# Comparison of Different Methods for the Meta‐Analysis of Diagnostic Test Accuracy Studies—A Simulation Study

**DOI:** 10.1002/bimj.70147

**Published:** 2026-07-02

**Authors:** Ferdinand V. Stoye, Olaf Raths, Alexander Hapfelmeier, Alexey Fomenko, Oliver Kuss, Annika Hoyer

**Affiliations:** ^1^ Biostatistics and Medical Biometry, Medical School OWL Bielefeld University Bielefeld Germany; ^2^ Institute of General Practice and Health Services Research, TUM School of Medicine and Health Technical University of Munich Munich Germany; ^3^ Institute of AI and Informatics in Medicine, TUM School of Medicine and Health Technical University of Munich Munich Germany; ^4^ Institute for Biometrics and Epidemiology, German Diabetes Center Leibniz Institute for Diabetes Research at Heinrich Heine University Düsseldorf Düsseldorf Germany

**Keywords:** diagnostic test accuracy studies, HADS‐A, ${\mathrm{HbA}}_{1c}$, meta‐analysis, ROC curve, simulation study

## Abstract

Meta‐analysis of diagnostic test accuracy studies aggregates information from multiple studies on sensitivity and specificity. Classical approaches select a single pair of sensitivity and specificity per study (single threshold methods, STM), ignoring additional information if studies report results on multiple diagnostic thresholds. Recently, models have been proposed that consider all available information and enable inference on all diagnostic thresholds (multiple threshold methods, MTM). We compare five STM and six MTM to each other in a simulation study, evaluating their performance in various situations. Covering a broad range of real‐life settings, we vary eight parameter dimensions in the data‐generation mechanisms, including continuous or ordinal outcome type of an index test, and different numbers of diagnostic thresholds available per study. While model performances are comparable regarding bias, empirical coverage, and convergence, we observe a logit GLMM of the MTM type to perform best in many situations. Model performances depend strongest on the outcome type, while the number of thresholds only has a minor impact. We thus find the main advantage of using MTM by getting threshold‐dependent estimates of sensitivity and specificity. Additionally, we illustrate differences between model estimates in two real‐data examples on diagnosing type 2 diabetes using the continuous biomarker HbA1c and screening for any anxiety disorder using the ordinal questionnaire HADS‐A. The applications reveal variations in model estimates within and between STM and MTM, which can be reduced by adjusting for the bias in the simulation settings resembling the real‐data situation most closely.

## 1 Introduction

Meta‐analysis is vital to translate the results of individual studies into statistically sound aggregates that guide researchers and practitioners in their decision‐making. For diagnostic test accuracy (DTA) studies reporting on sensitivity (probability that an individual with a target condition, determined by a reference standard test, tests positive) and specificity (probability that an individual without a target condition tests negative), meta‐analysis typically aims to estimate summary sensitivity and specificity, thereby characterizing the DTA (Macaskill et al. 2023). A key feature of diagnostic tests is that the diagnostic threshold, from which on patients are classified as test positives, can be chosen differently, impacting sensitivity and specificity. The higher the threshold is set, the lower the sensitivity and the higher the specificity will be if higher diagnostic test values indicate the target condition. This effect is commonly visualized using receiver operating characteristic (ROC) curves, which display pairs of 1‐specificity on the x‐axis and sensitivity on the y‐axis for multiple thresholds. Although DTA studies may report results on sensitivity and specificity for multiple thresholds (Choi et al. 2011; Wild et al. 2014; Negeri et al. 2024), most standard meta‐analysis methods only consider a single threshold from each available primary study (single threshold methods, STM) (Moses et al. 1993; Rutter and Gatsonis 2001; Reitsma et al. 2005; Chu and Cole 2006). They typically estimate (generalized) linear (mixed) models for transformations of one pair of sensitivity and specificity per study, including the study weights. Under certain assumptions (Rücker and Schumacher 2010), summary ROC (SROC) curves can subsequently be derived that describe the overall dependence between sensitivity and specificity. The area under the SROC curve (AUC) is finally computed as an overall DTA measure. To avoid information loss by ignoring additional results on multiple thresholds and to explicitly model the threshold effect, increased effort has been put into proposing novel methods that consider multiple thresholds per study (multiple threshold methods, MTM) in the past decade. These novel methods propose different approaches to include the diagnostic threshold, thereby allowing multiple entries per study to enter the model (Steinhauser et al. 2016; Hoyer and Kuss 2018; Hoyer et al. 2018; Frömke et al. 2022; Stoye et al. 2024).

While a number of MTM for the meta‐analysis of DTA studies have been proposed (see Section 2.4), systematic evaluation of these approaches in simulation studies that emulate a variation of realistic application scenarios remains scarce. Using the framework of Heinze et al. (2024), we have identified only a single phase III simulation study to explicitly compare existing MTM. In their study, Zapf et al. (2024) compared three MTM to each other: the logit linear mixed model (LMM) by Steinhauser et al. (2016), the Weibull accelerated failure time (AFT) model by Hoyer et al. (2018) and the semi‐parametric model by Frömke et al. (2022). While Zapf et al. conducted a comprehensive simulation study and identify the Weibull AFT model to be superior to the other approaches in some cases, their efforts leave two main questions unanswered: (1) DTA studies are often carried out on tests on an ordinal scale, e.g., using integer scores derived from questionnaires. Although there are case studies that compare MTM for meta‐analysis of ordinal scale DTA studies (Negeri et al. 2024; Benedetti et al. 2020), there does not appear to exist a systematic comparison of the performance of MTM using ordinal scale DTA studies by means of simulations. Zapf et al. only imposed data‐generating mechanisms with underlying continuous distributions for the diagnostic test values. Using ordinal instead of continuous scales may substantially impact the performance of MTM, because some methods assume a continuous distribution (Steinhauser et al. 2016; Hoyer and Kuss 2018), while others do not (Frömke et al. 2022). We thus deem it necessary to include ordinal data‐generating mechanisms in a simulation study to investigate potential effects. (2) As Zapf et al. only compared MTM to each other, it remains unclear how MTM compare to STM in their properties. It is desirable to quantify the positive (or negative) impact of using statistically more challenging and harder to implement MTM to simpler but established STM (Macaskill et al. 2023). Providing this quantification, along with a differentiated view on the scale on which a diagnostic test of interest is measured, is the aim of this simulation study.

Our study constitutes a phase III simulation study in the framework of Heinze et al. (2024), with the modification that we do not focus on a single method in our comparison. The study was pre‐registered in the Open Science Framework (Stoye et al. 2025). Throughout our study, we follow the ADEMP structure proposed by Morris et al. (2019). Therefore, we first introduce aims, data‐generating mechanisms, estimands, included models, and performance measures in Section 2. In Section 3, we continue by presenting the results of our simulation study, before illustrating the included models in two case studies on the diagnosis of type 2 diabetes using the continuously scaled biomarker ${\mathrm{HbA}}_{1c}$ and on the screening of any anxiety disorder using the HADS‐A questionnaire, which produces a discrete score, in Section 4. We conclude this paper by discussing our key findings and outlining possible future research questions in Section 5.

## 2 Methods

Throughout the paper, we use the following notation: we refer to the studies in a meta‐analysis by i, i=1,⋯,I, and to the individuals in study i using the index j, $j=1,\cdots ,N_{i}$. Each study i includes $N_{i}$ individuals, divided into $D_{i}$ diseased and $H_{i}$ non‐diseased persons, for which it reports results on $T_{i}$ diagnostic thresholds, $t_{ik}$, $k=1,\cdots ,T_{i}$. In the following, we use the terms target condition and disease interchangeably. The study‐specific disease prevalences are calculated as $p_{i}=D_{i}/N_{i}$. While for each individual a test value $y_{ij}$ is measured either on a continuous or an ordinal scale, we assume that studies only report the numbers of true positives (${\text{TP}}_{it_{ik}}$), false negatives (${\text{FN}}_{it_{ik}}$), false positives (${\text{FP}}_{it_{ik}}$), and true negatives (${\text{TN}}_{it_{ik}}$) for each reported threshold. From these values, empirical sensitivity (${\text{Se}}_{it_{ik}}={\text{TP}}_{it_{ik}}/\left({\text{TP}}_{it_{ik}}+{\text{FN}}_{it_{ik}}\right)$) and specificity (${\text{Sp}}_{it_{ik}}={\text{TN}}_{it_{ik}}/\left({\text{TN}}_{it_{ik}}+{\text{FP}}_{it_{ik}}\right)$) can be calculated for each diagnostic threshold.

### 2.1 Aims

Our simulation study aims to answer the following research questions:

- To what extent does the performance of STM and MTM (with respect to the performance measures defined in Section 2.5) depend on the measurement scale of a diagnostic test?
- Are MTM measurably superior to STM in cases where each primary study reports results on one or more diagnostic thresholds?
- Are STM measurably superior to MTM in cases where each primary study reports results on exactly one diagnostic threshold?
- Are there situations where specific models (between and among MTM and STM) perform best or outperform others?

Above we use the heuristic term model superiority based on the proportion of simulation settings, where one model outperforms another in terms of the performance measures (see Section 3.6). Although we design this study by incorporating a broad range of simulation settings to make it applicable to different kinds of DTA studies, we explicitly state that our aim cannot and must not be to make any absolute statements on the mathematical properties of the investigated models. We do not provide any proofs with this simulation and instead focus on empirical comparisons of model performances.

### 2.2 Data‐Generating Mechanisms

In a phase III study in the framework of Heinze et al. (2024), our aim is to compare methods regarding their estimation performance on data resembling real‐world settings, varying simulation settings according to different scenarios one might encounter when performing meta‐analyses in practice. Therefore, we motivate eight parameter dimensions that can vary in a meta‐analysis. In each dimension, we include two variants in our simulation, except for the number of diagnostic thresholds, where we include three variants. This leads to 384 simulation settings in total. The scenario space is summarized in Figure S3. We expect the simulation results in each dimension to potentially impact the method choice when performing a meta‐analysis of DTA studies in practice, depending on which setting most closely resembles the real‐data situation. For each simulation setting, we generate 1000 meta‐analysis datasets. We motivate the number of replications in each setting using a desired Monte Carlo standard error of 0.03 for the bias in AUC (Morris et al. 2019; Stoye et al. 2025). See Algorithm S1 for pseudocode of the data‐generating mechanisms.

#### 2.2.1 Number of studies per meta‐analysis:

Depending on the research question, the number of studies, I, for which data can be synthesized in a meta‐analysis of DTA studies, may vary strongly. We include a setting with fewer studies (I∼U[2;15], where U is the uniform distribution with bounds 2 and 15) and a setting with more studies (I∼U[7;50]) on average. In each simulation iteration, we draw I from the respective distributions and round to the nearest integer.

#### 2.2.2 Study size range per meta‐analysis:

Naturally, the number of individuals per study, $N_{i}$, in a meta‐analysis varies. The range of variation depends on the research question. For example, when the reference standard test is very costly, $N_{i}$ may be small on average. We include one setting with smaller studies on average ($N_{i}∼U\left[20;500\right]$) and one with larger studies ($N_{i}∼U\left[100;3000\right]$). After sampling from the uniform distributions, we round to the nearest integer.

#### 2.2.3 Prevalence range per meta‐analysis:

While DTA studies are often conducted in high‐risk populations where the disease prevalence is high, screening studies often deal with low‐prevalence populations (Obuchowski and Zhou 2002). Meta‐analyses aiming at the diagnostic accuracy of tests that are administered routinely in clinical settings may also rely on observational studies, where typically low prevalences are involved as well. We thus sample the true disease prevalence per study $p_{i}$ from two ranges, either $p_{i}∼U\left[0.01;0.1\right]$ or $p_{i}∼U\left[0.3;0.5\right]$.

#### 2.2.4 True diagnostic test accuracy:

The true DTA, measured by the AUC, varies for each diagnostic test. To include both a setting with medium and with high test accuracy, we either set AUC =0.75 or AUC =0.90. By varying the distribution parameters of the test value distributions of diseased and non‐diseased populations from which we generate the individual test values (see below and Supporting information A), we match the expected AUC to the specified value.

#### 2.2.5 Outcome type:

Most methods for the meta‐analysis of DTA studies are designed with continuous outcomes in mind. However, many applications deal with ordinal or discrete outcomes, such as discrete scores from a questionnaire, where it is especially common to include results on multiple diagnostic thresholds in a DTA study (Benedetti et al. 2020; Aktürk et al. 2025). We include a continuous setting with strictly positive test values and an ordinal setting. In the continuous settings, we sample test values from the four‐parametric generalized *F* distribution separately for diseased and non‐diseased populations, with an added bivariate Gaussian random effect to the location parameter to introduce heterogeneity. For the ordinal settings, we assume possible test values on the 0,1,⋯,21 scale and sample the number of persons in both populations per study having each test value from Dirichlet multinomial distributions, where the concentration parameter controls the induced heterogeneity between studies and acts as a (univariate) random effect. See Supporting information A for our concrete parameter choices for the distributions and visualizations of their probability density/mass functions.

#### 2.2.6 Study heterogeneity:

Study heterogeneity may depend on many measured or unmeasured factors, inter alia, on demographic differences in populations, differences in test administration, or time trends. To differentiate between situations where a smaller or larger heterogeneity is observed between studies, we include two settings in our simulation. For continuous outcomes, we vary the variance–covariance matrix of a bivariate Gaussian random effect in the data‐generating process for the test values. For ordinal outcomes, we vary the similarity between the study‐specific data‐generating probabilities for the ordinal scores by varying the concentration parameter in the Dirichlet multinomial distribution. The higher the concentration, the more similar the study‐specific probabilities are to the underlying probabilities, corresponding to less between‐study heterogeneity. See Supporting information B for our parameter choices and their motivation.

#### 2.2.7 Number of diagnostic thresholds reported per study:

Depending on the reporting culture in the respective research field and the research questions of DTA studies, studies may either report results on one or more diagnostic thresholds. To cover a broad field of applications, we include three variants in our simulation. Either all studies report on exactly one threshold, on between 1 and 5 thresholds, or on between 1 and 21 thresholds. The first variant is typical for fields where reporting on multiple thresholds is uncommon. The second variant is typical in cases where different thresholds may be motivated from a clinical point of view, for example, in the case of diagnosing (pre‐)diabetes (ElSayed et al. 2023). The third variant is chosen to align with the ordinal outcome type to cover cases, where some studies may report the results on every possible threshold in a questionnaire. In the second and third variants, we take a single threshold with 30% probability and otherwise sample the number of thresholds uniformly with boundaries described above, rounding to the next integer. The additional point mass on a single threshold recognizes that the number of thresholds reported per DTA study is unlikely to be univariately distributed, as an inflated number of (e.g., older) studies typically only report results on a single threshold. In all three cases, the diagnostic thresholds are sampled (for each study independently) without replacement out of the available threshold values. See Algorithm S1 for the exact mechanism. For the STM that only consider a single threshold in each study, we randomly select a single threshold per study after the threshold sampling is complete.

#### 2.2.8 Weight of a “standard“ threshold in each meta‐analysis:

For many diseases, official recommendations by health organizations on the choice of diagnostic threshold for a given diagnostic test are available. It can be assumed that the proportion of the recommended threshold in DTA studies included in a meta‐analysis will be elevated compared to other thresholds. To include this possibility in our simulation, we introduce a weight of a standard threshold in all three variants of number of thresholds reported on, which corresponds to the probability that this threshold is substituted for a randomly selected threshold out of the previously sampled ones. This weight is either set to w=0 or w=0.7. We set the standard threshold to 6.5 in the continuous setting and to 10 in the ordinal setting, inspired by the official recommendations on ${\mathrm{HbA}}_{1c}$ measured in percentage for the diagnosis of type 2 diabetes (World Health Organization 2011; ElSayed et al. 2023) and by common thresholds for questionnaires to screen for anxiety disorders (Spitzer et al. 2006; Aktürk et al. 2025; Fomenko et al. 2025).

### 2.3 Estimands

For all included methods, we primarily investigate three estimands: the AUC, the optimal sensitivity, and the optimal specificity of the diagnostic test (with respect to the data‐generating mechanisms and the performance measures defined in Section 2.5). AUC quantifies the overall diagnostic test performance, while sensitivity is concerned with the diseased, and specificity is concerned with non‐diseased individuals. Furthermore, MTM allow estimation of additional measures that describe the diagnostic test, i.e., optimal diagnostic threshold or the full ROC curve. We discuss these measures in our presentation of results, but refrain from using them for model comparison because they are not estimable from all models.

### 2.4 Models

We have identified 27 methods from 40 published articles (Moses et al. 1993; Rutter and Gatsonis 2001; Reitsma et al. 2005; Chu and Cole 2006; Steinhauser et al. 2016; Hoyer and Kuss 2018; Hoyer et al. 2018; Frömke et al. 2022; Stoye et al. 2024; Hoyer and Kuss 2019; 2020; Martínez‐Camblor 2017; Nikoloulopoulos 2015; Kardaun and Kardaun 1990; Littenberg and Moses 1993; Hasselblad and Hedges 1995; Hellmich et al. 1999; Kester and Buntinx 2000; Dukic and Gatsonis 2003; Poon 2004; Bipat et al. 2007; Arends et al. 2008; Hamza et al. 2009; Chu et al. 2010; Putter et al. 2010; Holling et al. 2012a; 2012b; Doebler et al. 2012; Chu et al. 2012; Charoensawat et al. 2014; Kuss et al. 2014; Riley et al. 2014; Schlattmann et al. 2015; Zapf et al. 2015; Doebler and Holling 2015; Guolo 2017; Jones et al. 2019; Guolo and To 2021; Guolo and Pesantez Cabrera 2023; Guolo 2025) that aim at meta‐analysis of DTA studies (see Table 1). We did not conduct a systematic literature search as the method papers introducing new methods for meta‐analysis are often hard to distinguish from application papers using the title and keywords. Instead, we performed a narrative review and identified further methods by “snowballing”. As this paper does not have the aim to be a comprehensive literature review on meta‐analysis of DTA studies, we did not deem a more systematic approach to be necessary. Out of the 27 methods, 12 are STM and 15 are MTM. In our simulation, we include 11 models, five STM (Moses et al. 1993; Reitsma et al. 2005; Chu and Cole 2006; Holling et al. 2012b; Nikoloulopoulos 2015) and six MTM (Steinhauser et al. 2016; Martínez‐Camblor 2017; Hoyer and Kuss 2018; Hoyer et al. 2018; Frömke et al. 2022; Stoye et al. 2024). Main exclusion criteria for omitting models are similarity to other included models (Kardaun and Kardaun 1990; Hellmich et al. 1999; Rutter and Gatsonis 2001; Kuss et al. 2014; Hoyer and Kuss 2019; Guolo and To 2021; Guolo 2025), inapplicability to include them in a simulation due to computational reasons (Hellmich et al. 1999; Rutter and Gatsonis 2001; Dukic and Gatsonis 2003; Jones et al. 2019), lack of available implementation or a concrete enough description in the article to allow manual re‐implementation (Kardaun and Kardaun 1990; Kester and Buntinx 2000; Dukic and Gatsonis 2003; Chu et al. 2012; Guolo and To 2021; Guolo and Pesantez Cabrera 2023), or model properties that make it impossible to compare the model to others in our simulation (Hasselblad and Hedges 1995; Poon 2004; Bipat et al. 2007; Hamza et al. 2009; Putter et al. 2010; Holling et al. 2012a; Schlattmann et al. 2015; Doebler and Holling 2015; Zapf et al. 2015). We briefly describe all identified models along with the reasons for their exclusion from the simulation (if applicable) in Supporting information C. In the following, we provide short descriptions of the included models and refer to the original publications for more details.

**TABLE 1:** Overview of relevant methods for meta‐analysis of DTA studies, ordered by year of publication. The column random effects indicates if the method includes random effects in its model formulation. Some methods include both fixed and random effects variants. The column multiple thresholds indicates if the method considers results on multiple thresholds per study, if available.

Method name	Author(s)	Year of publication	Random effects	Multiple thresholds	Included in simulation study
Basic LM	Kardaun and Kardaun	1990	×	×	×
SROC	Littenberg and Moses	1993	×	×	✓
	Moses et al.	1993			
StandDistance	Hasselblad and Hedges	1995	×/✓	×	×
HSROC	Hellmich et al.	1999	✓	×	×
	Rutter and Gatsonis	2001			
metaROC 2‐param.	Kester and Buntinx	2000	✓	✓	×
Unequal ordinal	Dukic and Gatsonis	2003	✓	✓	×
Multiple ordinal	Poon	2004	×	✓	×
Basic LMM	Reitsma et al.	2005	✓	×	✓
	Schlattmann et al.	2015			
Basic (G)LMM	Arends et al.	2008	✓	×	×
	Guolo	2017			
	Guolo	2025			
Basic GLMM	Chu and Cole	2006	✓	×	✓
	Chu et al.	2010			
Multinomial	Bipat et al.	2007	✓	✓	×
MREM	Hamza et al.	2009	✓	✓	×
	Guolo and Pesantez Cabrera	2023			
PCGF	Putter et al.	2010	✓	✓	×
SROC Lehmann	Holling et al.	2012a	✓	×	✓
	Holling et al.	2012b			
	Charoensawat et al.	2014			
SROC $t_{\alpha }$	Doebler et al.	2012	✓	×	×
	Doebler and Holling	2015			
BBM Sarmanov	Chu et al.	2012	✓	×	×
BBM copula	Kuss et al.	2014	✓	×	×
logit LMM	Riley et al.	2014	✓	✓	✓
	Steinhauser et al.	2016			
	Guolo and To	2021			
nPMA	Zapf et al.	2015	×	×	×
Normal/beta copula	Nikoloulopoulos	2015	✓	×	✓
nPSROC	Martínez‐Camblor	2017	×/✓	(✓)	✓
logit GLMM	Hoyer and Kuss	2018	✓	✓	✓
Weibull AFT	Hoyer et al.	2018	✓	✓	✓
Bayesian multinomial	Jones et al.	2019	✓	✓	×
Piecewise constant	Hoyer and Kuss	2019	✓	✓	×
Generalized F family	Hoyer and Kuss	2020	✓	✓	×
sPGR	Frömke et al.	2022	×	✓	✓
Discrete GLMM	Stoye et al.	2024	✓	✓	✓

#### 2.4.1 Single Threshold Methods

Classical methods for meta‐analyzing DTA studies rely on modeling transformations of sensitivity and specificity. In the SROC model of Moses et al. (1993); Littenberg and Moses (1993), which we include in our simulation, a linear model (LM) is estimated for $D_{i}=\text{logit}\left({\text{Se}}_{i}\right)-\text{logit}\left(1-{\text{Sp}}_{i}\right)$, by regressing on $S_{i}=\text{logit}\left({\text{Se}}_{i}\right)+\text{logit}\left(1-{\text{Sp}}_{i}\right)$:

(1) $$D_{i}=\alpha +\beta S_{i}+\varepsilon_{i},$$

where α,β are the parameters to estimate and $\varepsilon_{i}$, i=1,⋯,I, are the residuals. In this subsection, we leave out the threshold index $t_{i}$ as only a single threshold per study is considered. Studies are inverse‐variance weighted (Littenberg and Moses 1993). This model formulation is a univariate fixed effects model and directly models the shape of an SROC curve in the logit‐space.

Its bivariate extension to random effects (with a different transformation of sensitivity and specificity) was first proposed in a Bayesian framework (Rutter and Gatsonis 2001; Hellmich et al. 1999) and later in a (in case of no covariates equivalent) frequentist framework (Reitsma et al. 2005; Harbord et al. 2007; Arends et al. 2008). We include the LMM of Reitsma et al. (2005) (denoted as basic LMM in this paper), which directly models the bivariate endpoints $\text{logit}({\text{Se}}_{i})$ and $\text{logit}({\text{Sp}}_{i})$:

(2) $$\left(\begin{array}{c} \text{logit}({\text{Se}}_{i})\\ \text{logit}({\text{Sp}}_{i}) \end{array}\right)∼N\left(\left(\begin{array}{c} \theta_{\text{Se}}\\ \theta_{\text{Sp}} \end{array}\right),\Sigma \right),$$

where Σ is the variance–covariance matrix of the bivariate normal distribution and includes the study weights as precisions by having:

(3) $$\Sigma =\Sigma^{\prime }+C_{i}=\left(\begin{array}{cc} \sigma_{\text{Se}}^{2} & \sigma_{\text{Se,Sp}}\\ \sigma_{\text{Se,Sp}} & \sigma_{\text{Sp}}^{2} \end{array}\right)+\left(\begin{array}{cc} s_{\text{Se},i}^{2} & 0\\ 0 & s_{\text{Sp},i}^{2} \end{array}\right).$$

Here, $s_{\text{Se},i}^{2}=\frac{1}{D_{i}\cdot {\text{Se}}_{i}\cdot \left(1-{\text{Se}}_{i}\right)}$ and $s_{\text{Sp},i}^{2}=\frac{1}{H_{i}\cdot {\text{Sp}}_{i}\cdot \left(1-{\text{Sp}}_{i}\right)}$ are the variances of the estimated $\mathrm{logit}({\text{Se}}_{i})$ and $\mathrm{logit}({\text{Sp}}_{i})$. Backtransforming the estimated fixed parameters $\theta_{\text{Se}}$ and $\theta_{\text{Sp}}$ to the original scale of sensitivity and specificity yields a summary estimate of sensitivity and specificity, along with the corresponding bivariate normal distribution.

To fit the basic LMM, we first estimate study‐specific sensitivities and specificities, whose uncertainty is not considered in the final model. To alleviate this issue, Chu and Cole (2006); Chu et al. (2010) proposed a binomial generalized linear mixed model (GLMM) instead, denoted as basic GLMM in this paper. Although the authors consider different possible link functions, we restrict ourselves to the logit‐link.

The use of Gaussian random effects may be too restrictive on models that aim to synthesize results from studies in research fields, where different kinds of biases may influence results (e.g., publication bias). Copula models have been proposed to relax the assumption of Gaussian random effects by allowing a copula function to capture the dependence structure between variables, with the additional possibility to flexibly model the copula marginals with parametric univariate distributions (Kuss et al. 2014; Nikoloulopoulos 2015). We include the model of Nikoloulopoulos (2015) in the beta‐marginal variant, which models sensitivity and specificity on their original scale using beta distributions and links them via a bivariate Clayton copula (Clayton 1978).

Finally, methods have been proposed that aim to model the SROC curve directly in a flexible way (Holling et al. 2012b; 2012a; Doebler et al. 2012; Charoensawat et al. 2014; Doebler and Holling 2015). We include the model of Holling et al. (2012b), which proposes the Lehmann family

(4) $$p=u^{\theta },$$

with u∈[0,1] and θ>0 to model the SROC curve (Le 2006). θ is modeled with a univariate Gaussian random effect. After applying the natural logarithm, log(p)=θlog(u), plugging in p=Se, u=1−Sp and using normal approximations for log(p) and log(u), a model is estimated with adjusted profile likelihood maximization (Holling et al. 2012b). This model, denoted by SROC Lehmann in this paper, again constitutes a univariate model as in the case of the SROC model.

#### 2.4.2 Multiple Threshold Methods

Including a covariate for the diagnostic threshold $t_{i}$ into the meta‐analysis model may be seen as a straightforward solution to modeling multiple thresholds per study in a single model. Extensions to the basic LMM (Riley et al. 2014; Steinhauser et al. 2016) and the basic GLMM (Hoyer and Kuss 2018) have been proposed, with varying structures in the linear term for both fixed and random effects. Steinhauser et al. (2016) formulated a model for logit(Sp) and logit(1−Se) with (up to) a quadrivariate random effect, which we include in our simulation, termed logit LMM. We use the model variant with a trivariate random effect (*DICS, which is recommended in the original publication), allowing for different random effect intercepts for sensitivity and specificity, but not for different random slopes:

(5) $$\begin{array}{cc} \text{logit}({\text{Sp}}_{it_{ik}}) & =\beta_{01}+u_{01i}+\left(\beta_{02}+u_{2i}\right)\cdot t_{it_{ik}}+\varepsilon_{0it_{ik}},\\ \text{logit}(1-{\text{Se}}_{it_{ik}}) & =\beta_{11}+u_{11i}+\left(\beta_{12}+u_{2i}\right)\cdot t_{it_{ik}}+\varepsilon_{1it_{ik}},\\ \left(\begin{array}{c} u_{01}\\ u_{11}\\ u_{2} \end{array}\right) & ∼N\left(\left(\begin{array}{c} 0\\ 0\\ 0 \end{array}\right),\left(\begin{array}{ccc} \sigma_{01}^{2} & \rho_{1}\sigma_{01}\sigma_{11} & \rho_{2}\sigma_{01}\sigma_{2}\\ \rho_{1}\sigma_{01}\sigma_{11} & \sigma_{11}^{2} & \rho_{3}\sigma_{11}\sigma_{2}\\ \rho_{2}\sigma_{01}\sigma_{2} & \rho_{3}\sigma_{11}\sigma_{2} & \sigma_{2}^{2} \end{array}\right)\right). \end{array}$$

Here, $\beta_{01}$, $\beta_{02}$, $\beta_{11}$, and $\beta_{12}$ are the fixed model parameters. Backtransforming the estimated linear terms to the domain of sensitivity and specificity leads to smooth estimated SROC curves, expressed depending on the diagnostic threshold.

Like in the STM case, the logit LMM estimates study‐specific sensitivities and specificities in a first step, but now threshold‐specific. The logit GLMM, proposed in Hoyer and Kuss (2018), substitutes the LMM with a binomial GLMM in an analog to the STM case and can be formulated as:

(6) $$\begin{array}{cc} l({\text{Sp}}_{it_{ik}}) & =\beta_{01}+\beta_{02}\cdot t_{it_{ik}}+u_{1i}+\varepsilon_{0it_{ik}},\\ l({\text{Se}}_{it_{ik}}) & =\beta_{11}+\beta_{12}\cdot t_{it_{ik}}+u_{2i}+\varepsilon_{1it_{ik}},\\ \left(\begin{array}{c} u_{1}\\ u_{2} \end{array}\right) & ∼N\left(\left(\begin{array}{c} 0\\ 0 \end{array}\right),\left(\begin{array}{cc} \sigma_{1}^{2} & \rho \sigma_{1}\sigma_{2}\\ \rho \sigma_{1}\sigma_{2} & \sigma_{2}^{2} \end{array}\right)\right), \end{array}$$

where l is the link function used in the binomial GLMM. We use the logit‐link in our simulation and term this model logit GLMM. Note that the logit GLMM only uses a bivariate instead of the trivariate random effect in the logit LMM. We follow the original implementation by Hoyer and Kuss (2018) but using a different random effect structure would be possible in principle.

A fundamentally different approach is proposed by Hoyer et al. (2018), who rearrange the data reported by primary studies into bivariate interval‐censored time‐to‐event data. Here, the diagnostic test value acts as a timescale and the numbers of individuals with an underlying test value in the intervals between thresholds are taken as event counts. The resulting data structure is modeled as an AFT model, estimating the probability to be tested positive in the diseased (sensitivity) and the non‐diseased population (1‐specificity). A bivariate random effect is included in the linear predictor of the logarithmized test values:

(7) $$\begin{array}{cc} \log(y_{Hij}) & =\beta_{H}+u_{Hi}+\varepsilon_{Hij},\\ \log(y_{Dij}) & =\beta_{D}+u_{Di}+\varepsilon_{Dij},\\ \left(\begin{array}{c} u_{H}\\ u_{D} \end{array}\right) & ∼N\left(\left(\begin{array}{c} 0\\ 0 \end{array}\right),\left(\begin{array}{cc} \sigma_{H}^{2} & \rho \sigma_{H}\sigma_{D}\\ \rho \sigma_{H}\sigma_{D} & \sigma_{D}^{2} \end{array}\right)\right). \end{array}$$

While Hoyer et al. (2018) used different parametric distributions (Weibull, log‐logistic, log‐normal), we only use the Weibull distribution for the diagnostic test values, overall leading to a Weibull AFT model which we include in our simulation. Estimated summary sensitivities (1‐specificities) can be computed from the population‐specific Weibull survival functions.

Stoye et al. (2024) adapted the time‐to‐event approach but avoid a parametric distributional assumption for the diagnostic test values by modeling the discrete hazard of study participants to lie in the intervals between subsequent thresholds. The resulting model can be embedded as a binomial GLMM with one categorical parameter for each unique threshold in the dataset, separately for the diseased and non‐diseased population and a bivariate random effect:

(8) $$\begin{array}{cc} l(\lambda_{\mathrm{ij}t_{\mathrm{ik}}}) & =h(\alpha_{it_{\mathrm{ik}}}+x_{1\mathrm{ij}}^{⊤}\cdot \beta_{1}+x_{1\mathrm{ij}}^{⊤}\cdot \beta_{2}\cdot x_{\mathrm{Hij}}+u_{\mathrm{Hi}}\cdot x_{\mathrm{Hij}}\\ & \;+u_{\mathrm{Di}}\cdot \left(1-x_{\mathrm{Hij}}\right)+\varepsilon_{\mathrm{ij}t_{\mathrm{ik}}}),\\ \left(\begin{array}{c} u_{H}\\ u_{D} \end{array}\right) & ∼N\left(\left(\begin{array}{c} 0\\ 0 \end{array}\right),\left(\begin{array}{cc} \sigma_{H}^{2} & \rho \sigma_{H}\sigma_{D}\\ \rho \sigma_{H}\sigma_{D} & \sigma_{D}^{2} \end{array}\right)\right), \end{array}$$

with baseline hazard $\alpha_{it_{ik}}$, fixed parameter vectors $\beta_{1}$, $\beta_{2}$, indicator vectors $x_{1ij}$ for the interval in which the test value of individual j in study i lies and scalar $x_{Hij}$ that indicates if it is non‐diseased. After model estimation, the summary sensitivities and 1‐specificities are computed from the discrete survival functions using the estimated fixed model parameters. In our simulation, we use the asymmetric complementary‐log‐log link function, as suggested by Stoye et al. (2024) and term the resulting model discrete GLMM.

There have also been non‐parametric models proposed in meta‐analysis of DTA studies. Martínez‐Camblor (2017) proposed a fully non‐parametric model to estimate the SROC curve by a weighted average over the estimated individual study ROC curves, which are linearly interpolated through the known points (0,0) and (1,1). To estimate the individual ROC weights, both a fixed‐effect and a random‐effect approach are used. We choose the random‐effects variant (DerSimonian and Kacker 2007) and include the resulting model in our simulation, referred to as nPSROC. Note that this model does not allow inference on the diagnostic threshold. We still include it in the MTM part of the selected models as it estimates a full SROC curve and can handle multiple thresholds per study.

Finally, we include the semi‐parametric model of Frömke et al. (2022) in our simulation, termed sPGR. This model generates pseudo‐observations for each person in each study from uniform distributions using the information on the interval between thresholds in which the individual test values lie. Subsequently, all sampled values are replaced by their global midranks $R_{ijk}$, k=D,H, indicating the disease status and the summary AUC is estimated as

(9) $$\hat{\text{AUC}}=\frac{1}{2}+\frac{1}{\sum_{i=1}^{K}N_{i}}\left({\bar{R}}_{D}-{\bar{R}}_{H}\right),$$

where ${\bar{R}}_{H}$, ${\bar{R}}_{D}$ denote the mean ranks of the (non‐)diseased individuals. Frömke et al. (2022) proposed to estimate summary sensitivities and specificities in a similar fashion, replacing the observations of (non‐)diseased individuals with one‐point distributions for the sensitivity (specificity).

### 2.5 Performance Measures

We assess the overall model performance by means of bias, empirical coverage, and model convergence.

Bias, the mean difference between estimated values and the true value (determined by the data‐generating mechanisms), is assessed four‐fold, with respect to the estimated AUC, the estimated summary sensitivity, the estimated summary specificity, and (if applicable) the estimated optimal diagnostic threshold. To obtain these estimates, different approaches are taken for STM and MTM. While most STM give summary estimates of sensitivity and specificity directly, we use the optimal sensitivity and specificity with respect to the maximum (unweighted) Youden‐index (Youden 1950), ${\text{argmax}}_{t}\left({\text{Se}}_{t}+{\text{Sp}}_{t}-1\right)$, for the MTM, which in principle provide estimates for the whole SROC curve. Conversely, MTM allow direct estimation of the AUC from the estimated SROC curve. Estimation of the SROC curves varies for the STM. While the univariate SROC and SROC Lehmann model supply a direct relation to an estimated SROC curve, we use the additional assumption that studies report results based on the optimal Youden‐index proposed in Rücker and Schumacher (2010) for the basic LMM and GLMM to compute the SROC curves. In the case of the beta copula model, we compute the sensitivities (1‐specificities) from the quantile function of the estimated beta‐distribution of the diseased (non‐diseased) population. The true optimal sensitivities and specificities of the data‐generating processes are also computed based on the unweighted Youden‐index, whereas the true AUC is computed numerically, considering all available true sensitivities and specificities (see Supporting information A).

Empirical coverage is estimated by the proportion of cases where the 95% Wald‐type confidence interval (CI) of estimated summary sensitivity/specificity encloses the true optimal values. We do not consider empirical coverage in AUC or diagnostic threshold because not all included methods provide a Wald‐type CI for these performance measures. Computing non‐parametric bootstrap CIs (as done in Section 4) was not deemed feasible in our simulation.

Model convergence probabilities can be estimated as the proportion of converged simulation runs out of the 1000 replicates in each simulation scenario. Here, we use the same convergence criteria as proposed in the original implementations of the methods.

## 3 Simulation Results

In this section, we report the results from our simulation study. After we begin by giving details on model estimation, we briefly describe the overall model performance regarding bias, empirical coverage, and convergence. We then present observed effects on model performance when changing the underlying simulation parameters, before giving results specific to the four aims of this simulation study, defined in Section 2.1.

### 3.1 Model Estimation

We estimated all 11 included models on all datasets generated using the mechanisms explained in Section 2.2 and recorded estimated bias of AUC, optimal diagnostic threshold (if applicable), optimal sensitivity, and specificity using the criteria defined in Section 2.5, estimated standard errors of optimal sensitivity and specificity for Wald‐type CI computation, and an indicator of model convergence. If available, we used existing implementations in the form of R‐packages and implemented the methods ourselves otherwise. Unless noted otherwise, all calculations and implementations were made in R v.4.5.3 (R Core Team 2025) on a Linux virtual machine with a 28‐core 2.2 GHz AMD EPYC CPU.

In more detail: We manually implemented the SROC model based on the descriptions in Moses et al. (1993) (see Supporting information D for a derivation of the standard error of estimated sensitivity) and the basic GLMM based on code provided in Partlett and Takwoingi (2021), using lme4 v.2.0‐1 (Bates et al. 2015). For the basic LMM and the SROC Lehmann model, we used the implementations in the R‐package mada (Doebler and Sousa‐Pinto 2022) and for the beta copula model the package CopulaREMADA (Nikoloulopoulos 2025). In case of the MTM, we used diagmeta (Rücker et al. 2022) for the logit LMM, nsROC (Fernandez 2018) for the nPSROC model, diagacc (Weber and Zapf 2023) for the sPGR model, and metaROC (Stoye and Raths 2025b) for the discrete GLMM. We manually implemented the logit GLMM based on the description in Hoyer and Kuss (2018) using lme4 v.2.0‐1, and used SAS code provided in Hoyer et al. (2018) to estimate the Weibull AFT model in SAS 9.4 for Windows, Cary, NC, USA. Note that the packages diagacc and metaROC are currently not listed on CRAN. We include archives of the used package versions in the Supporting information to this paper to enable full reproducibility of our results.

All model implementations and simulation code are supplied at https://gitlab.ub.uni‐bielefeld.de/stoyef/metaROC/‐/tree/simstudy as a branch to the R‐package metaROC.

Using the described implementations, it was not possible to estimate the following quantities for certain models: due to being univariate models, there are no standard errors for specificity available for the SROC and SROC Lehmann model. Additionally, the nPSROC model does not supply standard errors for specificity. Therefore, Wald‐type CIs for specificity cannot be computed and coverage in specificity cannot be assessed for these three models. The optimal diagnostic threshold is estimated by all MTM, except the nPSROC model, which ignores the concrete threshold values. The logit LMM internally only allows to be estimated if at least one primary study reports on more than one threshold. It therefore could only be estimated in 256 out of the 384 simulation settings, where this condition was met.

### 3.2 General Results on Bias, Empirical Coverage, and Convergence

The simplest approach to evaluating overall model performance is to pool the results over all 384 simulation settings. However, this would ignore potential differences between specific simulation settings. For example, it may happen that a model systematically overestimates the AUC for certain values in a varied parameter dimension, while underestimating it in others. Pooling over all settings would ignore this and wrongly identify the model as having low median bias.

Table S3 shows simulation results of all performance measures, pooled over all simulation settings. Unless noted otherwise, we report our simulation results (in tables and figures) ordered chronologically by year of model publication. For the bias in specificity, we omitted all absolute standardized biases larger than 10 to avoid numerical artifacts. Tables S4 and S5 stratify the pooled results by outcome type, revealing that only in the case of convergence, the models performing best overall also perform best in both subsets of simulation settings. Therefore, it is necessary to report results depending on the simulation settings. A much more detailed picture of the variation in model performance depending on the setting is given by heatmaps in Figure S4, scatterplots in Figure S6, and by interactive nested loop plots (Rücker and Schwarzer 2014) in Figure I1 in the Supporting information, where detailed results on all 384 settings are shown.

The AUC is consistently and substantially underestimated by the nPSROC and sPGR model (see Figures S4, S6, and I1). All other models overestimate the AUC in some settings, while underestimating it in others. The optimal sensitivity is underestimated in all settings by the beta copula and the SROC Lehmann model, while the sign of the sensitivity bias of other models varies depending on the setting. The optimal specificity is overestimated by the discrete GLMM and the SROC Lehmann model in all settings. All other models vary in their sign of specificity bias. The absolute bias in the optimal diagnostic threshold (which can only be assessed for the logit LMM, logit GLMM, Weibull AFT, sPGR, and discrete GLMM) is highest for the discrete GLMM, which underestimates the optimal threshold in almost all settings. The logit LMM, logit GLMM, and sPGR models overestimate the threshold in most settings, but in a much lower magnitude.

Suppose we define a satisfying model performance by an absolute bias of less than one percentage point. In that case, satisfying performance is reached in between 0% and 66.9% of the simulation settings for the bias in AUC, in between 0% and 37.8% of the settings for the bias in optimal sensitivity, and in between 0% and 45.8% of settings for the bias in optimal specificity, depending on the used model (see Table 2). In Section 3.3, we present more nuanced results, depending on the simulation settings.

**TABLE 2:** Proportions of simulation settings where satisfying performance was reached. Performance was deemed satisfying in cases where the absolute bias of a model was smaller than 1 percentage point, the empirical coverage was at least 93.6%, or the proportion of converged models out of 1000 replicates in a setting was at least 90%. No results on the bias in optimal diagnostic thresholds are included as the thresholds are measured on different scales for the continuous and ordinal outcome settings.

	Percent of absolute bias <1%			Percent of empirical coverage ≥93.6%		
Model	AUC	sensitivity	specificity	sensitivity	specificity	Percent of convergence ≥90%
SROC (Moses et al. 1993)	51.8	37.8	1.8	98.4	N/A	100
Basic LMM (Reitsma et al. 2005)	53.9	1	12	8.1	11.7	100
Basic GLMM (Chu and Cole 2006)	69.3	3.1	13.3	6	6.5	97.1
SROC Lehmann (Holling et al. 2012b)	27.9	0	0	0	N/A	100
Beta copula (Nikoloulopoulos 2015)	36.2	0	4.2	0	0	91.9
Logit LMM (Steinhauser et al. 2016)	60.5	18.8	43	79.7	45.3	66.7 ^a^
nPSROC (Martínez‐Camblor 2017)	0.5	4.9	17.7	0	N/A	100
Logit GLMM (Hoyer and Kuss 2018)	59.4	37.2	45.8	95.1	74.7	91.7
Weibull AFT (Hoyer et al. 2018)	66.9	4.9	39.6	32.6	4.4	49
sPGR (Frömke et al. 2022)	0	1.3	10.4	0	0	100
Discrete GLMM (Stoye et al. 2024)	17.4	8.1	0	43.5	91.9	43.8

a The logit LMM does not allow model estimation if all studies only report on a single threshold (which is the case in a third of the simulation settings). The model reached 90% convergence in all settings where it was estimated.

After running 1000 replicates in each simulation setting, we would expect the empirical coverage to be in the interval [0.936;0.964] for a correctly specified model (Hoyer and Kuss 2018). Following Morris et al. (2019), we also accept confidence validity, which is fulfilled if at least 95% of the CIs include the true value. Combining both concepts, satisfying empirical coverage (of at least 93.6%) for optimal sensitivity is reached in between 0% (SROC Lehmann, beta copula, nPSROC, and sPGR models) and 98.4% (SROC model) of the simulation settings. Correspondingly, satisfying coverage is reached for the optimal specificity in between 0% (beta copula and sPGR models) and 91.9% of settings for the discrete GLMM. See Table 2 for the values of all models. Note that coverage in specificity cannot be assessed for the SROC, the SROC Lehmann, and the nPSROC models.

Satisfying convergence probability may depend on the application and user. Using the implemented convergence mechanisms in the included models, convergence was estimated to be at least 90% in between 43.8% (discrete GLMM) and 100% of the simulation settings. All models except the discrete GLMM and Weibull AFT model reached 90% in more than 91% of the settings (see Table 2).

Combining the definitions on satisfying performance defined above, only the SROC model (1/384 settings), the logit LMM (4/256 settings), and the logit GLMM (81/384 settings) fulfill all six evaluated performance measures satisfyingly in the same simulation setting.

### 3.3 Effects of Parameter Dimensions

**FIGURE 1:**
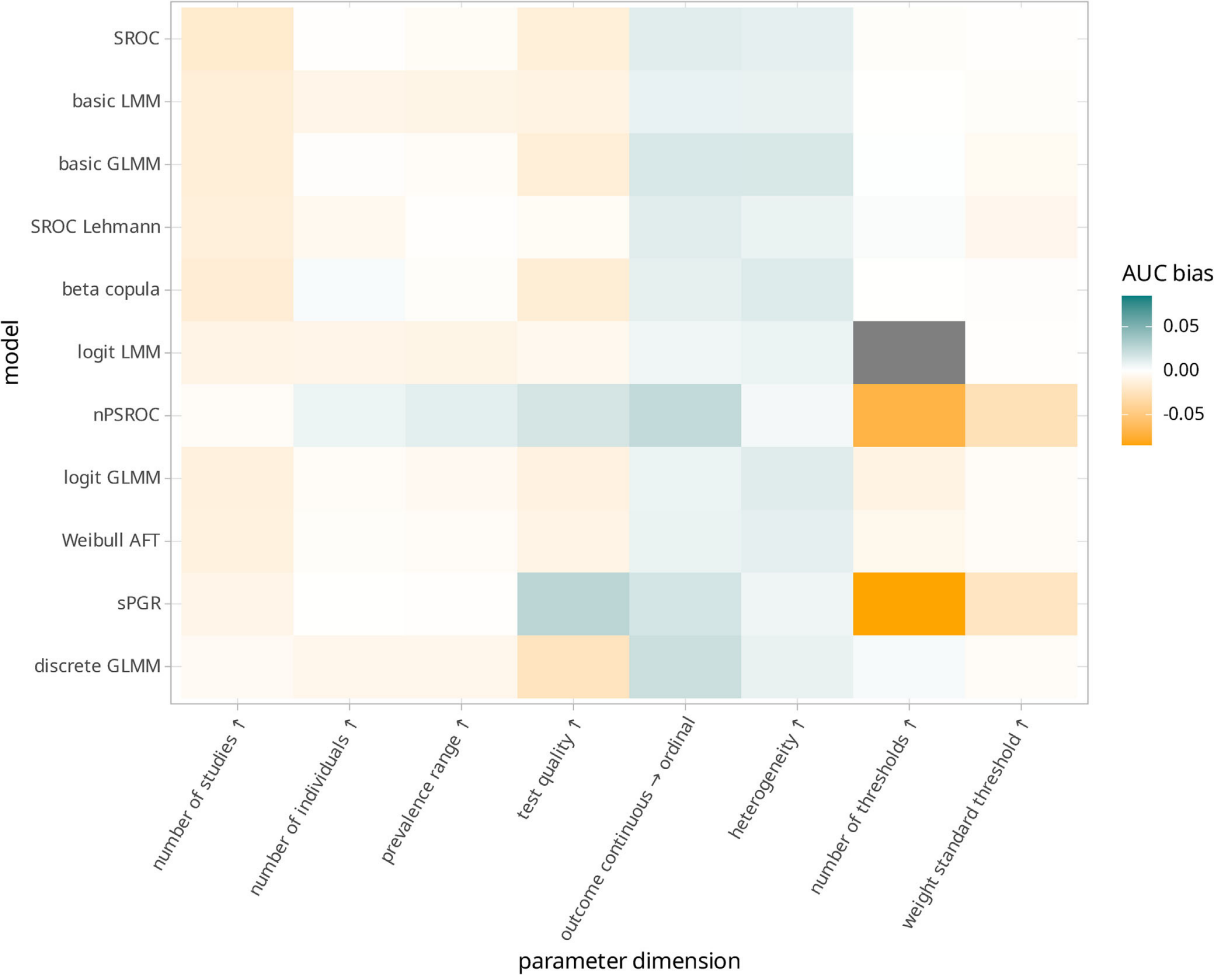
Heatmap of mean change in absolute AUC bias when varying the parameters in the data‐generating mechanism. The changes are denoted on the x‐axis and displayed ceteris paribus. For the number of thresholds, the change from $T_{i}=1$ to $1\leq T_{i}\leq 21$ is visualized. AUC, area under curve.

The focus of our simulation study's design is to analyze possible effects on model performance if underlying simulation parameters change. In Section 2.2, we motivated eight parameter dimensions that can vary in real‐data situations. We present key effects resulting from changes in these dimensions in the following. The effects on the average change in AUC bias when varying the parameters are visualized as a heatmap in Figure 1. More robust methods regarding the change of a parameter possess a lighter color on the heatmap. See Figures S7– S12 for corresponding heatmaps of the other performance measures. Additionally, we provide interactive nested loop plots for the log‐relative change in performance when changing the parameters, depending on the setting, in Figures I2– I11.

Overall, one might expect the overall bias to be reduced when more data are provided, by increasing the number of studies in a meta‐analysis, the number of individuals per study, or the number of reported thresholds per study. On the other hand, an increase in heterogeneity is expected to increase model biases.

AUC bias (see Figure 1) is most strongly impacted by increasing the number of thresholds reported per primary study. Here, the absolute bias of the nPSROC and the sPGR model is reduced substantially. These two models behave differently from all others, which are largely consistent and exhibit increased AUC bias for increased heterogeneity and a switch to an ordinal outcome, while decreased bias for increased numbers of studies, individuals, prevalence, DTA, and weight of a standard threshold. Notably, the STM's bias is not impacted strongly when increasing the number of thresholds reported per study, except for a slight increase in specificity bias for the basic LMM, basic GLMM, and beta copula model. The changes in AUC bias are mostly comparable to sensitivity and specificity (Figures S7 and S8). The largest difference between the three measures is the much stronger observed increase in specificity bias for the STM than for the MTM when switching from a continuous to an ordinal outcome, which is not observed in AUC or sensitivity bias.

Absolute bias in the optimal diagnostic threshold (Figure S9) is impacted most strongly in the case of the discrete GLMM, where it increases substantially when switching to an ordinal outcome or when increasing the number of thresholds per study. The other MTM are impacted to a lesser extent, following the overall pattern described above.

Changing the underlying parameters has a large effect on the empirical coverage of sensitivity and specificity (up to 40%, see Figures S10 and S11). However, patterns are less clear than in the case of the change in bias. Most notably, the logit LMM and logit GLMM are robust in their coverage of the optimal sensitivity, while the discrete GLMM is mostly robust regarding the optimal specificity. Increasing the weight of a standard diagnostic threshold from w=0 to w=0.7, results in lower empirical coverage for the basic LMM and basic GLMM, when isolating the simulation settings where studies report a single threshold. In contrast, the bias remains invariant to the change in weight (see Figures S17 and S18 for a more detailed presentation using boxplots).

Apart from the fact that the logit LMM cannot be estimated for a single threshold per study, we observe only minor changes in convergence behavior (see Figure S12). The only models impacted by a change in data‐generating parameters are the discrete GLMM and Weibull AFT model, which also perform worst regarding convergence overall as indicated by Table 2.

Next to visually inspecting effects of the parameter dimensions, we also identify the most relevant dimensions regarding each performance measure by using model‐based recursive partitioning (Zeileis et al. 2008; Hothorn and Zeileis 2015). The three most relevant dimensions, along with boxplots of the model‐specific performance, are shown in Figure S13 and S14. Identified as the most relevant parameter dimension is the outcome type, which is selected for all compared performance measures, followed by the number of thresholds, the true DTA, the weight of a standard threshold, and the heterogeneity between studies. Prevalence range and study size range were not selected for any of the performance measures. While this approach is able to detect the most relevant parameter dimensions to model performance, it does not reveal possible systematic dependencies or directions of changes in model performance. These are investigated in more detail in Sections 3.4 and 3.5.

### 3.4 Comparison of Continuous and Ordinal Outcome Settings

**FIGURE 2:**
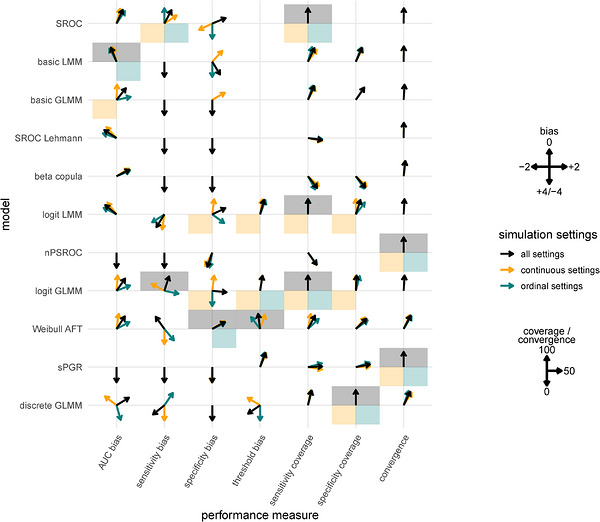
Arrow map of performances of all models, either pooled over all, all continuous, or all ordinal simulation settings. The arrow tips indicate performance (the higher the tip, the better the performance). For AUC, sensitivity, and specificity bias, the lowest point in performance is set to an absolute bias of four percentage points to better distinguish well‐performing models from each other. In case of the bias in the optimal threshold, values are scaled so that the worst performing model points to the bottom. For each performance measure, the best performing models are highlighted by the colored squares (top of the arrow: all settings; bottom‐left of the arrow: continuous settings; bottom‐right of the arrow: ordinal settings). All coverages of at least 95% are taken to be optimal, leading to arrows that point to the top. AUC, area under curve.

The first aim of this simulation, defined in Section 2.1, deals with exploring dependencies of model performances on the underlying measurement scale of the diagnostic test. In the context of our study, this means comparing the results of simulation settings using a continuous outcome with settings using an ordinal outcome. Figure 2 compares the performance of all 11 models pooled over all settings and stratified by the two outcome types using an arrow map. The corresponding numerical values can be found in Tables S3– S5.

Absolute AUC bias increases for all models in the case of ordinal, compared to continuous outcomes. This may point either to differences in the data‐generating processes or a worse performance on ordinal‐scaled diagnostic tests. Substantial dips in performance are observed for the discrete GLMM (however, note that the discrete GLMM underestimates the AUC in the continuous settings but overestimates the AUC in the ordinal settings), and to a lesser extent for the basic GLMM, logit GLMM, and Weibull AFT model. The differences between outcomes get more nuanced regarding the other performance measures. Absolute sensitivity bias is only low for the SROC and logit GLMM when a continuous outcome is used, and for the SROC and discrete GLMM when an ordinal outcome is observed. Absolute specificity bias is low for the basic LMM, basic GLMM, logit LMM, logit GLMM, and Weibull AFT model when a continuous outcome is used, but only for the Weibull AFT model when an ordinal outcome is used. Comparing the absolute bias in optimal diagnostic threshold is not easily possible, as continuous and ordinal outcomes are measured on different scales. Empirical coverage and model convergence are largely independent of the outcome. Substantial variations in performance can only be observed for the coverage in sensitivity of the Weibull AFT model and the specificity coverage of the logit LMM, which are higher for the continuous outcome.

Overall, model bias is sensitive to the measurement scale of the diagnostic test, whereas empirical coverage of optimal sensitivity and specificity and model convergence remain stable. Systematic differences between STM and MTM cannot be observed. The best performing model overall depends on the performance measure used to compare the models.

### 3.5 Comparison of Multiple Threshold Methods and Single Threshold Methods Depending on the Number of Diagnostic Thresholds

**FIGURE 3:**
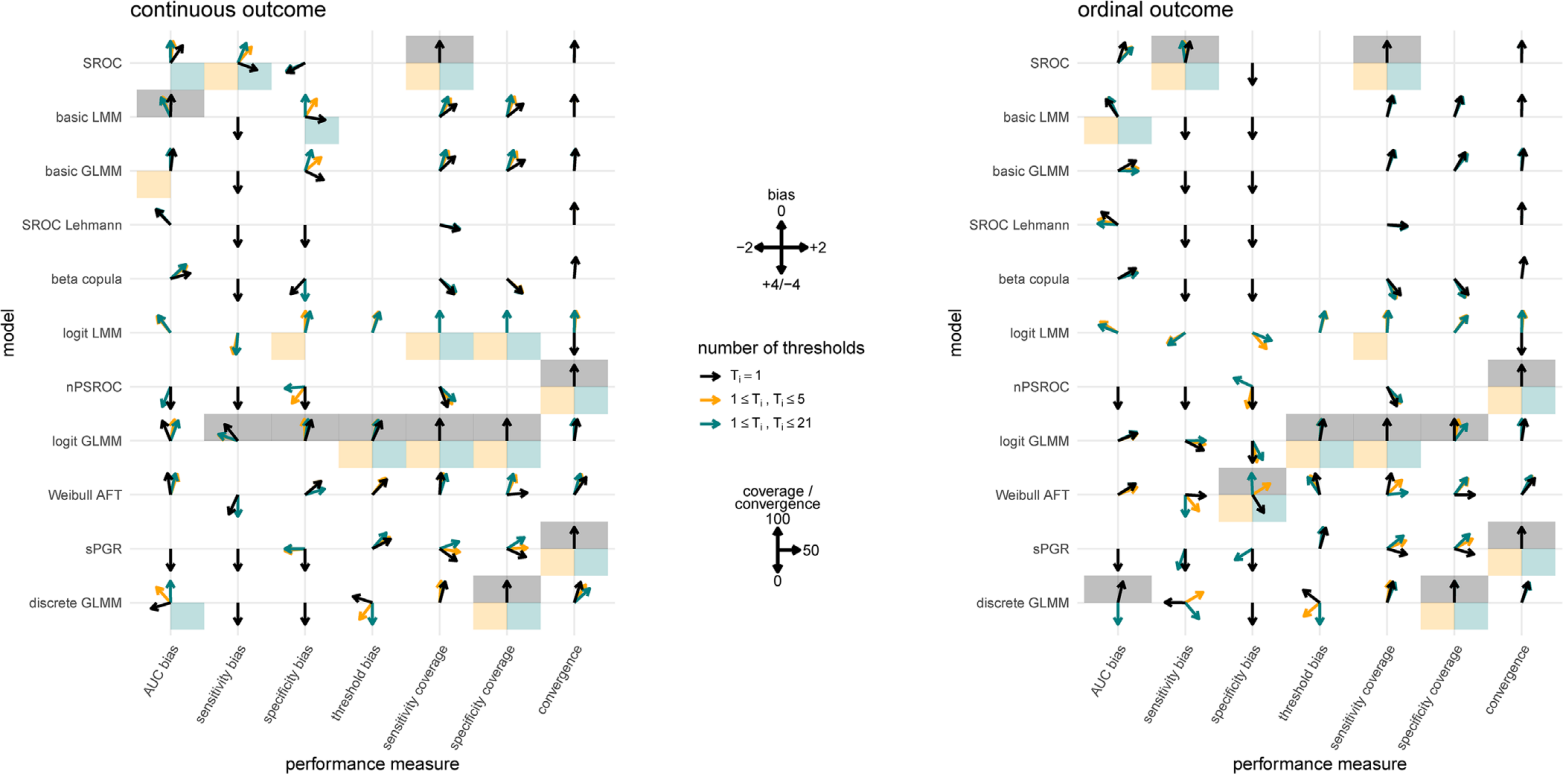
Arrow maps of performances of all models, stratified by the range in number of thresholds $T_{i}$ included in the data generation and by outcome type. The arrow tips indicate performance (the higher the tip, the better the performance). For the AUC, sensitivity, and specificity bias, the lowest point in performance is set to an absolute bias of four percentage points to better distinguish well‐performing models from each other. In case of the bias in the optimal threshold, values are scaled so that the worst performing model points to the bottom. For each performance measure, the best performing model(s) are highlighted by the colored squares (top of the arrow: $T_{i}=1$; bottom‐left of the arrow: $1\leq T_{i}\leq 5$; bottom‐right of the arrow: $1\leq T_{i}\leq 21$). All coverages of at least 95% are taken to be optimal, leading to arrows that point to the top. AUC, area under curve.

We also aim to compare STM and MTM to each other regarding their behavior when estimated to meta‐analysis datasets with or without more than a single threshold per study (second and third aim in Section 2.1). In a similar fashion to Section 3.4, Figure 3 shows two arrow maps of model performances, stratified by the three variations in ranges of number of thresholds reported on per study and visualized separately for continuous and ordinal outcome settings.

STM are designed to be estimated on meta‐analysis data that include results on a single threshold per individual study. In cases where more information was available, we randomly selected results on one of the thresholds per study. MTM, on the other hand, use all available information and are estimated on the full datasets. This leads to the expectation that MTM may perform better in simulation settings where data on one or more thresholds are available, whereas STM may perform better in settings where only data on a single threshold are available. However, this presumption is not supported by our simulation results shown in Figure 3. Based on our results, there does not appear to be a clear structure to the change in bias depending on the number of thresholds available. Further, the STM do not clearly outperform the MTM where data on a single threshold are available in each study and the MTM do not outperform the STM where data on more than one threshold are available. This is evident by comparing the highlighted arrows in Figure 3 that indicate the best performing model(s) for each performance measure.

Empirical coverage either remains stable when varying the number of thresholds during data generation or increases with increasing number of thresholds reported on, with some exceptions (see Figure 3). We do not observe a structural difference between STM and MTM, as well as for model convergence.

In summary, absolute model performance does not depend systematically on the number of thresholds used during data generation for all models. However, depending on the concrete setting they are still a relevant factor to model performance (see Section 3.3), necessitating individual performance comparisons for a given data structure (see Figure I1).

### 3.6 In What Situations do Models Outperform Others?

The nPSROC and sPGR models are outperformed by others in almost all situations, except regarding model convergence. Thus, we omit these two models in the remainder of this section. For the other models, no such clear statement is possible. To systematically compare the remaining models, we investigate the marginal behavior of all parameters that vary during data generation. That is, for all 2·7+3=17 variations of the eight parameter dimensions described in Section 2.2, we compute the proportions where one of the models outperforms another in each performance measure. We define a model to outperform another if it performs better in more than 80% of the simulation settings under investigation. We compute these numbers by averaging over the proportions in the case of (AUC, sensitivity, and specificity) bias, or (sensitivity and specificity) coverage. If this is the case, we deem a model superior to another in a specific parameter dimension, independent of the simulation setting. We omit the bias in the diagnostic threshold here to preserve comparability between STM and MTM.

Based on this definition of superiority, no model outperforms another in all parameter dimensions when combining bias, coverage, and convergence. However, when only considering (AUC, sensitivity, and specificity) bias, the logit GLMM outperforms the SROC Lehmann and discrete GLMM, and the Weibull AFT model outperforms the SROC Lehmann model. Furthermore, regarding (sensitivity and specificity) coverage, the basic LMM, basic GLMM, logit GLMM, Weibull AFT, and discrete GLMM all outperform the beta copula model. Additionally, the logit GLMM outperforms the basic GLMM. Regarding convergence, the Weibull AFT and discrete GLMM are outperformed by all other models, except for the beta copula model in the case of the discrete GLMM.

**FIGURE 4:**
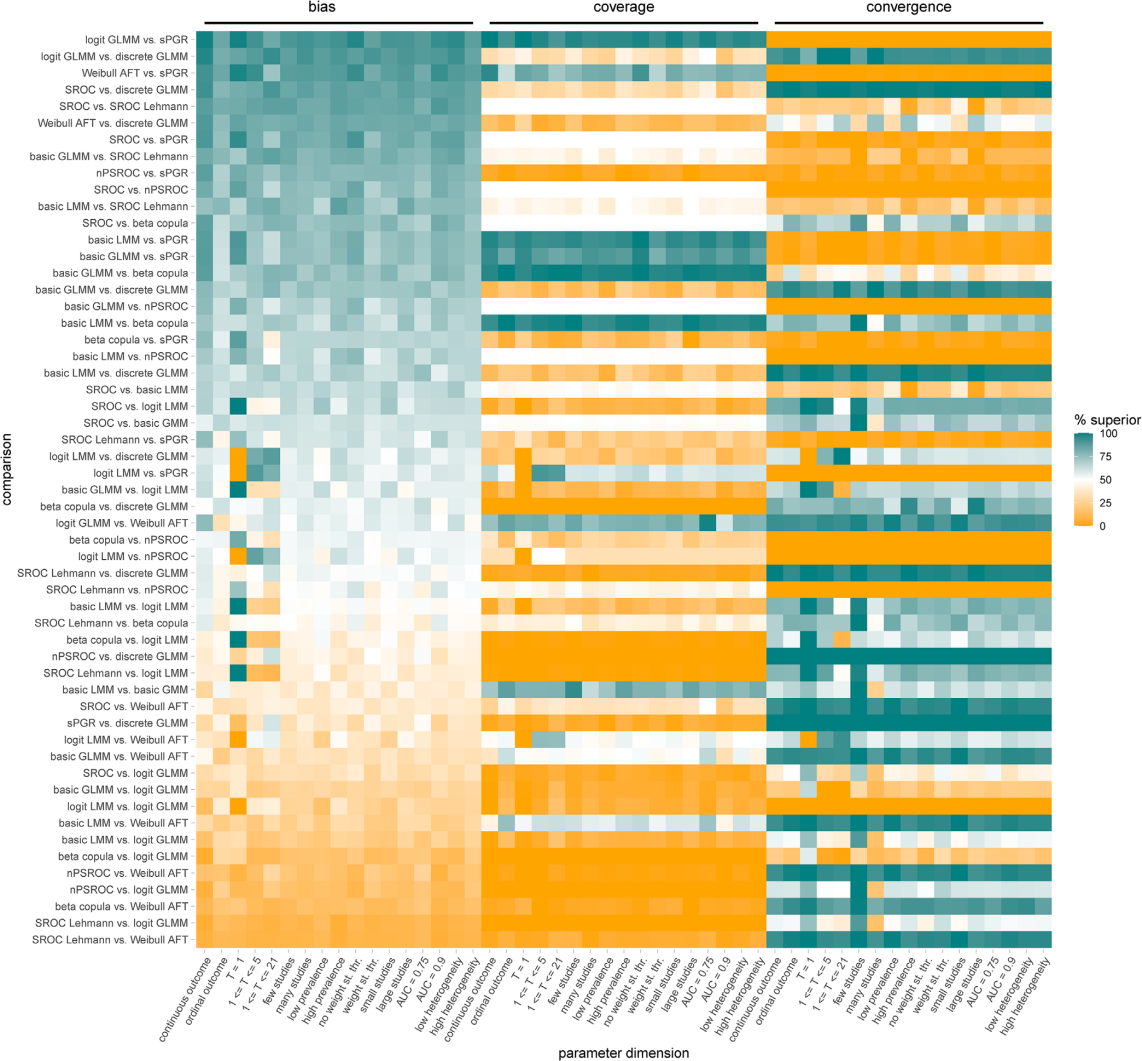
Heatmap of proportions where each model outperforms another, regarding the average of AUC, sensitivity, and specificity bias, the average of sensitivity and specificity coverage, and convergence. In each of the three blocks, all settings with the parameter dimension detailed on the x‐axis are included. This leads to 384/2=192 settings included in the aggregation in each column (except for the number of thresholds, there 384/3=128 settings are included). Each row comprises a head‐to‐head model comparison between two models. A value of 60% for the comparison X vs. Y in the column continuous outcome means that model X outperforms model Y in 60% of the simulation settings with a continuous outcome. The rows are ordered based on the average values in the block of columns associated with the bias. AUC, area under curve.

A more detailed comparison of model performance is shown in Figure 4, where we visualize the individual results on model superiority in a heatmap, stratified by parameter dimension, bias, coverage, and convergence. From the heatmap, we can deduce that models performing better in terms of bias tend to outperform others only rarely in terms of coverage and convergence. However, within performance in bias, coverage, or convergence, variation in a head‐to‐head model comparison is only minor on average. This suggests that differences in performance between models are larger than differences between the simulation settings within a head‐to‐head model comparison. Notable exceptions to this pattern would be the cases, where one model is superior (inferior) to another in one variation of a parameter dimension but inferior (superior) in the other variation. This would point to a clear change of model superiority, depending on the characteristics of the meta‐analysis dataset. However, we do not observe such changes in any of the parameter dimensions (omitting the comparisons containing the logit LMM for the number of thresholds due to its inestimability for $T_{i}=1$).

## 4 Application

From the simulation alone, it is hard to conclude how large differences in estimates of different methods for the meta‐analysis of DTA studies can be in real‐data situations. In this section, we provide two case studies to illustrate practical implications of using different STM and MTM for meta‐analysis of DTA studies by estimating all 11 included models on real‐world data.

### 4.1 Continuous Outcome—HbA1c to Detect Type 2 Diabetes

**FIGURE 5:**
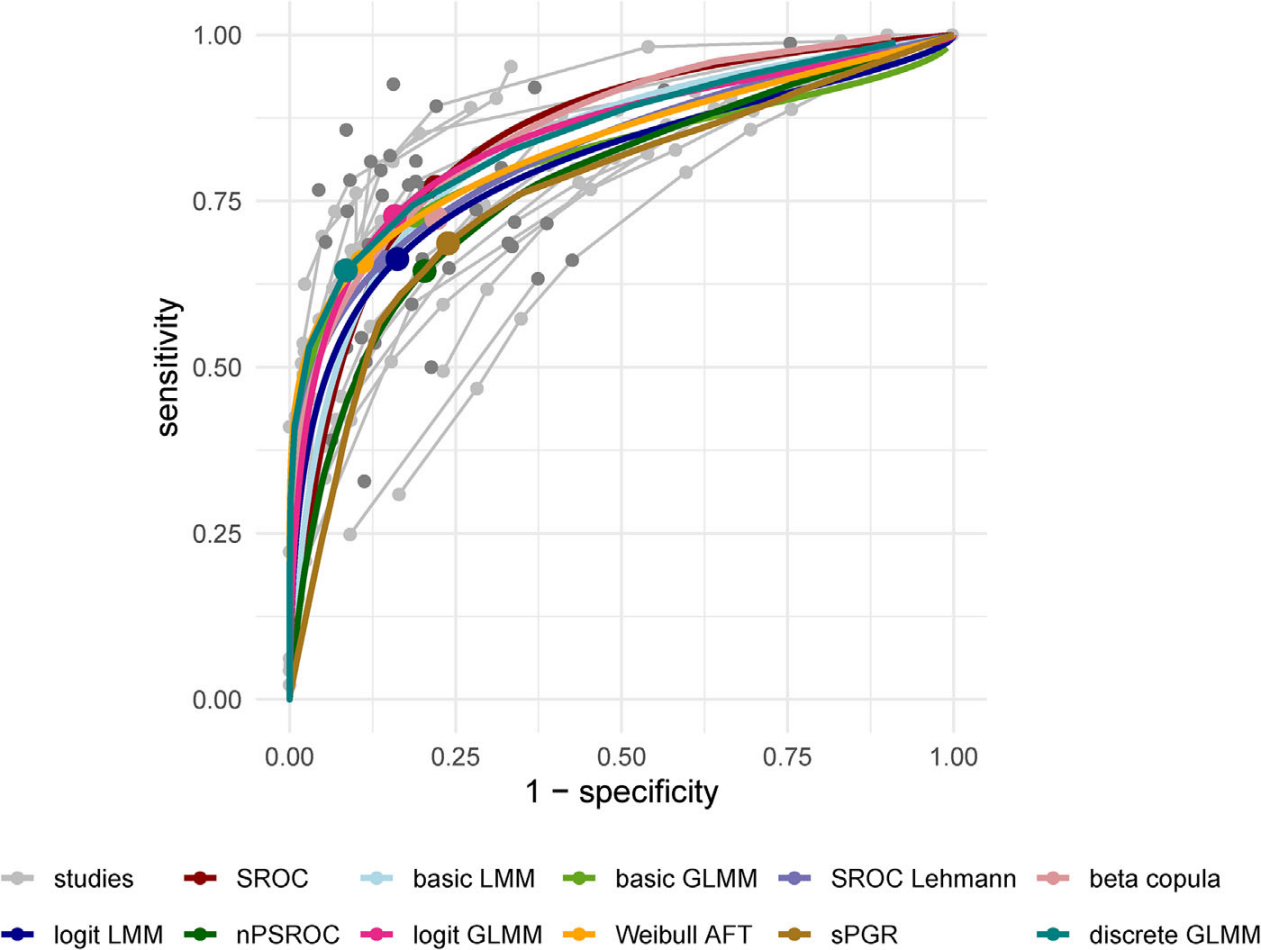
Estimated SROC curves of all 11 included models on ${\mathrm{HbA}}_{1c}$ dataset. The estimated pairs of optimal 1‐specificity and sensitivity are highlighted. Originally published study‐specific results (dark grey) and full study‐specific results (light grey, each line corresponds to a single study) are shown for comparison. SROC, summary receiver operating characteristic.

The continuous biomarker ${\mathrm{HbA}}_{1c}$, measured in percentage points, is used in medical practice to diagnose type 2 diabetes (Hoyer et al. 2018). We re‐analyze a meta‐analysis dataset on this biomarker, based on two systematic reviews (Bennett et al. 2007; Kodama et al. 2013) in the version first published in Hoyer et al. (2018) that contains results from 38 primary studies (median number of individuals per study: 1052, Q1 to Q3: 386 to 3565). The median type 2 diabetes prevalence, as measured by the reference standard oral glucose tolerance test, is 13% (Q1 to Q3: 5% to 21%). For each study, the originally included diagnostic thresholds are known (a single threshold per study), along with extended results with a total of 124 thresholds (median number in each study: 2, Q1 to Q3: 1 to 4.75). All studies combined report on 26 unique thresholds, in the range between 3.9% and 7.6%. An overview of the study‐specific sensitivities and specificities is given in Figure 5.

**FIGURE 6:**
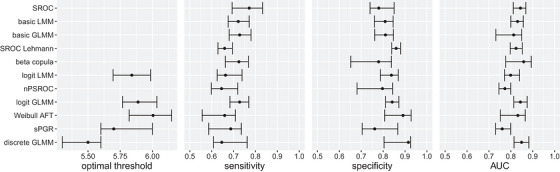
Estimated optimal diagnostic thresholds, sensitivities, specificities, and AUC for all included models on ${\mathrm{HbA}}_{1c}$ dataset, with 95%‐CIs, based on a non‐parametric bootstrap with 1000 replicates. All estimates are computed using the maximum unweighted Youden‐index. AUC, area under curve; CI, confidence interval.

We estimate all 11 models on the ${\mathrm{HbA}}_{1c}$ dataset, either in its full version for the MTM or in the version with originally published results for the STM. Both versions of the dataset are published in Hoyer et al. (2018) and also available from https://gitlab.ub.uni‐bielefeld.de/stoyef/metaROC/‐/tree/simstudy. Figure 5 shows estimated SROC curves, along with highlighted optimal pairs of sensitivity and specificity for all models. Additionally, Figure 6 shows estimated optimal sensitivities, specificities, AUC, and optimal threshold if applicable, along with 95%‐CIs from a non‐parametric bootstrap of the primary studies. We observe substantial deviations in estimated AUC (range: 0.761–0.859), optimal sensitivity (range: 0.645–0.771), specificity (range: 0.761–0.915), and diagnostic threshold (range: 5.5–6.01). See Table S7 for all numerical values.

If we consider the simulation setting that resembles the data distribution most closely out of all 384 settings presented in Section 3, we can adjust the model estimates by their estimated biases from the simulation (see the left part of Figure S15). This shrinks the range of adjusted AUC to [0.802;0.844]. Similar adjustments can be made for optimal sensitivity, specificity, and diagnostic threshold.

When screening for type 2 diabetes, it may be important to achieve high specificities of diagnostic tests, as the financial impact of treating false positives may be high. In case of MTM, we can increase the weight of specificity when computing the optimal threshold based on the weighted Youden‐index. Using a weight of 80% for the specificity leads to an increase of the estimated optimal threshold in the range [5.6;6.4], see Table S6. Corresponding results for an increase in sensitivity weight are shown in Table S8.

### 4.2 Ordinal Outcome—HADS‐A to Screen for Any Anxiety Disorder

The Hospital Anxiety and Depression Scale Anxiety subscale (HADS‐A), a subscale of the Hospital Anxiety and Depression Scale (HADS), is used to screen for anxiety disorders (Zigmond and Snaith 1983). Patients can score between zero and three points on seven items, for a total score between 0 and 21. In a recent Cochrane review, Fomenko et al. (2025) investigated the accuracy of HADS‐A to screen for any anxiety disorder in a meta‐analysis. We use a modified version of their data, consisting of 55 primary studies (median number of individuals per study: 132, Q1 to Q3: 95.5 to 246.5) (Fomenko et al. 2025). The median prevalence of any anxiety disorder in the studies is 16.8% (Q1 to Q3: 9.8% to 25.2%), determined by the reference standard of validated structured or semi‐structured clinical interviews (Fomenko et al. 2025). In the full dataset, studies report results on a median of 13 thresholds (Q1 to Q3: 2 to 19), leading to 609 results reported in total. We estimate the six MTM on the full dataset and the five STM on a subset containing results corresponding to the closest reported threshold to 10 per study. There are different established thresholds used for the HADS‐A questionnaire, e.g., 8 for doubtful cases and 11 for definite cases (Zigmond and Snaith 1983; Fomenko et al. 2025). We choose the threshold 10 as a compromise.

**FIGURE 7:**
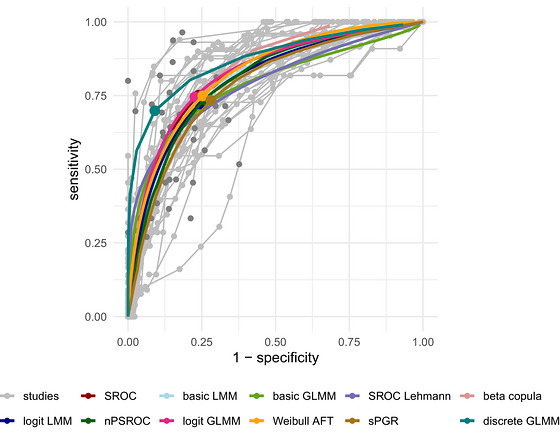
Estimated SROC curves of all 11 included models on HADS‐A dataset. The estimated pairs of optimal 1‐specificity and sensitivity are highlighted. The thresholds nearest to 10 from the originally published study‐specific results (dark grey) and full study‐specific results (light grey, each line corresponds to a single study) are shown for comparison. HADS, Hospital anxiety and depression scale; SROC, summary receiver operating characteristic.

**FIGURE 8:**
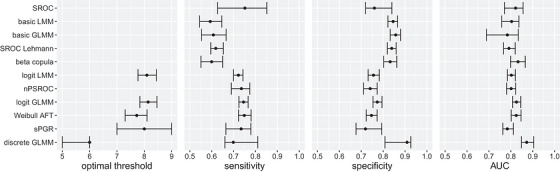
Estimated optimal diagnostic thresholds, sensitivities, specificities, and AUC for all included models on HADS‐A dataset, with 95%‐CIs, based on a non‐parametric bootstrap with 1000 replicates. All estimates are computed using the maximum unweighted Youden‐index. AUC, area under curve; CI, confidence interval; HADS, Hospital anxiety and depression scale.

The estimated performance of HADS‐A is illustrated by SROC curves in Figure 7. Notably, summary estimates of STM differ from estimates based on the optimal Youden‐index. Figure 8 additionally shows forest plots of estimated AUC, along with optimal sensitivity, specificity, and (if applicable) diagnostic threshold with respect to the unweighted maximum Youden‐index. AUC estimates range from 0.784 to 0.873, sensitivity estimates from 0.593 to 0.751, specificity estimates from 0.719 to 0.909, and threshold estimates from 6 to 8 (rounded to integers, see Table S10). The corresponding 95%‐bootstrap CIs are slightly narrower than those in Section 4.1 (median width for AUC CI: 0.053 vs. 0.068), likely due to the higher number of studies included (55 vs. 38). The included models vary in their estimation in a similar magnitude to the ${\mathrm{HbA}}_{1c}$ example, except for the discrete GLMM, which estimates a substantially higher AUC than the other models and the basic LMM, basic GLMM, SROC Lehmann and beta copula models, which estimate lower sensitivity and higher specificity than most other models. This behavior concurs with our observations in the simulation (see Section 3.2), which suggested a systematic overestimation in AUC of the discrete GLMM in the case of ordinal outcomes.

As suggested in Section 4.1, we can adjust the point estimates by the bias of the closest simulation setting (right part of Figure S15). Doing this, the range in estimated AUC shrinks to [0.776;0.834]. Most notably, subtracting the bias from the estimated AUC of the discrete GLMM (0.873−0.084=0.789) substantially diminishes the deviation in estimated AUC of this model from the others (see Figures 7 and 8), for which the estimated biases are much smaller (see top‐right of Figure S15).

In screening settings of anxiety disorders, high sensitivities of diagnostic tests may be of importance. If we weigh sensitivity with 80% in the computation of the Youden‐index, the optimal diagnostic threshold is estimated to be between 3 and 5, resulting in higher sensitivity and lower specificity (rounded to integers, see Table S11). However, if medical personnel capacity is limited, practitioners may instead favor a higher specificity, reducing the number of false positives. Employing a weight of 80% on the specificity instead, leads to an estimated optimal diagnostic threshold between 7 and 14 (see Table S9).

## 5 Discussion

In this paper, we presented results of a comprehensive simulation study that compared the performances of 11 models for the meta‐analysis of DTA studies. We explicitly compared simulation settings with and without multiple diagnostic thresholds per study, as well as methods that can or cannot consider multiple thresholds per study. Based on the results presented in Section 3, it becomes clear that no model fits all situations. However, combining the different approaches to model comparison, we find the SROC Lehmann (bias in optimal sensitivity and specificity), beta copula (bias in optimal sensitivity), nPSROC (bias in AUC), sPGR (bias in AUC), and discrete GLMM (bias in optimal specificity) models to be systematically biased in our selected simulation settings. The three remaining STM (SROC, basic LMM, basic GLMM) and MTM (logit LMM, logit GLMM, Weibull AFT) all behave comparably well, but can be differentiated by the inferences allowed from their estimates. For the SROC model, only empirical coverage of sensitivity can be assessed, as it is a univariate model for the sensitivity. Further, all STM possess a structural disadvantage because they do not allow inference on the optimal diagnostic threshold. Without being inferior in settings where each study only reports on a single threshold, the MTM are thus able to give more informative results than the STM. Additionally, the logit GLMM is superior to the basic GLMM regarding empirical coverage (see Section 3.6). When comparing only the logit LMM, logit GLMM, and Weibull AFT model to each other, the logit GLMM appears to be the most robust choice. This is due to the inferior convergence behavior of the Weibull AFT model, the inability of the logit LMM (in its implementation in diagmeta) to estimate effect measures from data where only a single diagnostic threshold per study is included, and the higher number of settings where performance of the logit GLMM was satisfying for all performance measures (see Section 3.2). This being said, the substantial variation in model performance depending on the simulation setting, especially regarding the outcome type (see Section 3.4), makes it vital to investigate theoretical model performance based on suitable simulations as a basis for selecting the best model in a particular application. We point to the interactive Figures I1– I11 that allow fine‐grained comparison of all 11 models regarding all performance measures in all included simulation settings. Additionally, computation of bootstrap confidence intervals and sensitivity analyses using additional models may be helpful to assess potential variation in the estimates.

In Section 4, we applied all 11 models on 38 studies investigating the diagnostic accuracy of the continuous biomarker ${\mathrm{HbA}}_{1c}$ for type 2 diabetes and on 55 studies investigating the ordinal questionnaire HADS‐A to screen for any anxiety disorder. While the included models vary substantially in their estimates, correcting for their estimated bias in the simulation setting that resembles the applications most closely reduces this variation. This behavior indicates that performing simulations that resemble real‐data situations can add value to meta‐analysis of DTA studies.

A key result of our simulation is the notable lack of a systematic dependence of model performance on the number of diagnostic thresholds reported on by the individual studies (see Section 3.5). This observation points to the usability of STM in multiple threshold situations and vice versa for the MTM. A possible explanation for the observed model robustness may be attributed to our choice of data‐generating mechanisms. We only considered benign simulation settings without the presence of potential publication bias, a known issue in the area of meta‐analysis (van Enst et al. 2014). While such considerations are out of the scope of this paper, further investigations could be a meaningful research avenue to quantify the impact of publication bias on STM and MTM model performance.

With the presented results in mind, we postulate that practitioners should primarily base their model choice on the quantities of interest, when conducting a meta‐analysis of DTA studies. If interest is purely on overall test accuracy (AUC), STM such as the basic GLMM may safely be considered, even in the presence of multiple diagnostic thresholds per study. If the inference on the optimal diagnostic threshold is of interest, MTM such as the logit GLMM or Weibull AFT model may be a suitable choice. Using an MTM bears additional advantages that were out of the scope of our simulation, but pointed out in the two case studies in Section 4: by applying different weights on sensitivity and specificity in a weighted Youden‐index, different sets of optimal sensitivity and specificity, along with a corresponding optimal threshold, can be generated based on a single model estimation. Combined with the possibility of including additional covariates in the MTM, this opens up possibilities in the field of precision diagnostics (Borrebaeck 2017), allowing inference on an optimal diagnostic threshold specific to both external (e.g., monetary) conditions of screening settings and the individual patient characteristics. All compared models are applicable to cases where only very few studies are available to synthesize. However, estimation uncertainty may increase for fewer studies (see Figure S16 for results on an additional simulation setting with between two and three studies per meta‐analysis).

This study possesses several strengths. To our knowledge, it is the first phase III simulation study (Heinze et al. 2024) that jointly compares STM and MTM to each other, without laying the focus on a specific method. Additionally, it investigates a broad range of realistic simulation settings, considering both continuous and ordinal outcomes, as well as single‐threshold and multiple‐threshold situations. Instead of generating the simulation data from one of the included models, all included models do not align with the data‐generating processes, enabling fair model comparison. Pre‐registration of this study in the Open Science Framework (Stoye et al. 2025) additionally ensures a transparent and multidimensional evaluation process.

Nevertheless, this study also possesses some weaknesses. By restricting to 11 out of the 27 identified models in the literature for the meta‐analysis of DTA studies, there exists the possibility that we excluded one or several models that are superior to all models included. Our decision of inclusion and exclusion of every model (documented in Supporting information C) was made in a combination of pragmatism to keep the scale of the simulation study manageable, and the availability of model implementations. While it would be of interest to compare the included models to Bayesian models for the meta‐analysis of DTA studies (Rutter and Gatsonis 2001; Jones et al. 2019), this was computationally not possible for a simulation study of our scale. We excluded the generalized F family model (Hoyer and Kuss 2020) because it is used as the data‐generating process of the continuous outcome settings. Because it is of similar structure to the Weibull AFT model (but more general) and did not lead to a large jump in performance when compared to each other in the simulation of Hoyer and Kuss (2020), we do not expect it to substantially outperform the Weibull AFT model in our simulation. Nevertheless, additional simulation efforts could include the generalized F model as a comparator. Model performance in simulation studies is mainly driven by the design of data‐generating mechanisms. It is possible that assuming a study‐specific bivariate Gaussian random effect on the test value distribution for continuous outcomes favors certain models that also include a bivariate random effect in their model structure. However, we do not observe structural advantages of these models in the continuous outcome case, when compared to ordinal outcomes, where no Gaussian random effect was used during data generation. We also are not aware that any of the included models have a structural advantage over others, regarding the Generalized F and Dirichlet multinomial test value distributions used for continuous, respectively ordinal outcomes. To completely avoid parametric assumptions to the data‐generating mechanisms, future research efforts could be made to investigate the potential of plasmode simulations in the area of meta‐analysis of DTA studies (Schreck et al. 2024). In our selection of performance measures (see Figure S5 for additional results on the root mean squared error that are consistent with the reported results regarding bias), we were restricted by the necessity to rely on the optimal Youden‐index to get SROC curves for the STM and optimal sensitivity, specificity, and threshold estimates for the MTM. If all included models were able to make inferences on the diagnostic threshold, we could instead estimate summary sensitivity and specificity at fixed thresholds as often done in the literature (Hoyer et al. 2018; Hoyer and Kuss 2020; Zapf et al. 2024). Alternatively, we could have varied the computation of optimal summary sensitivity and specificity using a weighted Youden‐index in case of the MTM. We did not want to increase the complexity of our simulation setup any further but recognize that additional investigations may prove helpful, as sensitivity and specificity are often of different priority in clinical practice. Note, however, that using the unweighted Youden‐index can be considered a conservative choice regarding performance of logit (G)LMM, because medium values for estimated sensitivity and specificity are impacted stronger by bias in model parameters than values closer to the edge of their domain [0;1], due to the symmetry in the logit‐transformation. Additionally, computing Wald‐type CIs to assess empirical coverage may not be appropriate for all models (Stoye et al. 2024). Using non‐parametric bootstrap CIs instead may be preferable, but was computationally infeasible in our simulation study. Our assumption of sampling diagnostic thresholds with equal probability over the whole threshold domain (apart from setting an additional point weight to a threshold) may be too simplistic. We could instead sample the thresholds from different symmetric or skewed distributions, with the recommended threshold as expected value. While this is an important aspect to systematically investigate potential effects of publication or selection bias, we deemed it to be out of the scope of our simulation study. Further, we implicitly assume in our simulation that it is actually possible for a DTA study to report results on multiple diagnostic thresholds. This assumption does not hold true for all diagnostic tests, some of which may only be available in dichotomous form (e.g., the presence of some symptom in a patient may be used as a diagnostic test for a target condition). Our simulation results are not generalizable to these cases, where the diagnostic test does not possess any quantifiable threshold. Finally, we cannot exclude the possibility of inventor bias (Heinze et al. 2024) as we are developers of some of the included models. To ensure full transparency, we point to the pre‐registration of this study (Stoye et al. 2025) as well as the code repository that includes source code to all simulation experiments, results, figures, and tables, available at https://gitlab.ub.uni‐bielefeld.de/stoyef/metaROC/‐/tree/simstudy.

This paper provides in‐depth insight into the performance of 11 methods for the meta‐analysis of DTA studies (five of which consider a single threshold, six of which consider multiple thresholds per study). Using a broad variation of underlying simulation parameters in eight parameter dimensions, we conclude that no single model is preferable over all others. Out of the 11 models, the logit GLMM (and with restrictions the logit LMM and Weibull AFT model) seems best suited in most cases. Nevertheless, practitioners should ideally investigate in a simulation study which model is most suitable for a particular use case, instead of simply applying a single model. Alternatively, they can state their choice in model(s) before conducting the analysis—e.g., in a statistical analysis plan—to limit researchers' degrees of freedom. To enable researchers to perform their own simulation studies as basis for an informed model choice, future research efforts should focus on creating low‐threshold tools that can be safely used without expertise in statistical software.

## Conflicts of Interest

F.V.S. developed the discrete GLMM. O.K. developed the BBM copula model, the nPMA model, the Weibull AFT model, the logit GLMM, the piecewise constant model, the generalized F family model, and the discrete GLMM. A.Ho. developed the BBM copula model, the nPMA model, the Weibull AFT model, the logit GLMM, the piecewise constant model, the generalized F family model, and the discrete GLMM.

## Funding

This research was supported by the Deutsche Forschungsgemeinschaft (DFG, German Research Foundation, Project number: 519901253) and the de.NBI Cloud within the German Network for Bioinformatics Infrastructure (de.NBI) and ELIXIR‐DE (Forschungszentrum Jülich and W‐de.NBI‐001, W‐de.NBI‐004, W‐de.NBI‐008, W‐de.NBI‐010, W‐de.NBI‐013, W‐de.NBI‐014, W‐de.NBI‐016, W‐de.NBI‐022).

## Open Research Badges

This article has earned an Open Data badge for making publicly available the digitally‐shareable data necessary to reproduce the reported results. The data is available in the Supporting Information section.

This article has earned an open data badge “**Reproducible Research**” for making publicly available the code necessary to reproduce the reported results. The results reported in this article could fully be reproduced.

## Supporting information


**Supporting File 1:** bimj70147‐sup‐0001‐SuppMat.pdf.

**Supporting File 2:** bimj70147‐sup‐0002‐Interactive_Figures.zip.

**Supporting File 3:** bimj70147‐sup‐0003‐simstudy_code.zip.

## Data Availability

We offer the R‐package metaROC which contains all methods used in this paper at https://gitlab.ub.uni‐bielefeld.de/stoyef/metaROC. Replication of the results and figures of this contribution is possible using the code supplied at https://gitlab.ub.uni‐bielefeld.de/stoyef/metaROC/‐/tree/simstudy. An archive of the simulation results presented in Section 3 is available from Zenodo (Stoye and Raths 2025a). The ${\mathrm{HbA}}_{1c}$ meta‐analysis data used in Section 4.1 is published in Hoyer et al. (2018) and also included as a dataset in the R‐package metaROC. The HADS‐A meta‐analysis data used in Section 4.2 is based on the data published in Fomenko et al. (2025), its modified version used in this manuscript is available from https://gitlab.ub.uni‐bielefeld.de/stoyef/metaROC/‐/tree/simstudy/simstudy_code/applications.
