# Supplementary material for: Comparison of Different Methods for the Meta‐Analysis of Diagnostic Test Accuracy Studies—A Simulation Study
Source: Biom J. 2026 Jul 2;68(4):e70147. doi: 10.1002/bimj.70147 (PMC13329219; doi:10.1002/bimj.70147)
Supplement: Supplementary file 1 — Supporting File 1: bimj70147‐sup‐0001‐SuppMat.pdf. [file BIMJ-68-e70147-s003.pdf]

SUPPLEMENTARY INFORMATION

Comparison of different methods for the meta-analysis of diagnostic test accuracy studies – a simulation study

Ferdinand V. Stoye, Olaf Raths, Alexander Hapfelmeier, Alexey Fomenko, Oliver Kuss and Annika Hoyer

A. Test-value distribution parameter choices in simulation data-generating processes

In the continuous outcome simulation settings, we draw true test values from the Generalized F distribution, $y \sim \mathcal{GF}(p, q, b, \lambda)$. See Hoyer and Kuss [1] for more details on the parameterization. Table S1 shows the concrete parameter choices and resulting true values of AUC, sensitivity, specificity, and threshold that depend on the population and the underlying diagnostic test quality. Figure S1 visualizes the corresponding probability density functions (pdfs).

Table S1. *Generalized F family parameter choices and resulting true AUC, optimal sensitivity, specificity, and threshold in the simulation data-generating processes.*

true AUC[a]	true sensitivity[b]	true specificity[b]	true threshold[b]	population	p	q	b	λ
0.7500	0.672	0.756	5.929	non-diseased	0.4000	−0.6000	1.610	0.160
				diseased	1.7473	0.5027	1.915	0.166
0.9000	0.821	0.871	5.845	non-diseased	0.4000	−0.6000	1.496	0.160
				diseased	1.7473	0.5027	2.029	0.166

[a] Rounded to four decimals.
[b] Optimal values with repect to unweighted Youden-index, rounded to three decimals.

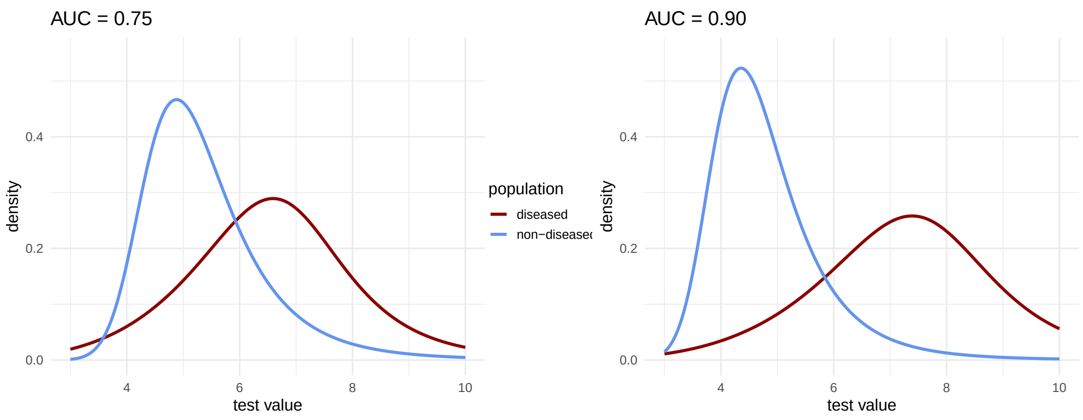

Figure S1. *Probability density functions of Generalized F distributions in the data-generating processes.*

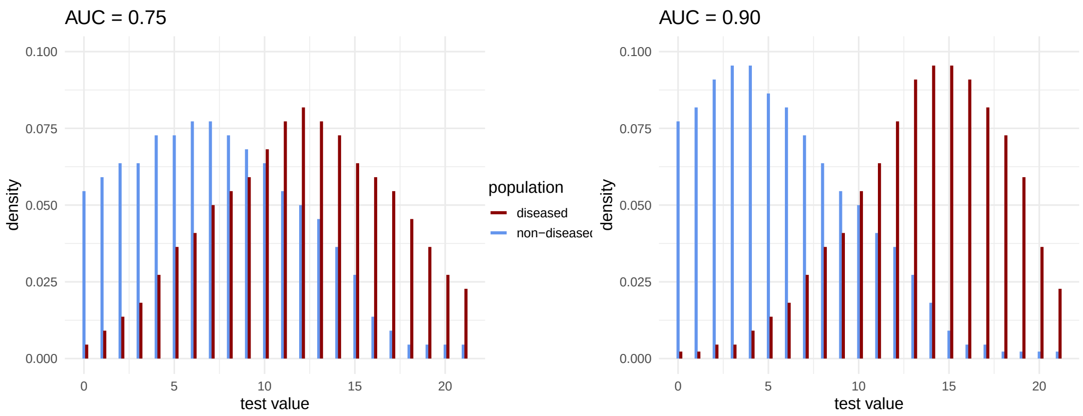

Figure S2. *Probability mass functions of multinomial distributions in the data-generating processes.*

In the ordinal outcome simulation settings, we draw true test values from the Dirichlet multinomial distribution (using the function `rdirmn` in the R-package `MGLM` [2]). Figure S2 shows the probability mass functions (pmfs) of the underlying multinomial distributions, again depending on the diagnostic test quality and the population. Table S2 shows the corresponding true values of AUC, sensitivity, specificity and threshold.

Table S2. *True AUC, sensitivity, specificity, and threshold for Dirichlet multinomial parameter choices in data-generating processes resulting from the pmfs shown in Figure S2.*

**true AUC**[a]	**true sensitivity**[b]	**true specificity**[b]	**true threshold**[c]
0.7500	0.686	0.682	10
0.9000	0.841	0.800	10

[a] Rounded to four decimals.
[b] Optimal values with repect to unweighted Youden-index, rounded to three decimals.
[c] Optimal values with repect to unweighted Youden-index.

For both distributions, we numerically compute the area under the ROC curve, which is based on the true sensitivities and specificities. We compute the true specificity as the cumulative distribution function (cdf) of the test value distribution of the non-diseased and the true sensitivity as 1−cdf of the test value distribution of the diseased.

B. Random effect parameter choices in simulation data-generating processes

Varying the heterogeneity across studies comes with different parameters for the random effects in the data-generating processes, both for the continuous and ordinal outcome. For continuous outcomes, we include a true bivariate Gaussian random effect in the data-generating process:

$$\begin{pmatrix} u_h \\ u_d \end{pmatrix} \sim \mathcal{N}\left(\begin{pmatrix} 0 \\ 0 \end{pmatrix}, \begin{pmatrix} \sigma_h^2 & \rho\sigma_h\sigma_d \\ \rho\sigma_h\sigma_d & \sigma_d^2 \end{pmatrix}\right).$$

In the low heterogeneity settings, we select similar true values for the variance-covariance matrix of the random effect to the estimates from the Weibull AFT model on the HbA1c dataset: $\sigma_h^2 = 0.005$,

$\sigma_d^2 = 0.01$, $\rho = 0.7$. For the high heterogeneity settings, we multiply the σ^2 by 2, while keeping ρ constant: $\sigma_h^2 = 0.01$, $\sigma_d^2 = 0.02$, $\rho = 0.7$.

As we sample the test values for the ordinal outcome settings from Dirichlet multinomial distributions, we can control the heterogeneity between studies using a factor by which the underlying probabilities of the multinomial distribution are multiplied. The larger this factor α (also known as concentration parameter), the less heterogeneity will be across studies studies. We fix $\alpha = 50$ in the low heterogeneity settings and $\alpha = 10$ in the high heterogeneity settings.

Algorithm S1 provides pseudocode on the full data-generating process in the simulation.

Algorithm S1 Pseudocode of data-generation in the simulation. Continuation in Algorithm S2.

for $s_r \in \{\mathcal{U}[2;15], \mathcal{U}[7;50]\}$ **do** ▷ Range of number of studies
 for $n_r \in \{\mathcal{U}[20;500], \mathcal{U}[100;3000]\}$ **do** ▷ Range in number of individuals
  for $T_{\max} \in \{1, 5, 21\}$ **do** ▷ Range in number of thresholds
   for outcome type ∈ {continuous, ordinal} **do** ▷ Outcome type
    for $p_r \in \{\mathcal{U}[0.01;0.1], \mathcal{U}[0.3;0.5]\}$ **do** ▷ Prevalence range
     for AUC ∈ {0.75, 0.9} **do** ▷ Test quality
      for $h \in$ {low, high} **do** ▷ Study heterogeneity
       for $w \in \{0, 0.7\}$ **do** ▷ Standard threshold weight
        for $r = 1, 2, \ldots, 1000$ **do** ▷ Simulation replicates
         Sample $I \sim s_r$; round I ▷ Number of studies
         for $i \in \{1, \ldots, I\}$ **do**
          Sample $N_i \sim n_r$; round N_i ▷ Number of individuals
          Sample $p_i \sim p_r$; $D_i = p_i \cdot N_i$; round D_i; $H_i = N_i - D_i$
          if outcome type == continuous **then** ▷ Random effects
           if heterogeneity == low **then**
            Sample $\begin{pmatrix} u_{i1} \\ u_{i2} \end{pmatrix} \sim \mathcal{N}\left(\begin{pmatrix} 0 \\ 0 \end{pmatrix}, \begin{pmatrix} 0.005 & 0.0049 \\ 0.0049 & 0.01 \end{pmatrix}\right)$
           else
            Sample $\begin{pmatrix} u_{i1} \\ u_{i2} \end{pmatrix} \sim \mathcal{N}\left(\begin{pmatrix} 0 \\ 0 \end{pmatrix}, \begin{pmatrix} 0.01 & 0.0099 \\ 0.0099 & 0.02 \end{pmatrix}\right)$
           end if
          else
           if heterogeneity == low **then**
            $\alpha = 50$
           else
            $\alpha = 10$
           end if
          end if
          for $j = 1, \ldots, H_i$ **do** ▷ Sample test values non-diseased
           if outcome type == continuous **then**
            if AUC == 0.75 **then**
             Sample $y_{ijh} \sim \mathcal{GF}(p = 0.4, q = -0.6, b = 1.61 + u_{i1}, \lambda = 0.16)$
            else
             Sample $y_{ijh} \sim \mathcal{GF}(p = 0.4, q = -0.6, b = 1.496 + u_{i1}, \lambda = 0.16)$
            end if
           else
            if AUC == 0.75 **then**
             Sample $y_{ijh} \sim \mathcal{DMN}(p_0 = 1.2/22, p_1 = 1.3/22, p_2 = 1.4/22, p_3 = 1.4/22, p_4 = 1.6/22, p_5 = 1.6/22, p_6 = 1.7/22, p_7 = 1.7/22, p_8 = 1.6/22, p_9 = 1.5/22, p_{10} = 1.4/22, p_{11} = 1.2/22, p_{12} = 1.1/22, p_{13} = 1/22, p_{14} = 0.8/22, p_{15} = 0.6/22, p_{16} = 0.3/22, p_{17} = 0.2/22, p_{18} = 0.1/22, p_{19} = 0.1/22, p_{20} = 0.1/22, p_{21} = 0.1/22, \alpha)$
            else
             Sample $y_{ijh} \sim \mathcal{DMN}(p_0 = 1.7/22, p_1 = 1.8/22, p_2 = 2/22, p_3 = 2.1/22, p_4 = 2.1/22, p_5 = 1.9/22, p_6 = 1.8/22, p_7 = 1.6/22, p_8 = 1.4/22, p_9 = 1.2/22, p_{10} = 1.1/22, p_{11} = 0.9/22, p_{12} = 0.8/22, p_{13} = 0.6/22, p_{14} = 0.4/22, p_{15} = 0.2/22, p_{16} = 0.1/22, p_{17} = 0.1/22, p_{18} = 0.05/22, p_{19} = 0.05/22, p_{20} = 0.05/22, p_{21} = 0.05/22, \alpha)$
            end if
           end if
          end for

C. Identified methods for meta-analysis of DTA studies

This section compiles all identified methods that perform meta-analysis of DTA studies, in chronological order of first publication. For each method, we include (1) a brief description and (2) a reason for exclusion in our simulation study, if applicable.

Throughout this section, we use the following abbreviations/definitions: LM – linear model; LMM – linear mixed model; GLMM – generalized linear mixed model; TP – true positives; FN – false negatives; FP – false positives; TN – true negatives; Se – sensitivity, Sp – specificity; SROC – summary receiver operating characteristic; logit(x) – $\log(x/(1-x))$; ϵ – model residuals; $i = 1, \ldots, I$ – study IDs in meta-analysis; D – number of diseased individuals in a study; H – number of non-diseased individuals in a study; AFT – accelerated failure time; MCMC – Markov chain monte carlo.

1. **basic LM** [3]
   - **Description:** Fixed effects model for Se, dependent on 1−Sp in logit-space. In detail, linear model with parameters α, β: $\text{logit}(\text{Se}) = \alpha + \beta \text{logit}(1-\text{Sp}) + \epsilon$.
   - **Reason for exclusion:** Similar to the SROC model; no concrete code for implementation (the authors propose profile likelihood estimation which is not as straightforward to implement as the SROC model).
2. **SROC** [4, 5]
   - **Description:** Fixed effects model for Se, dependent on 1−Sp in logit-space, with additional transformations. In detail, linear model with parameters α, β: $D = \alpha + \beta S + \epsilon$, where $D = \text{logit}(\text{Se}) - \text{logit}(1-\text{Sp})$ and $S = \text{logit}(\text{Se}) + \text{logit}(1-\text{Sp})$.
   - **Reason for exclusion:** N/A, included.
3. **StandDistance** [6]
   - **Description:** Instead of bivariate information, standardized distance between study populations is used as a single test quality measure, $d = \frac{\sqrt{3}}{\pi}\big(\log(\text{TP}) + \log(\text{TN}) - \log(\text{FP}) - \log(\text{FN})\big)$. Meta-analysis is conducted similar to standard meta-analysis of intervention studies, the standardized distances are interpreted as effect-sizes. Fixed and random effect models are proposed.
   - **Reason for exclusion:** Not possible to compare performance to models that use bivariate information.
4. **HSROC** [7, 8]
   - **Description:** Full Bayesian hierarchical model as an extension to the SROC model.
   - **Reason for exclusion:** Bayesian approach computationally infeasible for the simulation and equivalent to basic LMM in case of no covariates [9].
5. **metaROC 2-param.** [10]
   - **Description:** At first, the individual study ROC curves are fitted using 2-parametric models. Second, meta-analysis is carried out for the two parameters of the ROC curves, leading to an estimated SROC curve. For the meta-analysis two variants with random effects are used. Either two separate univariate models are fitted, one for each parameter or a model with bivariate random effect.
   - **Reason for exclusion:** No straightforward implementation and no code provided.
6. **unequal ordinal** [11]
   - **Description:** This model is an extension of the SROC model to multiple thresholds, with a possible extension to random effects using a Bayesian framework. The approach allows for an arbitrary number of ordered categories in each study which are then estimated in an ordinal regression structure.

- **Reason for exclusion:** No straightforward implementation, no code provided and Bayesian modeling approach is infeasible for the simulation.

7. **multiple ordinal** [12]

- **Description:** Using a normal approximation of underlying continuous test values, a fixed effects meta-analysis model is estimated that assumes the same ordinal thresholds in all studies.
- **Reason for exclusion:** All studies have to report on the same ordinal thresholds which makes the model not applicable in our simulation.

8. **basic LMM** [13–17]

- **Description:** LMM with bivariate random effect for logit(Se) and logit(Sp). In detail,

$$\begin{pmatrix}\text{logit}(\text{Se}_i)\\ \text{logit}(\text{Sp}_i)\end{pmatrix} \sim \mathcal{N}\left(\begin{pmatrix}\theta_{\text{Se}}\\ \theta_{\text{Sp}}\end{pmatrix}, \Sigma\right)$$

where Σ is the variance-covariance matrix of the bivariate normal distribution and includes the study weights as precisions by having

$$\Sigma = \Sigma' + C_i = \begin{pmatrix}\sigma^2_{\text{Se}} & \sigma_{\text{Se,Sp}}\\ \sigma_{\text{Se,Sp}} & \sigma^2_{\text{Sp}}\end{pmatrix} + \begin{pmatrix}s^2_{\text{Se},i} & 0\\ 0 & s^2_{\text{Sp},i}\end{pmatrix}$$

and $s^2_{\text{Se},i} = \frac{1}{D_i \cdot \text{Se}_i \cdot (1-\text{Se}_i)}$ and $s^2_{\text{Sp},i} = \frac{1}{H_i \cdot \text{Sp}_i \cdot (1-\text{Sp}_i)}$ are the variances of the estimated logit(Se) and logit(Sp). Different estimation schemes to maximum likelihood have been proposed, including double SIMEX [16], and hierarchical multinomial trees [17]. An extension to a finite mixture model, resulting in several estimated SROC curves for each mixture component, is also proposed [15].
- **Reason for exclusion:** N/A, included in the version of [13].

9. **basic GLMM** [14, 18, 19]

- **Description:** Instead of a LMM for logit(Se) and logit(Sp), a binomial GLMM is estimated (different link functions are possible, but we use the most commonly used logit-link). Otherwise, the model architecture is identical to the basic LMM.
- **Reason for exclusion:** N/A, included.

10. **multinomial** [20]

- **Description:** Relative $K \times K$ tables are modeled using a $K^2 - K$-dimensional normal distribution with a multinomial mixed model. The full model likelihood is the product of the K multinomial likelihoods, integrated over the $K^2 - K$-dimensional normal.
- **Reason for exclusion:** All studies have to report on the same K thresholds which makes the model not applicable in our simulation. Application of this model is (e.g.) cancer staging.

11. **MREM** [21, 22]

- **Description:** $2 \times (K+1)$ tables are assumed for each study. A multivariate hierarchical random effects model is fitted for logit(Se) which is assumed to be linearly dependent on logit(1−Sp). Within the studies, multinomial distributions are assumed. Additional to maximum likelihood estimation [21], estimation in a SIMEX framework has also been proposed [22].
- **Reason for exclusion:** All studies have to report on the same K thresholds which makes the model not applicable in our simulation. Application of this model is (e.g.) cancer staging.

12. **PCGF** [23]

- **Description:** The discrete hazard of the ordinal outcomes of the test are modeled. Survival methods are used to model the number of events in one ordinal category, using a Poisson distribution with the number of persons under risk as expectancy. The between-study heterogeneity is introduced by multivariate and positively correlated gamma-distributed random effects (one effect for diseased/non-diseased population and test outcome). The model is estimated in two stages using composite likelihood. First, conditional on the random effects, the marginals of the model can be interpreted as a negative binomial GLM with log-link. Second, the estimated hazard and frailty parameters are put into the log-likelihood of pairs to estimate the correlation parameters.
- **Reason for exclusion:** Only applicable to ordinal outcomes where every study reports on the same thresholds, which makes it inapplicable in our simulation study.

13. **SROC Lehmann** [24–26]

- **Description:** The Lehmann family $p = u^{\theta}$, with $u \in [0, 1]$ and $\theta > 0$ is proposed to model an SROC curve. Logarithmized, $\log(p) = \theta \log(u)$ and plugging in $p = \text{Se}$, $u = 1 - \text{Sp}$ and using normal approximations for $\log(p)$ and $\log(u)$ a model is estimated with adjusted profile likelihood maximization. An extension to a non-parametric mixture model, resulting in several estimated SROC curves for each mixture component, is also proposed [26].
- **Reason for exclusion:** N/A, included.

14. **SROC t_{α}** [27, 28]

- **Description:** Instead of logit-transformations, the t_{α} family of transformations is used, $t_{\alpha}(p) = \alpha \log(p) - (1 - \alpha) \log(1 - p)$, $p \in [0, 1]$, $\alpha \in [0, 2]$. This model is a generalization of the SROC Lehmann model and uses a compromise between a logit- and a log-transformation. An extension to a semiparametric mixture model (where estimation is proposed with the EM-Algorithm), resulting in several estimated SROC curves for each mixture component, is also proposed [28].
- **Reason for exclusion:** No rationale for estimation of α provided in the implementation in [29].

15. **BBM Sarmarnov** [30]

- **Description:** The number of observed TP and FP are modeled in one step using a bivariate beta-binomial distribution. The populations are linked through the Sarmanov family of distributions to allow correlation between the random effects.
- **Reason for exclusion:** Limited range of dependence in Sarmanov family [31], no straightforward implementation and no code provided.

16. **BBM copula** [32]

- **Description:** Instead of using Gaussian random effects, copulas with beta-binomial marginal distributions are proposed to model Se and Sp. Different copulas are possible. The resulting closed-form likelihood is numerically optimized.
- **Reason for exclusion:** Similar to normal/beta copula [31].

17. **logit LMM** [33–35]

- **Description:** Extension of basic LMM to the case of modeling multiple thresholds. The threshold is introduced as a covariate in the model. Different random effect structures and transformations of the threshold are possible, [34] introduce variants up to a four dimensional random effect (random intercept and slope for logit(1−Se) and logit(Sp)) but note that there may be convergence problems. Alternative model estimation using pseudo likelihood is also proposed [35].
- **Reason for exclusion:** N/A, included.

18. **nPMA** [36]

- **Description:** Non-parametric model for the meta-analysis of Se and Sp with a single diagnostic threshold per study. Fixed effects are assumed and Se (Sp) is estimated as the proportion of correctly identified diseased (non-diseased) individuals, pooled over all studies.
- **Reason for exclusion:** Only Se and Sp are estimated, not the AUC. This makes this model in the proposed form not applicable in our simulation study.

19. **normal/beta copula** [31]
    - **Description:** Similar to [32], but with different marginal distributions. Either Gaussian marginals for transformed Se and Sp or beta marginals for Se and Sp are proposed.
    - **Reason for exclusion:** N/A, included.
20. **nPSROC** [37]
    - **Description:** A weighted sum of the estimated individual ROC curves is proposed as the SROC curve. Both fixed and random effect approaches are considered in the calculation of individual study weights. Otherwise, the model is non-parametric.
    - **Reason for exclusion:** N/A, included.
21. **logit GLMM** [38]
    - **Description:** Extension of basic GLMM to the case of modeling multiple thresholds. The threshold is introduced as a covariate in the model. Different random effect structures and transformations of the threshold are possible. The authors propose a bivariate random intercept model.
    - **Reason for exclusion:** N/A, included.
22. **Weibull AFT** [39]
    - **Description:** Meta-analysis data can be re-arranged into interval-censored time-to-event data, where the time is the diagnostic test value and the events are the indicators if individuals have their test value in the intervals between subsequent thresholds. Different parametric assumptions can be made for the test values, e.g., Weibull. The model is estimated as an AFT model with bivariate random intercept u, leading to the linear term $\log(y_j) = \beta_j + u_j + \epsilon$, $j = d, h$ for test values y.
    - **Reason for exclusion:** N/A, included.
23. **Bayesian multinomial** [40]
    - **Description:** logit(1−Sp) and logit(Se) are modeled using a box-cox transformation of the threshold c, $(\mu_j - g(c))/\sigma_j$, $j = 1, 2$. μ_j, σ_j are modeled as random effects, different structures are possible here. Within each study, multinomial distributions are assumed for both populations that model the intervals between thresholds, with probabilities derived from the transformed 1−Sp and Se. The model is estimated in a Bayesian framework with MCMC.
    - **Reason for exclusion:** Bayesian model estimation is not feasible in our simulation framework.
24. **piecewise constant** [41]
    - **Description:** Instead of a parametric assumption for the interval-censored test values as in [39], a semiparametric structure is proposed that can be interpreted a piecewise-constant hazard modeling: $\lambda_j(t) = \lambda_{jh} \exp(\beta_d x_d + u)$ for unique increasing thresholds j, interval-specific constant baseline hazard λ_{jd}, indicator variable x_d for diseased individuals and univariate random effect u.
    - **Reason for exclusion:** Similar to [42] but without bivariate random effect.
25. **generalized F family** [1]
    - **Description:** Generalization of [39] to the four parametric generalized F distribution, otherwise with the same AFT model structure.
    - **Reason for exclusion:** Matches the data-generating process of the continuous outcome settings in the simulation.

26. **sPGR** [43]
    - **Description:** Pseudo observations are generated from uniform distributions between thresholds. Then, the summary AUC, Se and Sp are estimated non-parametrically using the global ranks of the pseudo data.
    - **Reason for exclusion:** N/A, included.
27. **discrete GLMM** [42]
    - **Description:** Using the time-to-event structure first proposed in [39], a binomial GLMM with bivariate random effect is estimated for the discrete hazard to be in each of the intervals between subsequent thresholds. Different link functions are possible, e.g., logit or complementary-log-log.
    - **Reason for exclusion:** N/A, included.

D. Delta method for standard errors of SROC model

Following Moses et al. [4], the model has two parameters that are estimated in a linear model, A and B. Back-transformation to the ROC scale yields a univariate curve, depending on the (fixed) FPR:

$$\text{TPR} = \text{logit}^{-1}\left(\frac{A + (B+1)\cdot \text{logit(FPR)}}{1-B}\right)$$

Standard errors for the TPR are then computed as:

$$\widehat{\text{SE}}(\text{TPR}) = \sqrt{\nabla_{\text{TPR}}^{\top}\hat{\Sigma}\nabla_{\text{TPR}}},$$

with estimated variance-covariance matrix Σ and

$$\nabla_{\text{TPR}} = \begin{pmatrix} -\dfrac{\exp\left(\frac{A}{1-B}\right)\left(\frac{\text{FPR}}{1-\text{FPR}}\right)^{\frac{B+1}{1-B}}}{(B-1)\left(\exp\left(\frac{A}{1-B}\right)\left(\frac{\text{FPR}}{1-\text{FPR}}\right)^{\frac{B+1}{1-B}}+1\right)^2} \\ \dfrac{\exp\left(\frac{A}{1-B}\right)\left(\frac{\text{FPR}}{1-\text{FPR}}\right)^{\frac{B+1}{1-B}}\left(A+2\log\left(\frac{\text{FPR}}{1-\text{FPR}}\right)\right)}{(1-B)^2\left(\exp\left(\frac{A}{1-B}\right)\left(\frac{\text{FPR}}{1-\text{FPR}}\right)^{\frac{B+1}{1-B}}+1\right)^2} \end{pmatrix}.$$

Plugging in the estimates of A and B, we can subsequently estimate the standard error of the TPR for any given FPR.

E. Additional simulation results

Figure S3 gives an overview of the entire simulation scenario space.

Table S4 and Table S5 shows summary statistics on the simulation results, pooled over all simulation settings with continuous, respectively ordinal, outcome.

Figure S4 stratifies the results into the different simulation settings in form of heatmaps, generated using the R-package `rsimsum` [44]. Figure S5 shows additional results on the RMSE for the different settings.

Figure S7 – Figure S12 visualize the effects of changing the parameter values in the data-generating mechanisms regarding bias in optimal sensitivity, specificity, and threshold, empirical coverage in sensitivity and specificity, and model convergence.

Figure S13 and Figure S14 show boxplots of estimated model performance stratified by splits into different settings. For each performance measure, the optimal splits are computed using Model-based Recursive Partitioning with the `partykit` R-package [45, 46]. In red, the fitted values from the models are drawn. The partitioning was stopped after the second split to identify the most important parameter

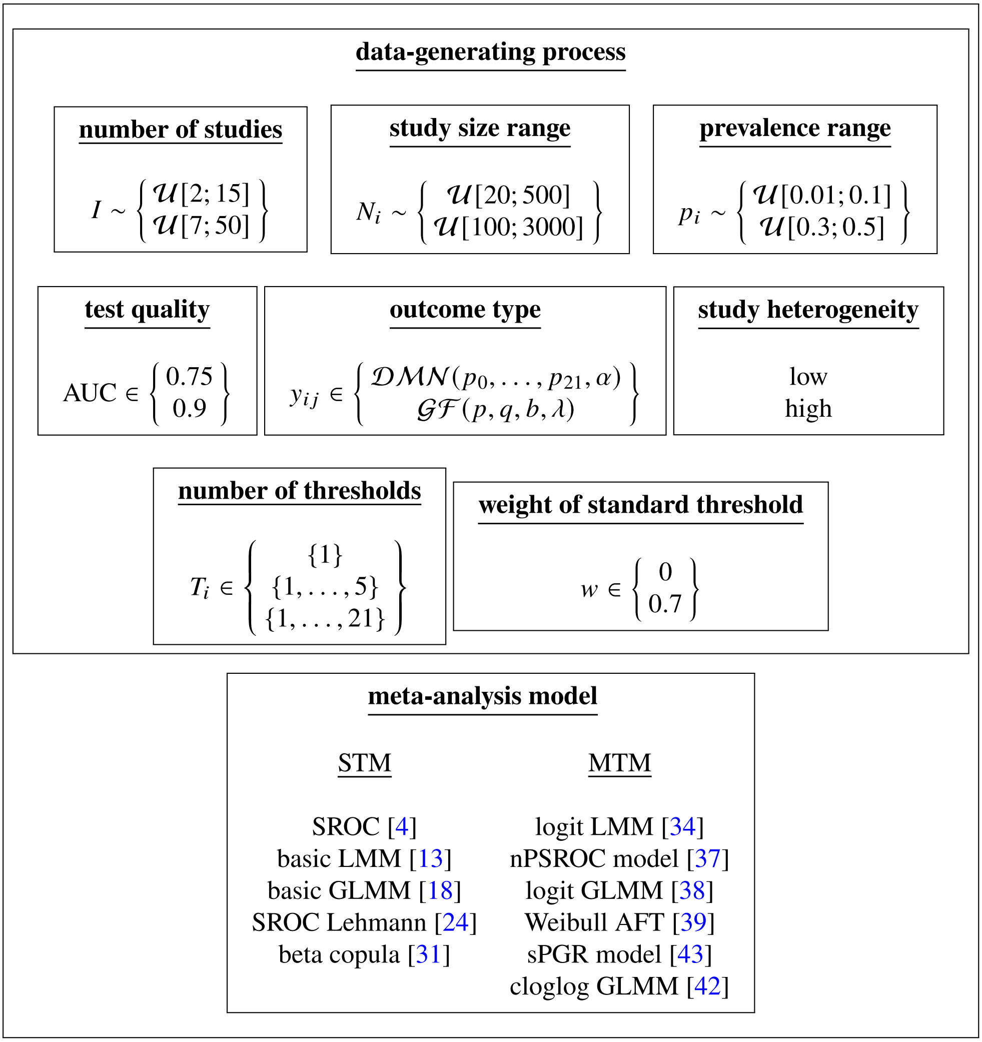

Figure S3. *Scenario space of the simulation study. N is the number of individuals in a study, and p is the prevalence of the disease which corresponds to the proportion of diseased individuals in a study. AUC is the area under the ROC curve of the diagnostic test. y is the test value of an individual. STM: single threshold method. MTM: multiple threshold method.*

dimensions during data generation for each performance measures. We do not show results on the convergence as the Model-based Recursive Partitioning did not find an optimal split here.

We exemplarily show detailed results for two selected settings in Figure S15 in form of lollipop plots [44, 47]. We selected the settings based on the highest similarity in their data-generating mechanisms to the datasets in the two case studies.

All included models are also applicable in cases where only very few studies are included in a meta-analysis. Figure S16 shows lollipop plots of the estimated performance of all models for an additional simulation setting where the number of studies per dataset was between two and three studies. While the estimated Monte Carlo CIs are wider than in Figure S15, there is no systematic deviation in estimated model performance from settings with larger number of studies.

Figure S17 and Figure S18 show boxplots of estimated model performance for STM, distinguishing between the two variations in weights for a standard diagnostic threshold during data generation, either

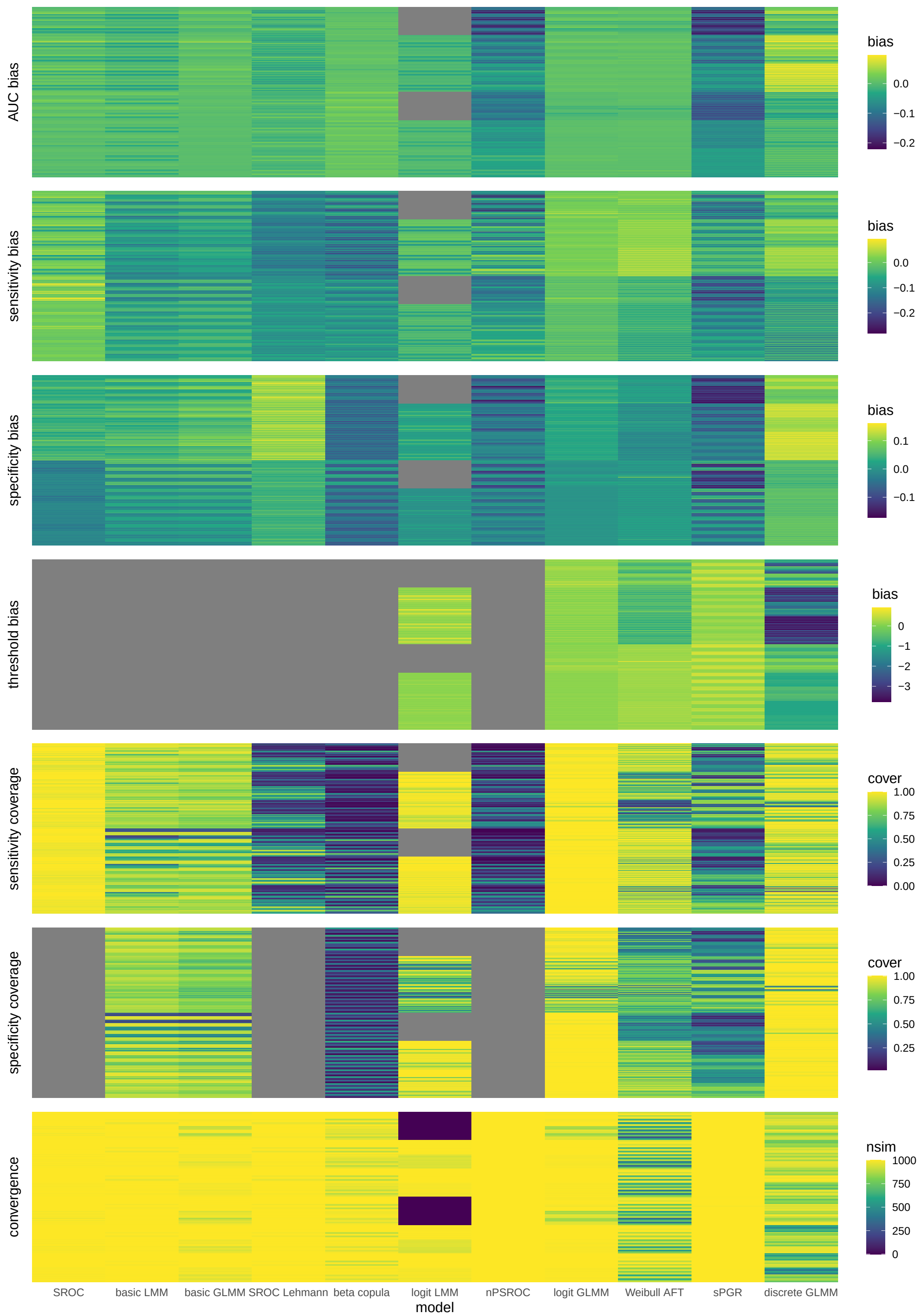

Figure S4. *Heatmap of all simulation results. Grey color indicates that estimating the measure was not possible for the model. The simulation settings are plotted vertically in each of the heatmap plots. Setting IDs are omitted to impove visibility.*

Table S3. *Pooled bias, coverage and convergence results (multiplied by 100, except for the threshold, and rounded to two decimals). Maximum Monte Carlo standard errors on the original scale are* 0.0002 *(bias in AUC, sensitivity, specificity),* 0.0027 *(bias in threshold),* 0.0008 *(coverage).*

	bias				coverage		
**model**	**AUC**	**sensitivity**	**specificity**	**threshold**	**sensitivity**	**specificity**	**convergence**
SROC [4]	0.57	0.65	1.54		98.34		99.58
basic LMM [13]	−0.51	− 7.03	3.27		81.55	82.20	99.65
basic GLMM [18]	0.90	− 5.56	4.49		82.27	76.85	98.09
SROC Lehmann [24]	−1.30	−10.09	7.49		45.66		99.67
beta copula [31]	1.43	−10.93	−4.68		22.15	23.53	96.65
logit LMM [34]	−1.16	− 3.25	1.46	0.18	96.04	85.60	98.22
nPSROC [37]	−7.46	− 8.54	−3.55		20.62		100.00
logit GLMM [38]	0.82	0.42	2.13	0.11	98.52	93.33	98.07
Weibull AFT [39]	0.78	− 0.83	1.39	−0.09	80.24	70.94	83.94
sPGR [43]	−9.19	− 9.53	−5.13	0.25	51.15	55.07	100.00
discrete GLMM [42]	1.33	− 2.83	9.10	−1.39	88.06	97.71	85.02

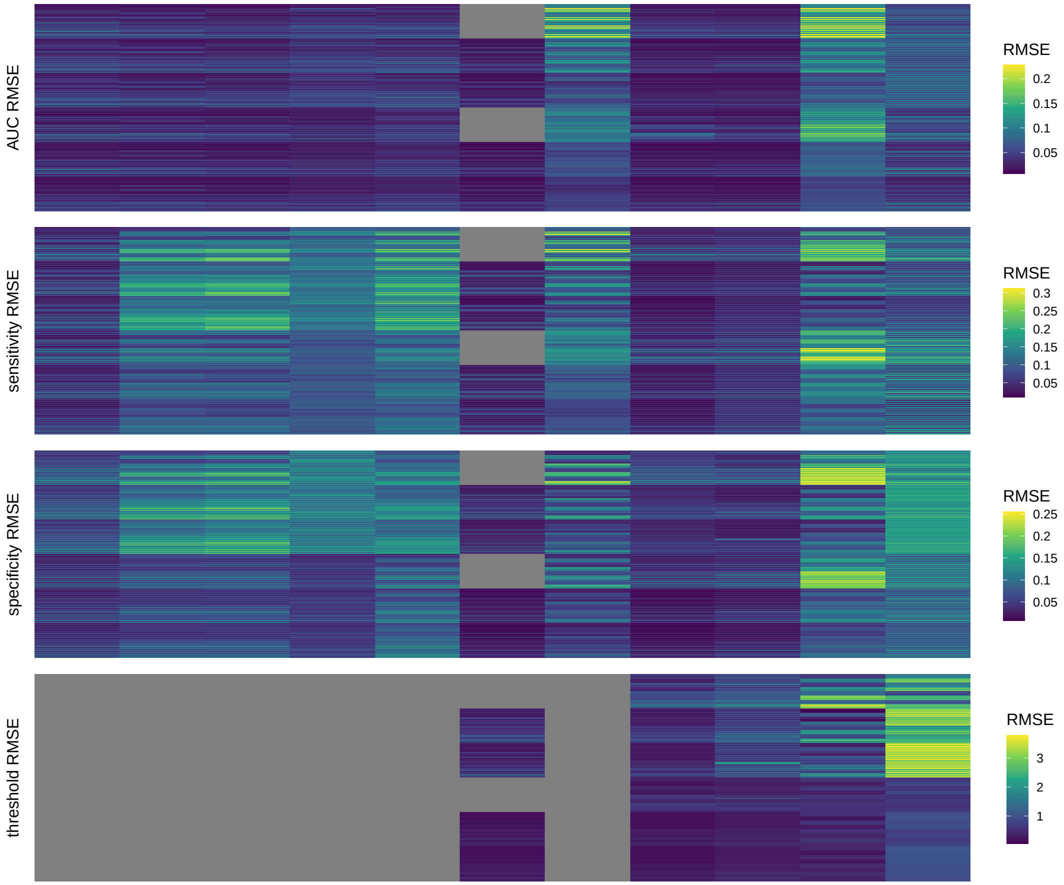

Figure S5. *Heatmap of all simulation results for root mean squared error (RMSE). Grey color indicates that estimating the measure was not possible for the model. The simulation settings are plotted vertically in each of the heatmap plots. Setting IDs are omitted to impove visibility.*

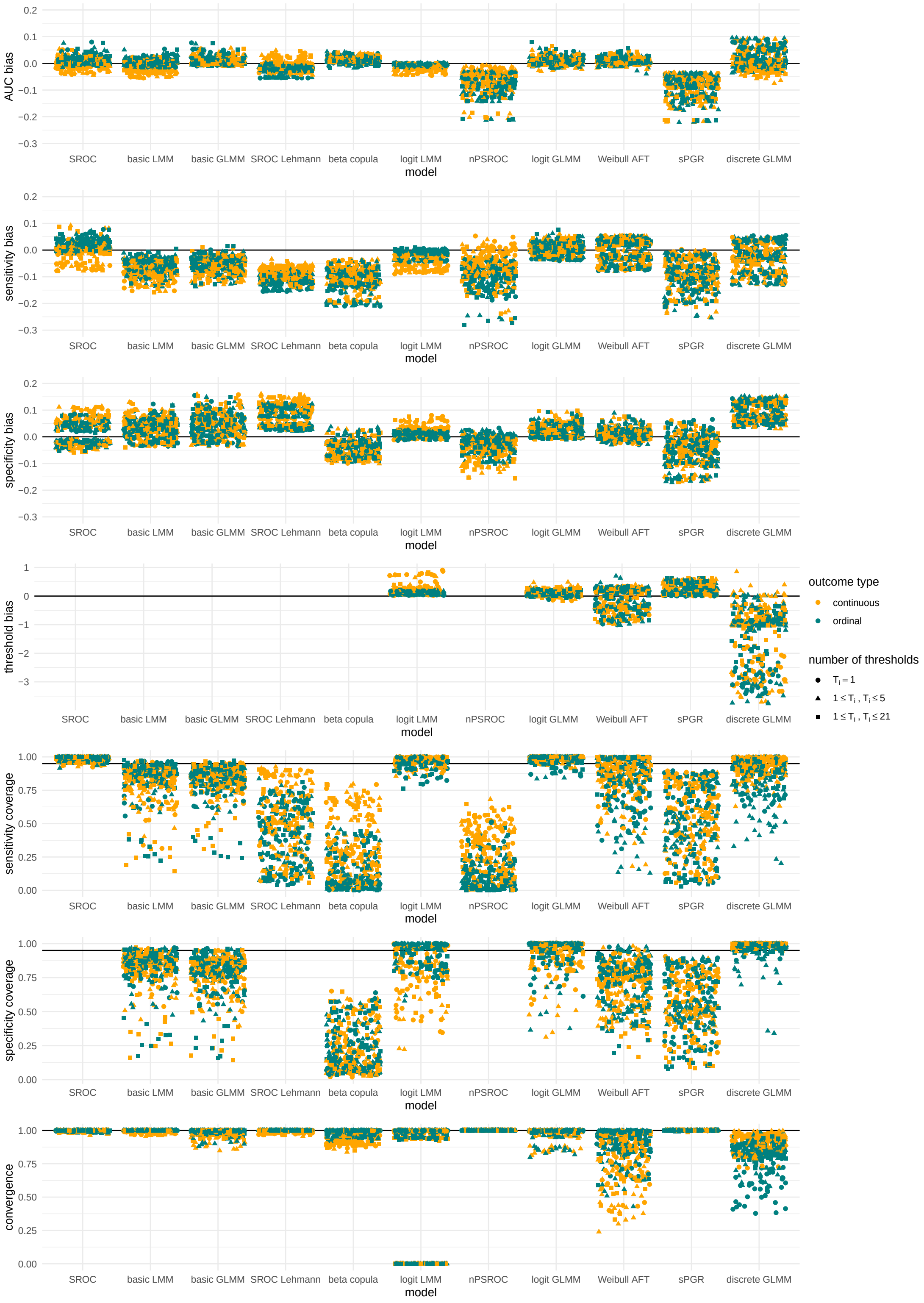

Figure S6. *Scatterplots of all simulation results. Each point corresponds to the measured performance of a model in a simulation setting. Points are jittered horizontally to improve visibility. Optimal values are indicated by horizontal lines.*

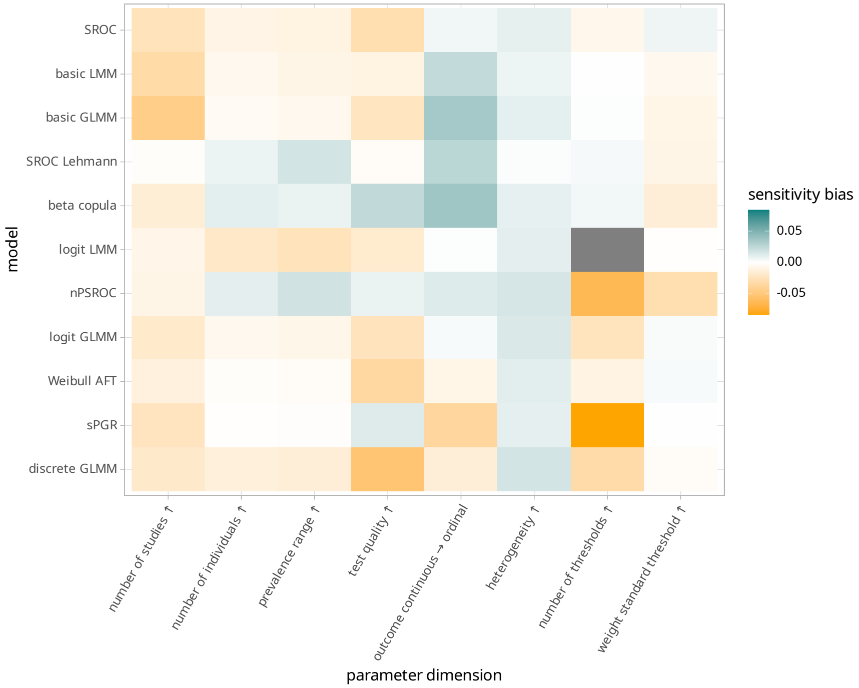

Figure S7. *Heatmap of mean change in absolute sensitivity bias when varying the parameters in the data-generating mechanism. The changes are denoted on the x-axis and displayed ceteris paribus. For the number of thresholds, the change from* $T_i = 1$ *to* $1 \leq T_i \leq 21$ *is visualized.*

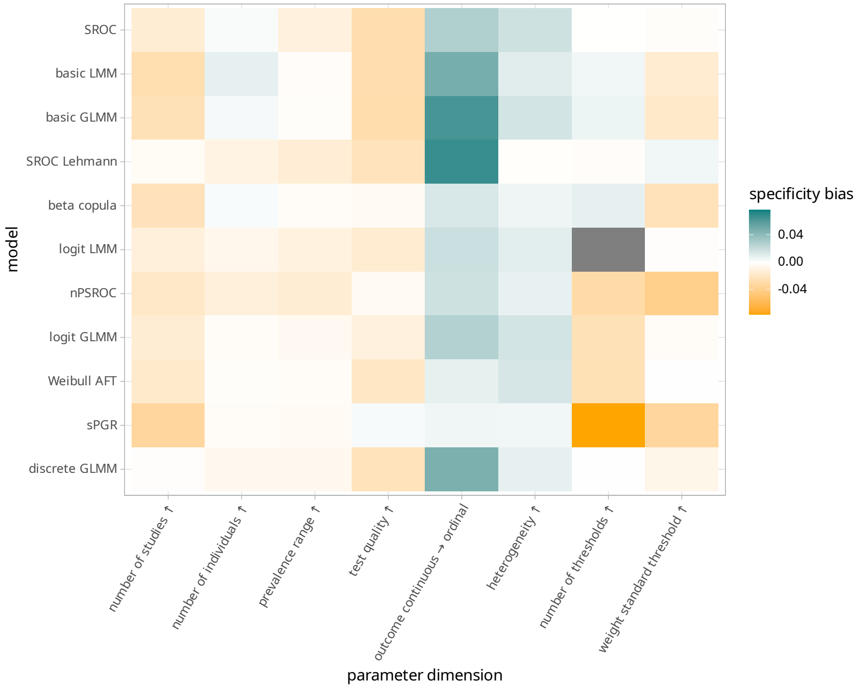

Figure S8. *Heatmap of mean change in absolute specificity bias when varying the parameters in the data-generating mechanism. The changes are denoted on the x-axis and displayed ceteris paribus. For the number of thresholds, the change from* $T_i = 1$ *to* $1 \leq T_i \leq 21$ *is visualized.*

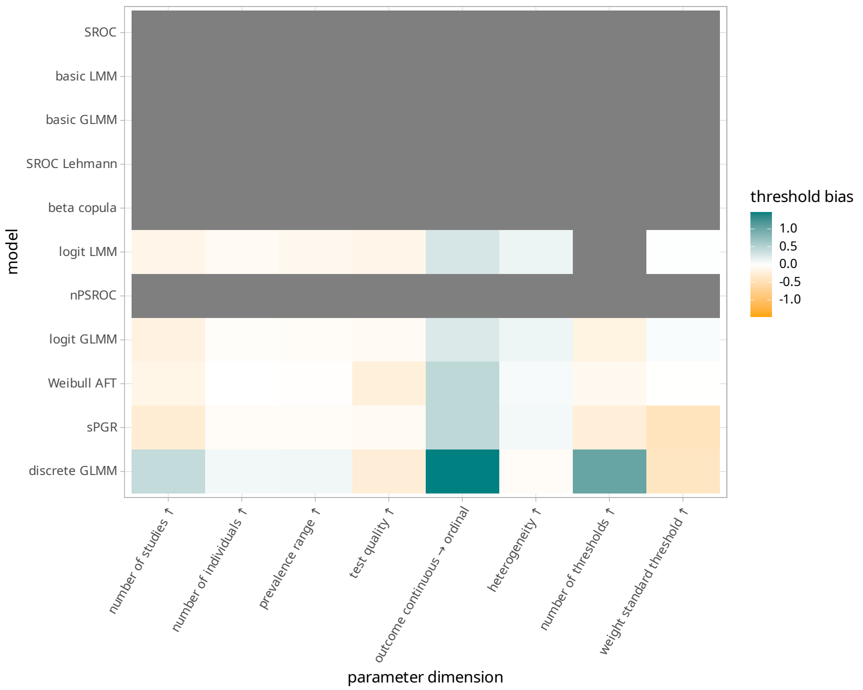

Figure S9. *Heatmap of mean change in absolute threshold bias when varying the parameters in the data-generating mechanism. The changes are denoted on the x-axis and displayed ceteris paribus. For the number of thresholds, the change from* $T_i = 1$ *to* $1 \leq T_i \leq 21$ *is visualized.*

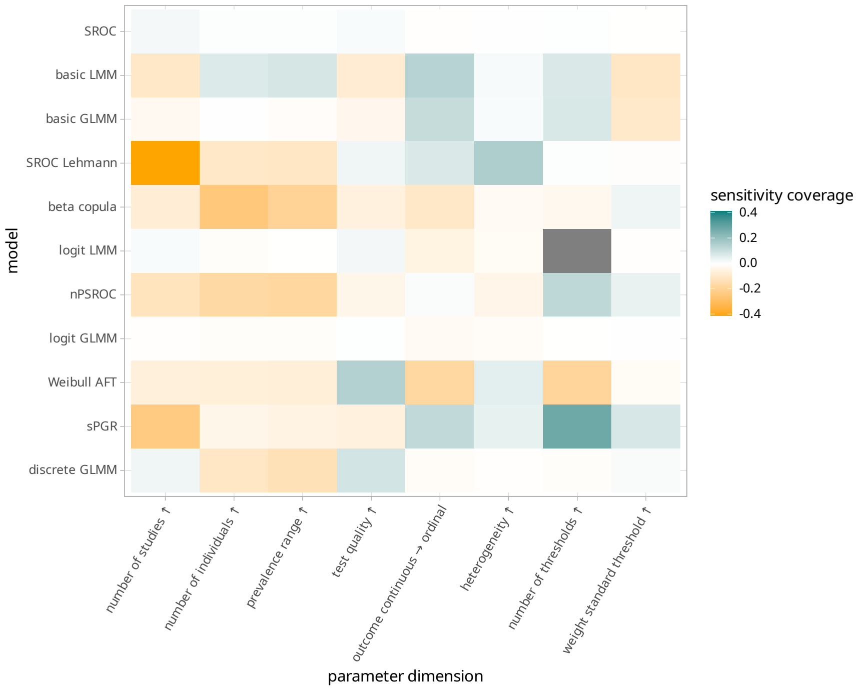

Figure S10. *Heatmap of mean change in empirical coverage of sensitivity when varying the parameters in the data-generating mechanism. The changes are denoted on the x-axis and displayed ceteris paribus. For the number of thresholds, the change from* $T_i = 1$ *to* $1 \leq T_i \leq 21$ *is visualized.*

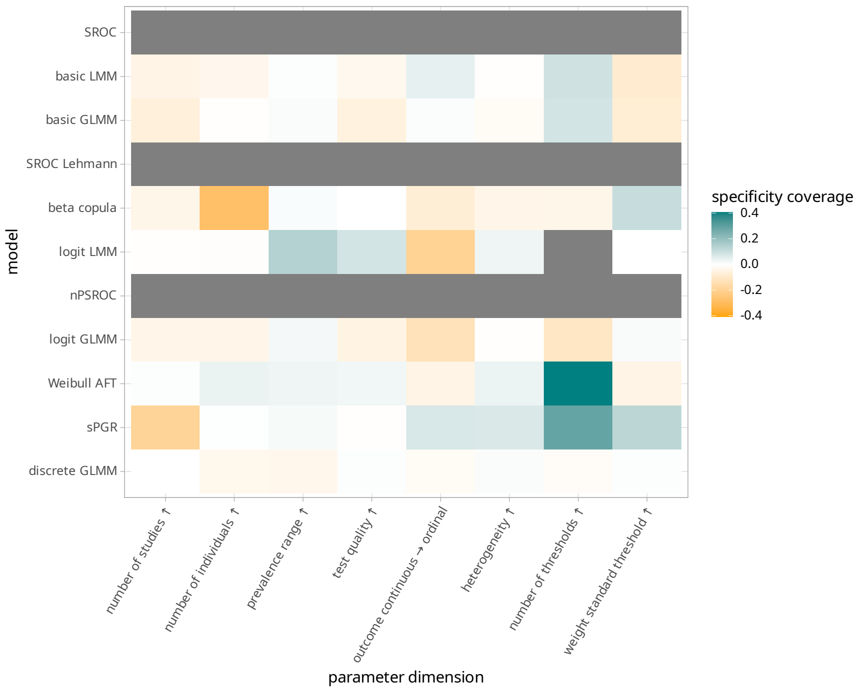

Figure S11. *Heatmap of mean change in empirical coverage of specificity when varying the parameters in the data-generating mechanism. The changes are denoted on the x-axis and displayed ceteris paribus. For the number of thresholds, the change from $T_i = 1$ to $1 \leq T_i \leq 21$ is visualized.*

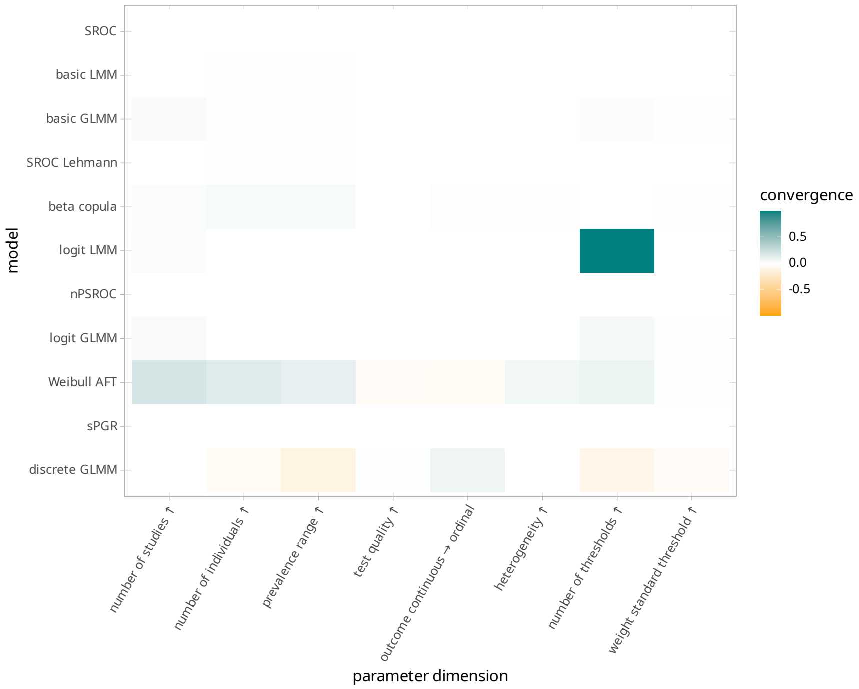

Figure S12. *Heatmap of mean change in model convergence when varying the parameters in the data-generating mechanism. The changes are denoted on the x-axis and displayed ceteris paribus. For the number of thresholds, the change from $T_i = 1$ to $1 \leq T_i \leq 21$ is visualized.*

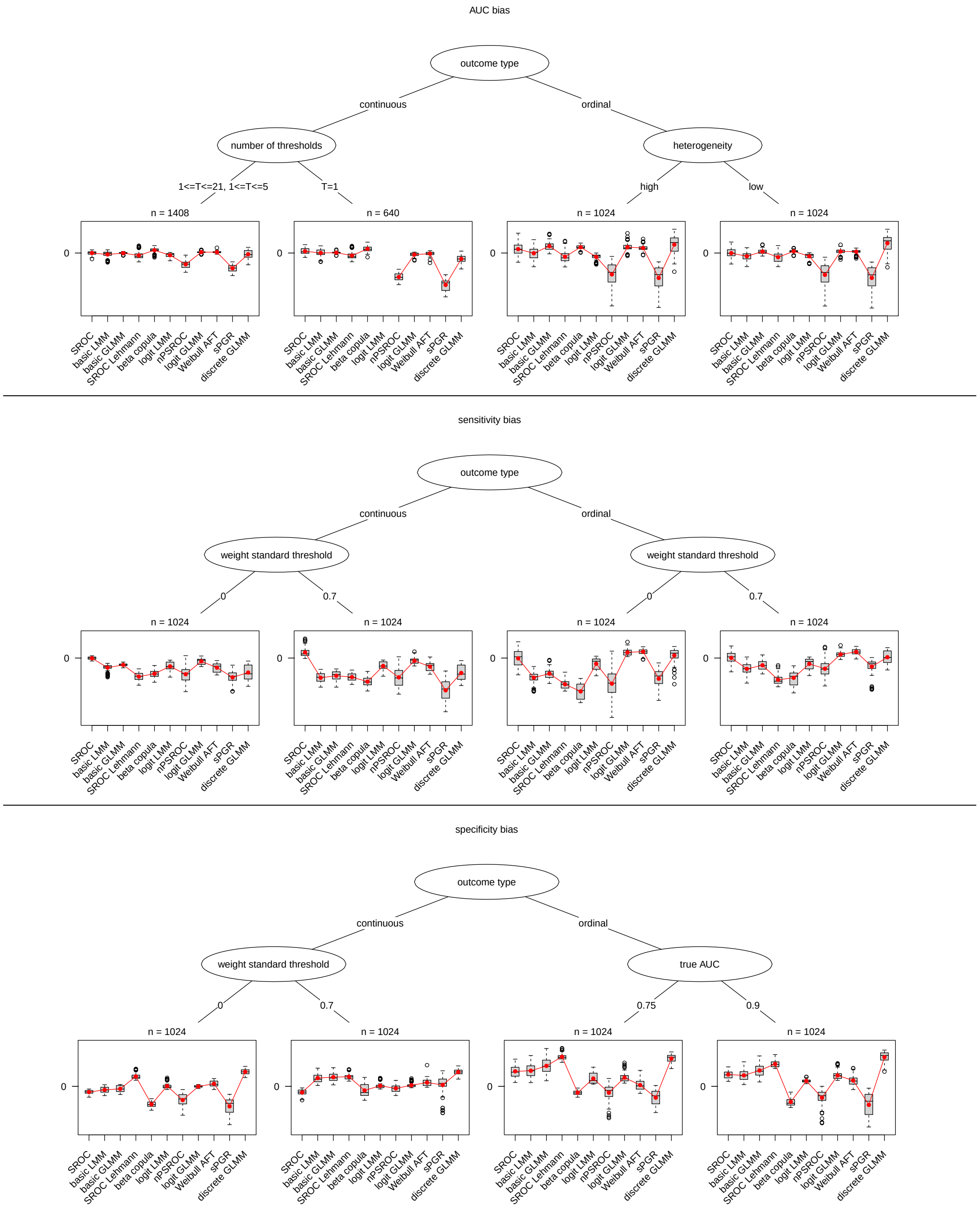

Figure S13. *Boxplots of estimated AUC, sensitivity and specificity bias, stratified using model-based recursive partitioning.*

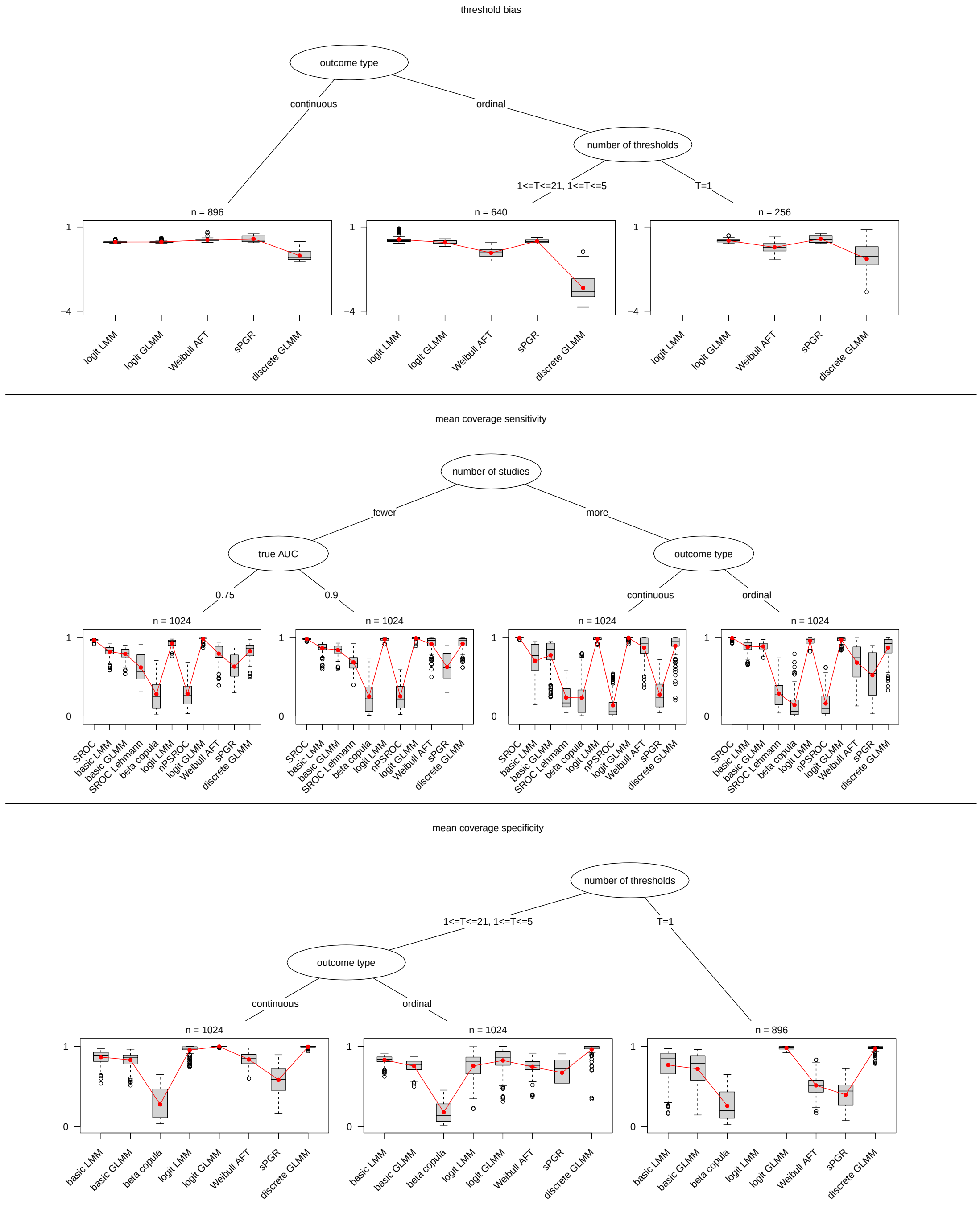

Figure S14. *Boxplots of estimated threshold bias, and sensitivity and specificity coverage, stratified using model-based recursive partitioning.*

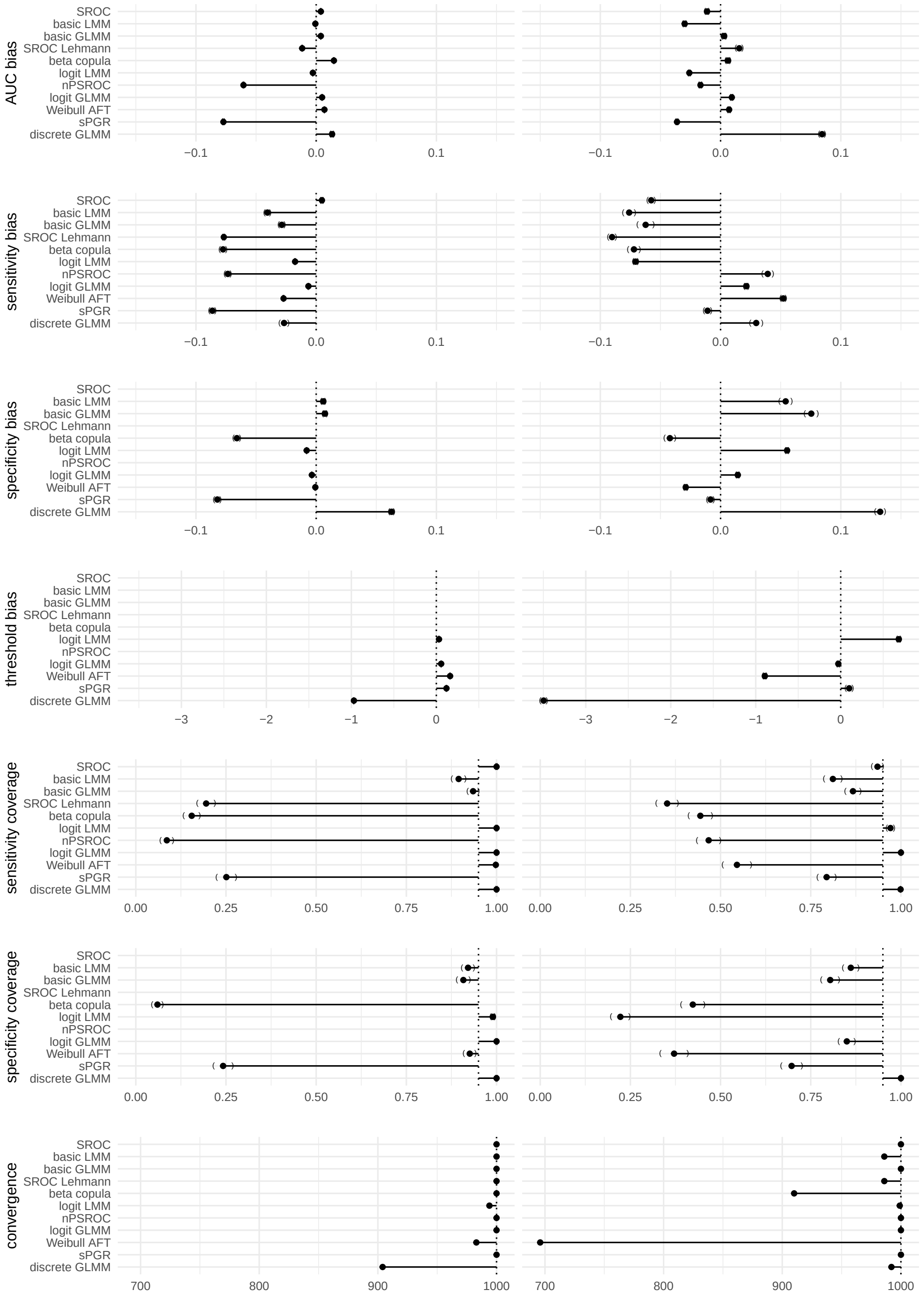

Figure S15. *Lollipop plots with* 95% *Monte Carlo CIs in parentheses of estimated model performances in two selected simulation settings. On the left, the setting with* $I \in \{7, \ldots 50\}$, $N_i \in \{100, \ldots, 3000\}$, $T_i \in \{1, \ldots, 5\}$, *continuous outcome,* $p_i \in [0.01; 0.1]$, *true AUC* $= 0.9$, *low heterogeneity, and a weight of* 0 *for a standard threshold is shown. On the right, the setting with* $I \in \{7, \ldots 50\}$, $N_i \in \{20, \ldots, 500\}$, $T_i \in \{1, \ldots, 21\}$, *ordinal outcome,* $p_i \in [0.01; 0.1]$, *true AUC* $= 0.75$, *low heterogeneity, and a weight of* 0.7 *for a standard threshold is shown.*

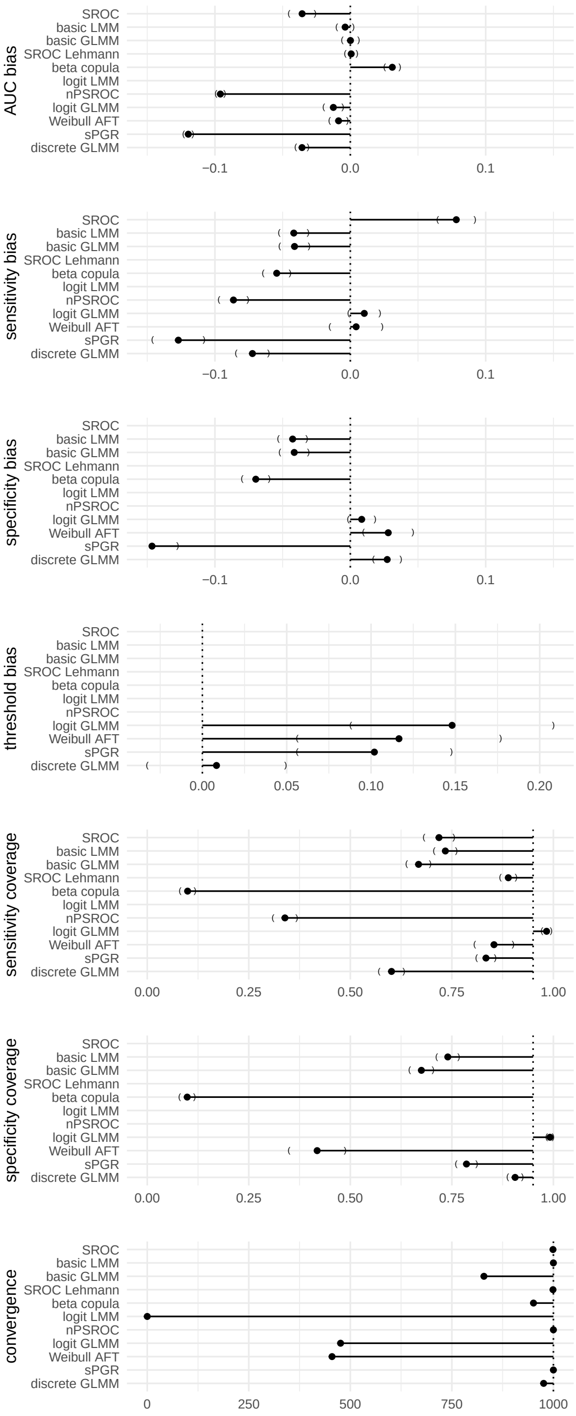

Figure S16. *Lollipop plots with* 95% *Monte Carlo CIs in parentheses of estimated model performances in an additional simulation setting with between two and three studies per dataset ($I \in \{2, 3\}$). The remaining simulation parameter choices are: $N_i \in \{20, \ldots, 500\}$, $T_i = 1$, continuous outcome, $p_i \in [0.3; 0.5]$, true AUC =* 0.75*, low heterogeneity, and a weight of* 0 *for a standard threshold.*

Table S4. *Pooled bias coverage and convergence results (multiplied by 100, except for the threshold, and rounded to two decimals) for continuous outcome settings. Maximum Monte Carlo standard errors on original scale are* 0.0003 *(bias in AUC, sensitivity, specificity),* 0.0012 *(bias in threshold),* 0.0011 *(coverage).*

	bias				coverage		
**model**	**AUC**	**sensitivity**	**specificity**	**threshold**	**sensitivity**	**specificity**	**convergence**
SROC [4]	0.32	1.29	−2.54		98.53		99.48
basic LMM [13]	−0.36	− 6.89	0.99		76.46	80.53	99.77
basic GLMM [18]	0.05	− 5.77	1.37		79.71	77.08	98.13
SROC Lehmann [24]	−0.99	− 8.86	4.10		43.75		99.78
beta copula [31]	1.32	− 9.28	−4.69		27.44	27.26	97.54
logit LMM [34]	−0.88	− 3.80	0.17	0.10	97.77	95.51	98.20
nPSROC [37]	−6.25	− 8.43	−3.35		20.14		100.00
logit GLMM [38]	0.10	− 1.40	0.17	0.10	99.48	99.50	98.13
Weibull AFT [39]	0.19	− 4.46	1.32	0.23	89.22	74.57	86.25
sPGR [43]	−8.36	−12.17	−3.95	0.29	45.23	51.37	100.00
discrete GLMM [42]	−1.20	− 6.80	5.96	−0.68	89.29	98.86	81.13

Table S5. *Pooled bias coverage and convergence results (multiplied by 100, except for the threshold, and rounded to two decimals) for ordinal outcome settings. Maximum Monte Carlo standard errors on original scale are* 0.0004 *(bias in AUC, sensitivity, specificity),* 0.0044 *(bias in threshold),* 0.0012 *(coverage).*

	bias				coverage		
**model**	**AUC**	**sensitivity**	**specificity**	**threshold**	**sensitivity**	**specificity**	**convergence**
SROC [4]	0.79	0.00	5.62		98.16		99.74
basic LMM [13]	− 0.66	− 7.17	5.56		86.66	83.86	99.53
basic GLMM [18]	1.75	− 5.35	7.62		84.83	76.61	98.07
SROC Lehmann [24]	− 1.60	−11.32	10.90		47.56		99.56
beta copula [31]	1.53	−12.57	− 4.66		16.85	19.78	95.79
logit LMM [34]	− 1.43	− 2.70	2.76	0.25	94.32	75.68	98.23
nPSROC [37]	− 8.68	− 8.64	− 3.76		21.11		100.00
logit GLMM [38]	1.55	2.25	4.10	0.11	97.58	87.13	97.96
Weibull AFT [39]	1.40	3.08	1.46	−0.44	70.65	67.07	81.63
sPGR [43]	−10.01	− 6.90	− 6.32	0.20	57.07	58.77	100.00
discrete GLMM [42]	3.63	0.80	11.97	−2.03	86.93	96.66	88.91

without restrictions on the number of reported diagnostic thresholds (Figure S17) or with additional restriction on setting where each study reports a single diagnostic threshold (Figure S18). The visualizations are generated using using Model-based Recursive Partitioning with the `partykit` R-package [45, 46]. In red, the fitted values from the models are drawn.

F. Additional results on HbA1c case study

Table S6 – Table S8 show estimated optimal sensitivities, specificities, AUCs, and diagnostic thresholds, if applicable, along with 95%-CIs from a non-parametric bootstrap with 1000 replicates for the HbA_{1c} dataset. Additionally to results corresponding to an unweighted Youden-index which are shown in the main manuscript and Table S7, we also show the results using a weighted Youden-index, $2 \cdot (\lambda \cdot Se + (1-\lambda) \cdot Sp) - 1$, with $\lambda = 0.2$ (Table S6, resulting in higher weight of specificity) and $\lambda = 0.8$ (Table S8, resulting in higher weight of sensitivity). Figure S19 and Figure S20 visualize Table S6 and Table S8 in form of forest plots.

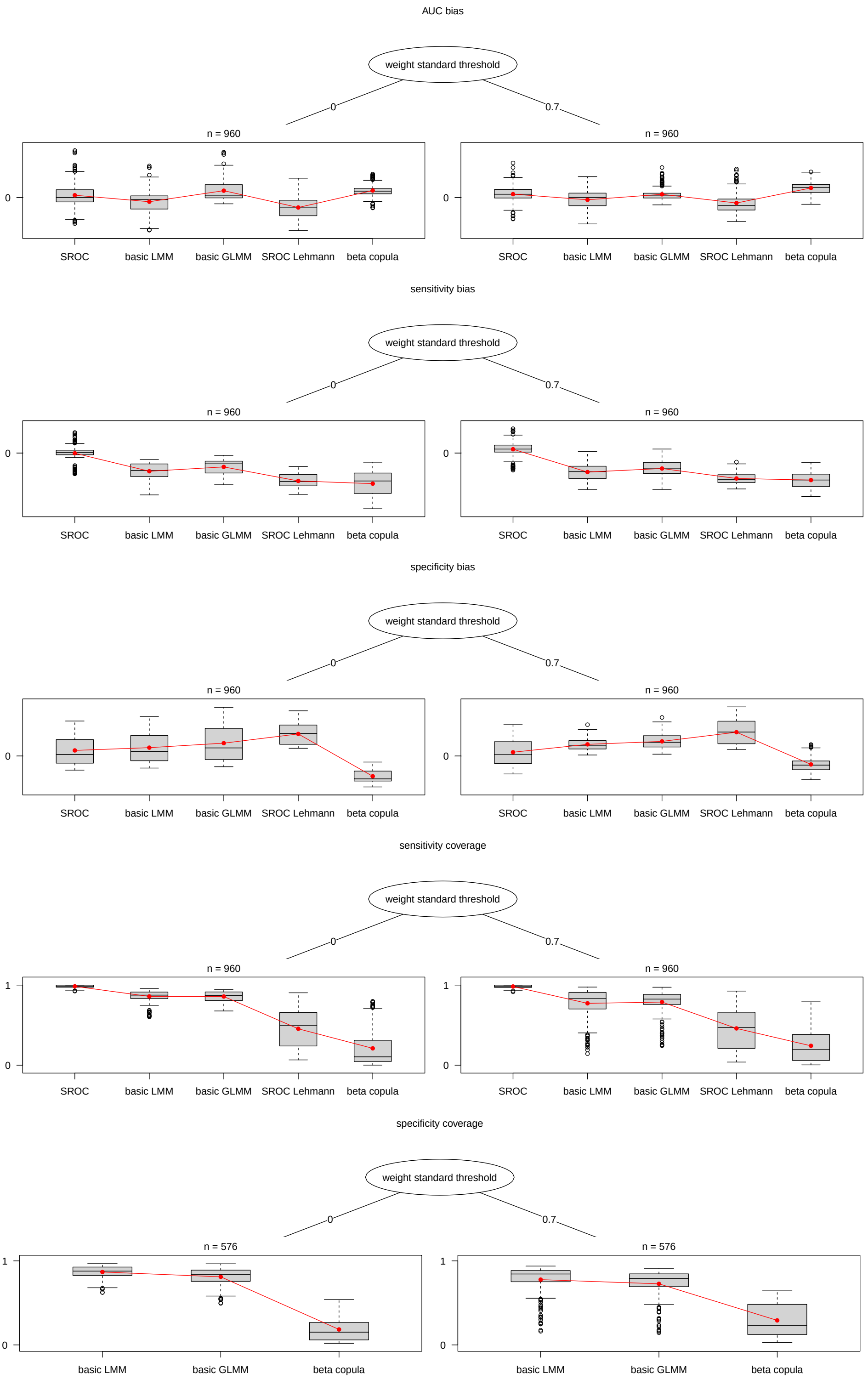

Figure S17. *Boxplots of estimated bias and empirical coverage for STM, distinguishing between the two variations in weights for a standard diagnostic threshold during data generation.*

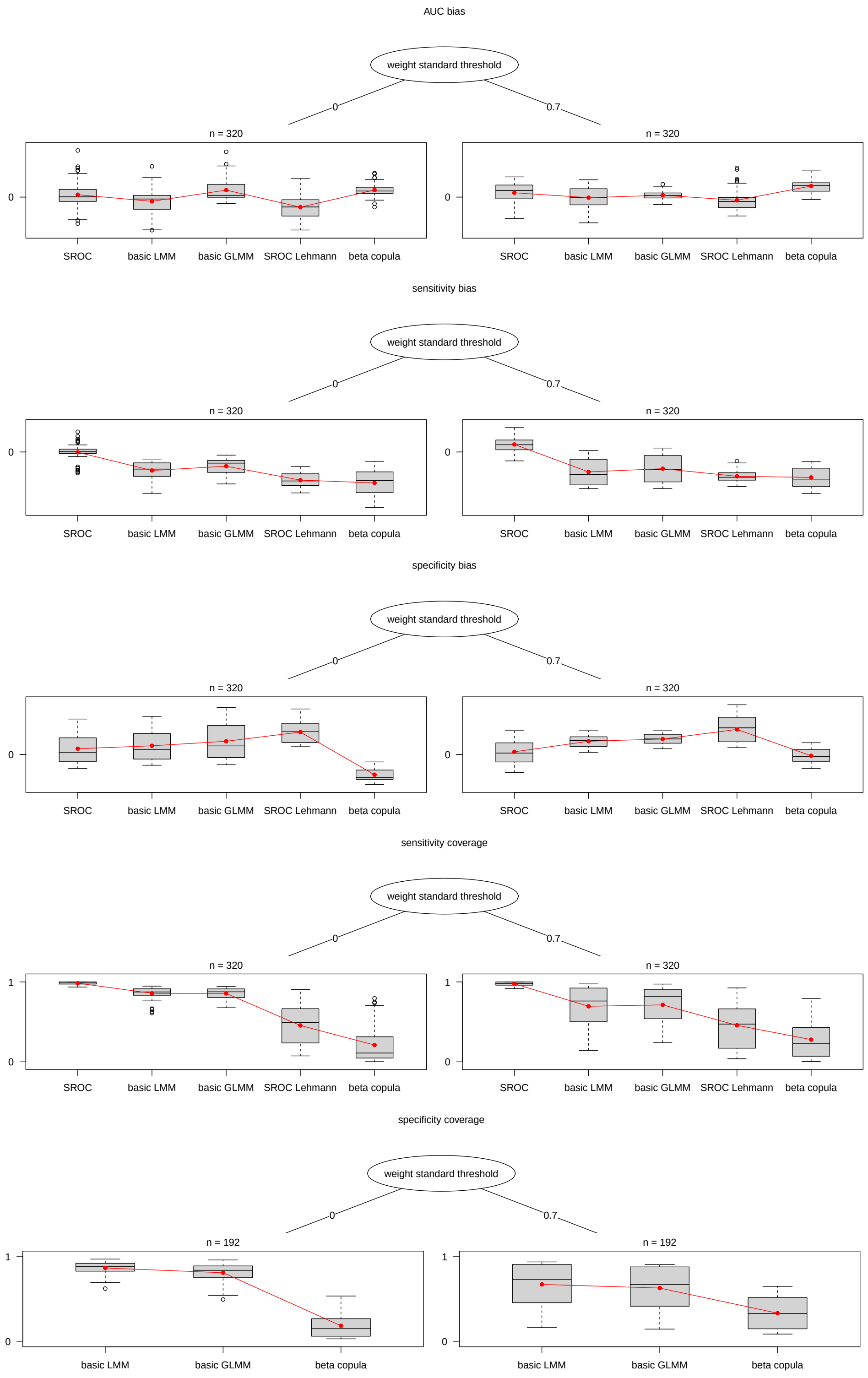

Figure S18. *Boxplots of estimated bias and empirical coverage for STM, distinguishing between the two variations in weights for a standard diagnostic threshold during data generation, only for settings where a single threshold was reported ($T = 1$).*

Table S6. *Summary results of application to type-2 diabetes meta-analysis data. Where available from the methods, the estimated optimal sensitivities, specificities and diagnostic thresholds according to the Youden-index with* 0.2 *as weight for sensitivity and* 0.8 *as weight for specificity are reported. All confidence intervals are computed using a non-parametric bootstrap on the study indices. Thresholds are rounded to two decimals. All other values are multiplied with* 100 *and rounded to one decimal.*

**model**	**threshold** [95%-CI]	**sensitivity** [95%-CI]	**specificity** [95%-CI]	**AUC** [95%-CI]
SROC [4]		47.6 [38.3; 54.2]	93.0 [91.0; 96.0]	84.4 [81.2; 86.9]
basic LMM [13]		72.1 [67.5; 77.1]	80.9 [76.0; 84.6]	83.1 [80.0; 85.7]
basic GLMM [18]		72.8 [68.0; 77.9]	81.1 [76.1; 84.7]	81.3 [73.2; 85.0]
SROC Lehmann [24]		65.9 [62.8; 69.4]	85.9 [83.9; 87.9]	82.4 [79.7; 85.2]
beta copula [31]		72.4 [66.2; 76.7]	77.9 [65.2; 83.8]	85.9 [77.7; 89.4]
logit LMM [34]	6.24 [6.09; 6.41]	43.0 [36.7; 49.8]	96.1 [94.6; 96.7]	79.9 [77.1; 84.0]
nPSROC [37]		32.7 [24.4; 41.2]	95.1 [93.7; 96.0]	77.3 [74.6; 79.9]
logit GLMM [38]	6.19 [6.07; 6.34]	50.8 [43.9; 57.7]	95.6 [94.9; 96.5]	84.4 [81.4; 87.5]
Weibull AFT [39]	6.25 [6.06; 6.63]	49.8 [31.0; 59.6]	98.1 [97.3; 98.7]	83.2 [75.2; 86.6]
sPGR [43]	6.4 [6.1 ; 7.6]	40.8 [00.4; 57.6]	91.3 [88.7; 100.0]	76.1 [73.1; 79.9]
discrete GLMM [42]	5.6 [5.5 ; 5.7]	53.0 [37.9; 61.2]	97.1 [95.9; 98.9]	84.9 [81.5; 88.3]

Table S7. *Summary results of application to type-2 diabetes meta-analysis data. Where available from the methods, the estimated optimal sensitivities, specificities and diagnostic thresholds according to the Youden-index with equally weighted sensitivity and specificity are reported. All confidence intervals are computed using a non-parametric bootstrap on the study indices. Thresholds are rounded to two decimals. All other values are multiplied with* 100 *and rounded to one decimal.*

**model**	**threshold** [95%-CI]	**sensitivity** [95%-CI]	**specificity** [95%-CI]	**AUC** [95%-CI]
SROC [4]		77.1 [69.3; 83.2]	78.0 [74.0; 85.0]	84.4 [81.2; 86.9]
basic LMM [13]		72.1 [67.5; 77.1]	80.9 [76.0; 84.6]	83.1 [80.0; 85.7]
basic GLMM [18]		72.8 [68.0; 77.9]	81.1 [76.1; 84.7]	81.3 [73.2; 85.0]
SROC Lehmann [24]		65.9 [62.8; 69.4]	85.9 [83.9; 87.9]	82.4 [79.7; 85.2]
beta copula [31]		72.4 [66.2; 76.7]	77.9 [65.2; 83.8]	85.9 [77.7; 89.4]
logit LMM [34]	5.84 [5.69; 5.99]	66.3 [62.5; 73.8]	83.8 [78.7; 86.9]	79.9 [77.1; 84.0]
nPSROC [37]		64.5 [59.8; 71.9]	79.7 [68.1; 84.3]	77.3 [74.6; 79.9]
logit GLMM [38]	5.89 [5.77; 6.03]	72.7 [68.3; 76.9]	84.1 [81.1; 87.2]	84.4 [81.4; 87.5]
Weibull AFT [39]	6.01 [5.82; 6.15]	65.9 [55.6; 70.8]	89.1 [80.6; 92.8]	83.2 [75.2; 86.6]
sPGR [43]	5.7 [5.6 ; 6]	68.6 [58.6; 73.5]	76.1 [70.4; 86.7]	76.1 [73.1; 79.9]
discrete GLMM [42]	5.5 [5.3 ; 5.6]	64.6 [60.8; 76.2]	91.5 [80.4; 92.5]	84.9 [81.5; 88.3]

G. Additional results on HADS-A case study

Table S9 – Table S11 show estimated optimal sensitivities, specificities, AUCs, and diagnostic thresholds, if applicable, along with 95%-CIs from a non-parametric bootstrap with 1000 replicates for the HADS-A dataset. Additionally to results corresponding to an unweighted Youden-index which are shown in the main manuscript and Table S10, we also show the results using a weighted Youden-index, $2 \cdot (\lambda \cdot \mathrm{Se} + (1-\lambda) \cdot \mathrm{Sp}) - 1$, with $\lambda = 0.2$ (Table S9, resulting in higher weight of specificity) and $\lambda = 0.8$ (Table S11, resulting in higher weight of sensitivity). Figure S21 and Figure S22 visualize Table S9 and Table S11 in form of forest plots.

Table S8. *Summary results of application to type-2 diabetes meta-analysis data. Where available from the methods, the estimated optimal sensitivities, specificities and diagnostic thresholds according to the Youden-index with* 0.8 *as weight for sensitivity and* 0.2 *as weight for specificity are reported. All confidence intervals are computed using a non-parametric bootstrap on the study indices. Thresholds are rounded to two decimals. All other values are multiplied with* 100 *and rounded to one decimal.*

**model**	**threshold** [95%-CI]	**sensitivity** [95%-CI]	**specificity** [95%-CI]	**AUC** [95%-CI]
SROC [4]		93.3 [90.4; 100.0]	46.0 [00.0; 54.0]	84.4 [81.2; 86.9]
basic LMM [13]		72.1 [67.5; 77.1]	80.9 [76.0; 84.6]	83.1 [80.0; 85.7]
basic GLMM [18]		72.8 [68.0; 77.9]	81.1 [76.1; 84.7]	81.3 [73.2; 85.0]
SROC Lehmann [24]		65.9 [62.8; 69.4]	85.9 [83.9; 87.9]	82.4 [79.7; 85.2]
beta copula [31]		72.4 [66.2; 76.7]	77.9 [65.2; 83.8]	85.9 [77.7; 89.4]
logit LMM [34]	3.51 [3.51; 5.36]	99.8 [90.1; 99.9]	0.1 [0.0; 45.0]	79.9 [77.1; 84.0]
nPSROC [37]		100.0 [97.6; 100.0]	0.0 [0.0; 10.0]	77.3 [74.6; 79.9]
logit GLMM [38]	5.52 [3.51; 5.69]	89.7 [87.3; 100.0]	47.8 [0.0; 58.0]	84.4 [81.4; 87.5]
Weibull AFT [39]	3.51 [2.00; 5.47]	100.0 [89.5; 100.0]	0.1 [0.0; 49.3]	83.2 [75.2; 86.6]
sPGR [43]	3.9 [3.9 ; 5.0]	100.0 [98.9; 100.0]	0.0 [0.0; 4.3]	76.1 [73.1; 79.9]
discrete GLMM [42]	5.1 [4.8 ; 5.3]	93.7 [88.4; 98.8]	32.2 [8.6; 59.7]	84.9 [81.5; 88.3]

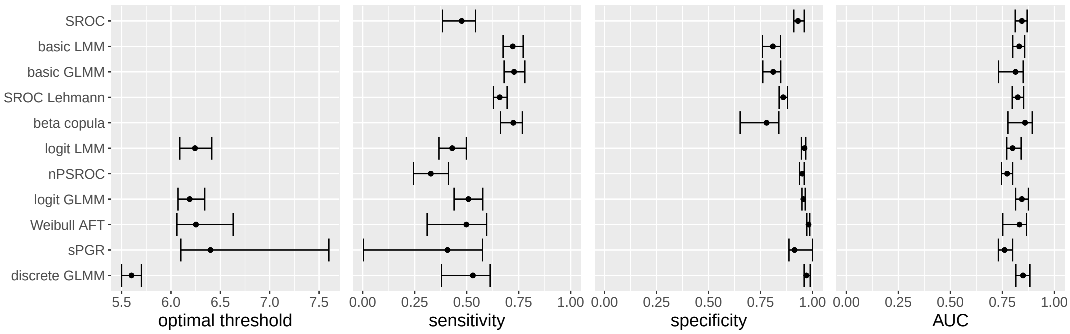

Figure S19. *Estimated optimal diagnostic thresholds, sensitivities, specificities for sensitivity weight* $\lambda = 0.2$*, and AUC for all included models on* HbA_{1c} *dataset, with* 95%*-CIs, based on a non-parametric bootstrap with* 1000 *replicates.*

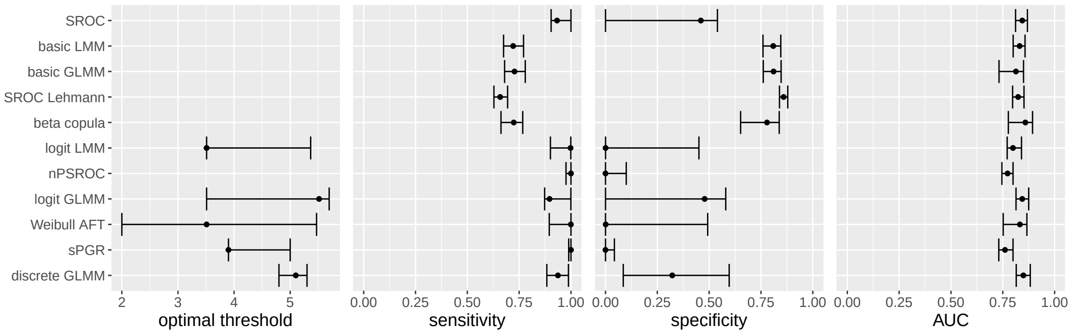

Figure S20. *Estimated optimal diagnostic thresholds, sensitivities, specificities for sensitivity weight* $\lambda = 0.8$*, and AUC for all included models on* HbA_{1c} *dataset, with* 95%*-CIs, based on a non-parametric bootstrap with* 1000 *replicates.*

Algorithm S2 Continuation of Algorithm S1.

for $j = 1, \ldots, D_i$ **do** ▷ Sample test values diseased
if outcome type == continuous **then**
if AUC == 0.75 **then**
Sample $y_{ijd} \sim \mathcal{GF}(p = 1.7473, q = 0.5027, b = 1.915 + u_{i2}, \lambda = 0.166)$
else
Sample $y_{ijd} \sim \mathcal{GF}(p = 1.7473, q = 0.5027, b = 2.029 + u_{i2}, \lambda = 0.166)$
end if
else
if AUC == 0.75 **then**
Sample $y_{ijd} \sim \mathcal{DMN}(p_0 = 0.1/22, p_1 = 0.2/22, p_2 = 0.3/22, p_3 = 0.4/22, p_4 = 0.6/22, p_5 = 0.8/22, p_6 = 0.9/22, p_7 = 1.1/22, p_8 = 1.2/22, p_9 = 1.3/22, p_{10} = 1.5/22, p_{11} = 1.7/22, p_{12} = 1.8/22, p_{13} = 1.7/22, p_{14} = 1.6/22, p_{15} = 1.4/22, p_{16} = 1.3/22, p_{17} = 1.2/22, p_{18} = 1/22, p_{19} = 0.8/22, p_{20} = 0.6/22, p_{21} = 0.5/22, \alpha)$
else
Sample $y_{ijd} \sim \mathcal{DMN}(p_0 = 0.05/22, p_1 = 0.05/22, p_2 = 0.1/22, p_3 = 0.1/22, p_4 = 0.2/22, p_5 = 0.3/22, p_6 = 0.4/22, p_7 = 0.6/22, p_8 = 0.8/22, p_9 = 0.9/22, p_{10} = 1.2/22, p_{11} = 1.4/22, p_{12} = 1.7/22, p_{13} = 2/22, p_{14} = 2.1/22, p_{15} = 2.1/22, p_{16} = 2/22, p_{17} = 1.8/22, p_{18} = 1.6/22, p_{19} = 1.3/22, p_{20} = 0.8/22, p_{21} = 0.5/22, \alpha)$
end if
end if
end for
Sample $T_i \in T_r$ ▷ Number of thresholds
for $k = 1, \ldots, T_{\max}$ **do**
if outcome type == continuous **then**
Sample $t_{ki} \in \{4.5, 4.6, \ldots, 7.5\}$ without replacement
else
Sample $t_{ki} \in \{1, 2, \ldots, 21\}$ without replacement
end if
end for
if outcome type == continuous **then**
Randomly substitute one t_{ki} with 6.5 with probability w
else
Randomly substitute one t_{ki} with 10 with probability w
end if
Sort thresholds in ascending order
Randomly select one threshold for the STM
for $k = 1, \ldots, T_{\max}$ **do**
Compute TP_{ik}, FN_{ik}, FP_{ik}, TN_{ik}
end for
Create meta-analysis dataset, containing study index, thresholds, TP, FN, FP, TN, indicator of STM threshold
end for
return Meta-analysis dataset for replicate
end for
return Meta-analysis datasets of all replicates for a simulation setting
end for
end for
end for
end for
end for
end for
end for
end for
return Meta-analysis datasets for all replicates of all 384 simulation settings

Table S9. *Summary results of application to HADS-A meta-analysis data. Where available from the methods, the estimated optimal sensitivities, specificities and diagnostic thresholds according to the Youden-index with* 0.2 *as weight for sensitivity and* 0.8 *as weight for specificity are reported. All confidence intervals are computed using a non-parametric bootstrap on the study indices. Thresholds are rounded to two decimals. All other values are multiplied with* 100 *and rounded to one decimal.*

**model**	**threshold** [95%-CI]	**sensitivity** [95%-CI]	**specificity** [95%-CI]	**AUC** [95%-CI]
SROC [4]		39.5 [33.0; 52.3]	94.0 [89.0; 96.0]	82.2 [77.1; 85.6]
basic LMM [13]		59.3 [54.5; 64.6]	84.6 [82.2; 86.6]	80.4 [75.9; 83.7]
basic GLMM [18]		60.7 [55.4; 66.6]	85.7 [83.2; 87.9]	78.4 [68.9; 83.4]
SROC Lehmann [24]		61.8 [59.5; 65.3]	83.9 [81.9; 85.9]	79.2 [76.7; 82.1]
beta copula [31]		59.9 [55.1; 65.0]	83.2 [80.2; 86.2]	83.3 [79.9; 86.6]
logit LMM [34]	12.23 [11.82; 12.76]	35.0 [29.4; 40.8]	94.5 [94.0; 95.0]	80.3 [78.5; 82.1]
nPSROC [37]		34.0 [20.4; 43.1]	93.7 [92.2; 96.5]	80.2 [78.1; 82.3]
logit GLMM [38]	11.47 [11.12; 11.91]	41.7 [36.3; 47.1]	94.0 [93.6; 94.5]	82.6 [80.8; 84.5]
Weibull AFT [39]	12.28 [11.70; 12.90]	35.9 [30.0; 42.2]	95.1 [94.3; 95.7]	82.5 [80.2; 84.7]
sPGR [43]	14 [12 ; 15]	27.2 [17.2; 45.7]	94.5 [90.6; 96.7]	78.4 [76.4; 81.2]
discrete GLMM [42]	7 [7 ; 7]	56.3 [49.3; 63.3]	97.1 [95.9; 98.2]	87.3 [84.9; 90.5]

Table S10. *Summary results of application to HADS-A meta-analysis data. Where available from the methods, the estimated optimal sensitivities, specificities and diagnostic thresholds according to the Youden-index with equally weighted sensitivity and specificity are reported. All confidence intervals are computed using a non-parametric bootstrap on the study indices. Thresholds are rounded to two decimals. All other values are multiplied with* 100 *and rounded to one decimal.*

**model**	**threshold** [95%-CI]	**sensitivity** [95%-CI]	**specificity** [95%-CI]	**AUC** [95%-CI]
SROC [4]		75.1 [62.7; 85.1]	76.0 [72.0; 84.0]	82.2 [77.1; 85.6]
basic LMM [13]		59.3 [54.5; 64.6]	84.6 [82.2; 86.6]	80.4 [75.9; 83.7]
basic GLMM [18]		60.7 [55.4; 66.6]	85.7 [83.2; 87.9]	78.4 [68.9; 83.4]
SROC Lehmann [24]		61.8 [59.5; 65.3]	83.9 [81.9; 85.9]	79.2 [76.7; 82.1]
beta copula [31]		59.9 [55.1; 65.0]	83.2 [80.2; 86.2]	83.3 [79.9; 86.6]
logit LMM [34]	8.09 [7.77; 8.44]	72.2 [69.9; 74.3]	75.6 [73.1; 78.2]	80.3 [78.5; 82.1]
nPSROC [37]		73.6 [69.0; 77.4]	74.1 [71.1; 77.3]	80.2 [78.1; 82.3]
logit GLMM [38]	8.14 [7.84; 8.46]	74.5 [72.4; 76.6]	77.3 [75.3; 79.5]	82.6 [80.8; 84.5]
Weibull AFT [39]	7.72 [7.30; 8.10]	74.9 [72.2; 78.0]	74.7 [72.2; 77.3]	82.5 [80.2; 84.7]
sPGR [43]	8 [7 ; 9]	73.4 [66.5; 77.9]	71.9 [67.6; 79.4]	78.4 [76.4; 81.2]
discrete GLMM [42]	6 [5 ; 6]	69.9 [66.0; 81.0]	90.9 [80.8; 92.6]	87.3 [84.9; 90.5]

Table S11. *Summary results of application to HADS-A meta-analysis data. Where available from the methods, the estimated optimal sensitivities, specificities and diagnostic thresholds according to the Youden-index with* 0.8 *as weight for sensitivity and* 0.2 *as weight for specificity are reported. All confidence intervals are computed using a non-parametric bootstrap on the study indices. Thresholds are rounded to two decimals. All other values are multiplied with* 100 *and rounded to one decimal.*

**model**	**threshold** [95%-CI]	**sensitivity** [95%-CI]	**specificity** [95%-CI]	**AUC** [95%-CI]
SROC [4]		93.3 [91.8; 100.0]	39.0 [0.0; 54.0]	82.2 [77.1; 85.6]
basic LMM [13]		59.3 [54.5; 64.6]	84.6 [82.2; 86.6]	80.4 [75.9; 83.7]
basic GLMM [18]		60.7 [55.4; 66.6]	85.7 [83.2; 87.9]	78.4 [68.9; 83.4]
SROC Lehmann [24]		61.8 [59.5; 65.3]	83.9 [81.9; 85.9]	79.2 [76.7; 82.1]
beta copula [31]		59.9 [55.1; 65.0]	83.2 [80.2; 86.2]	83.3 [79.9; 86.6]
logit LMM [34]	3.47 [2.59; 4.21]	93.7 [92.8; 94.8]	31.4 [23.9; 37.9]	80.3 [78.5; 82.1]
nPSROC [37]		93.1 [91.5; 95.7]	38.3 [25.7; 46.0]	80.2 [78.1; 82.3]
logit GLMM [38]	4.56 [3.98; 5.11]	93.0 [92.4; 93.7]	39.6 [33.4; 45.9]	82.6 [80.8; 84.5]
Weibull AFT [39]	3.93 [3.40; 4.50]	95.5 [95.1; 95.9]	35.3 [29.1; 42.1]	82.5 [80.2; 84.7]
sPGR [43]	3 [2 ; 5]	95.5 [91.6; 97.7]	23.8 [13.3; 43.7]	78.4 [76.4; 81.2]
discrete GLMM [42]	3 [3 ; 4]	93.9 [88.8; 94.8]	39.5 [35.9; 66.0]	87.3 [84.9; 90.5]

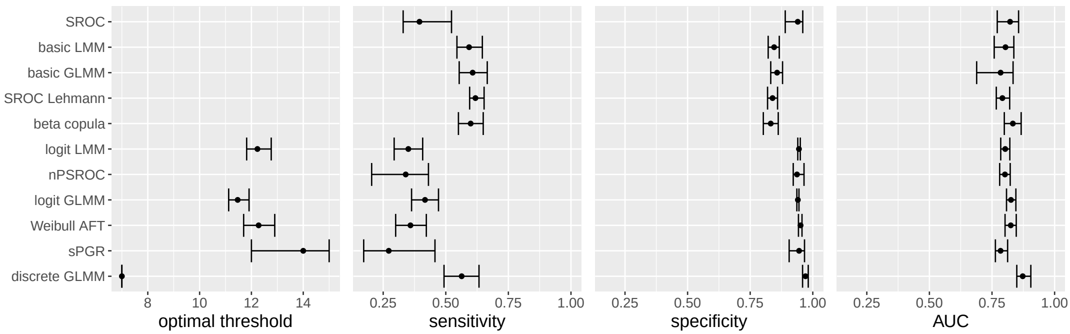

Figure S21. *Estimated optimal diagnostic thresholds, sensitivities, specificities for sensitivity weight* $\lambda = 0.2$*, and AUC for all included models on HADS-A dataset, with* 95%*-CIs, based on a non-parametric bootstrap with* 1000 *replicates.*

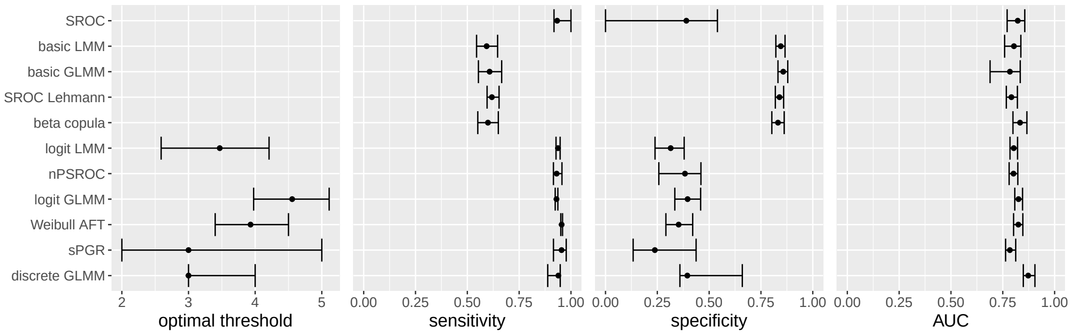

Figure S22. *Estimated optimal diagnostic thresholds, sensitivities, specificities for sensitivity weight* $\lambda = 0.8$*, and AUC for all included models on HADS-A dataset, with* 95%*-CIs, based on a non-parametric bootstrap with* 1000 *replicates.*

References

1. Hoyer A and Kuss O. Meta-analysis of full ROC curves with flexible parametric distributions of diagnostic test values. Research synthesis methods 2020;11:301–13.
2. Zhang Y and Zhou H. MGLM: Multivariate Response Generalized Linear Models. R package version 0.2.1. 2022. URL: https://CRAN.R-project.org/package=MGLM.
3. Kardaun JWPF and Kardaun OJWF. Comparative diagnostic performance of three radiological procedures for the detection of lumbar disk herniation. Methods of information in medicine 1990;29:12–22.
4. Moses LE, Shapiro D, and Littenberg B. Combining independent studies of a diagnostic test into a summary ROC curve: data-analytic approaches and some additional considerations. Statistics in medicine 1993;12:1293–316.
5. Littenberg B and Moses LE. Estimating diagnostic accuracy from multiple conflicting reports: a new meta-analytic method. Medical decision making 1993;13:313–21.
6. Hasselblad V and Hedges LV. Meta-analysis of screening and diagnostic tests. Psychological bulletin 1995;117:167.
7. Hellmich M, Abrams KR, and Sutton AJ. Bayesian approaches to meta-analysis of ROC curves. Medical Decision Making 1999;19:252–64.
8. Rutter CM and Gatsonis CA. A hierarchical regression approach to meta-analysis of diagnostic test accuracy evaluations. Statistics in medicine 2001;20:2865–84.
9. Harbord RM, Deeks JJ, Egger M, Whiting P, and Sterne JA. A unification of models for meta-analysis of diagnostic accuracy studies. Biostatistics 2007;8:239–51.
10. Kester AD and Buntinx F. Meta-analysis of ROC curves. Medical Decision Making 2000;20:430–9.
11. Dukic V and Gatsonis CA. Meta-analysis of diagnostic test accuracy assessment studies with varying number of thresholds. Biometrics 2003;59:936–46.
12. Poon WY. A latent normal distribution model for analysing ordinal responses with applications in meta-analysis. Statistics in medicine 2004;23:2155–72.
13. Reitsma JB, Glas AS, Rutjes AW, Scholten RJ, Bossuyt PM, and Zwinderman AH. Bivariate analysis of sensitivity and specificity produces informative summary measures in diagnostic reviews. Journal of clinical epidemiology 2005;58:982–90.
14. Arends L, Hamza T, Van Houwelingen J, Heijenbrok-Kal M, Hunink M, and Stijnen T. Bivariate random effects meta-analysis of ROC curves. Medical Decision Making 2008;28:621–38.
15. Schlattmann P, Verba M, Dewey M, and Walther M. Mixture models in diagnostic meta-analyses—clustering summary receiver operating characteristic curves accounted for heterogeneity and correlation. Journal of clinical epidemiology 2015;68:61–72.
16. Guolo A. A double SIMEX approach for bivariate random-effects meta-analysis of diagnostic accuracy studies. BMC Medical Research Methodology 2017;17:6.
17. Guolo A. Hierarchical multinomial processing tree models for meta-analysis of diagnostic accuracy studies. Journal of the Royal Statistical Society Series A: Statistics in Society 2024;188:410–27.
18. Chu H and Cole SR. Bivariate meta-analysis of sensitivity and specificity with sparse data: a generalized linear mixed model approach. Journal of clinical epidemiology 2006;59:1331–2.
19. Chu H, Guo H, and Zhou Y. Bivariate random effects meta-analysis of diagnostic studies using generalized linear mixed models. Medical decision making 2010;30:499–508.
20. Bipat S, Zwinderman AH, Bossuyt PMM, and Stoker J. Multivariate random-effects approach: for meta-analysis of cancer staging studies. Academic radiology 2007;14:974–84.

21. Hamza TH, Arends LR, Houwelingen HC van, and Stijnen T. Multivariate random effects meta-analysis of diagnostic tests with multiple thresholds. BMC medical research methodology 2009;9:73.
22. Guolo A and Pesantez Cabrera TE. A SIMEX approach for meta-analysis of diagnostic accuracy studies with attention to ROC curves. The International Journal of Biostatistics 2023;19:455–71.
23. Putter H, Fiocco M, and Stijnen T. Meta-analysis of diagnostic test accuracy studies with multiple thresholds using survival methods. Biometrical journal 2010;52:95–110.
24. Holling H, Böhning W, and Böhning D. Meta-analysis of diagnostic studies based upon SROC-curves: a mixed model approach using the Lehmann family. Statistical Modelling 2012;12:347–75.
25. Charoensawat S, Böhning W, Böhning D, and Holling H. Meta-analysis and meta-modelling for diagnostic problems. BMC medical research methodology 2014;14:56.
26. Holling H, Böhning W, and Böhning D. Likelihood-based clustering of meta-analytic SROC curves. Psychometrika 2012;77:106–26.
27. Doebler P, Holling H, and Böhning D. A mixed model approach to meta-analysis of diagnostic studies with binary test outcome. Psychological methods 2012;17:418.
28. Doebler P and Holling H. Meta-analysis of diagnostic accuracy and ROC curves with covariate adjusted semiparametric mixtures. Psychometrika 2015;80:1084–104.
29. Doebler P and Sousa-Pinto B. mada: Meta-Analysis of Diagnostic Accuracy. R package version 0.5.12. 2022. URL: https://CRAN.R-project.org/package=mada.
30. Chu H, Nie L, Chen Y, Huang Y, and Sun W. Bivariate random effects models for meta-analysis of comparative studies with binary outcomes: methods for the absolute risk difference and relative risk. Statistical methods in medical research 2012;21:621–33.
31. Nikoloulopoulos AK. A mixed effect model for bivariate meta-analysis of diagnostic test accuracy studies using a copula representation of the random effects distribution. Statistics in medicine 2015;34:3842–65.
32. Kuss O, Hoyer A, and Solms A. Meta-analysis for diagnostic accuracy studies: a new statistical model using beta-binomial distributions and bivariate copulas. Statistics in medicine 2014;33:17–30.
33. Riley RD, Takwoingi Y, Trikalinos T, et al. Meta-analysis of test accuracy studies with multiple and missing thresholds: a multivariate-normal model. J Biomed Biostat 2014;5:196.
34. Steinhauser S, Schumacher M, and Rücker G. Modelling multiple thresholds in meta-analysis of diagnostic test accuracy studies. BMC medical research methodology 2016;16:97.
35. Guolo A and To DK. A pseudo-likelihood approach for multivariate meta-analysis of test accuracy studies with multiple thresholds. Statistical Methods in Medical Research 2021;30:204–20.
36. Zapf A, Hoyer A, Kramer K, and Kuss O. Nonparametric meta-analysis for diagnostic accuracy studies. Statistics in Medicine 2015;34:3831–41.
37. Martínez-Camblor P. Fully non-parametric receiver operating characteristic curve estimation for random-effects meta-analysis. Statistical methods in medical research 2017;26:5–20.
38. Hoyer A and Kuss O. Meta-analysis for the comparison of two diagnostic tests to a common gold standard: a generalized linear mixed model approach. Statistical methods in medical research 2018;27:1410–21.
39. Hoyer A, Hirt S, and Kuss O. Meta-analysis of full ROC curves using bivariate time-to-event models for interval-censored data. Research synthesis methods 2018;9:62–72.

40. Jones HE, Gatsonsis CA, Trikalinos TA, Welton NJ, and Ades A. Quantifying how diagnostic test accuracy depends on threshold in a meta-analysis. Statistics in medicine 2019;38:4789–803.

41. Hoyer A and Kuss O. Meta-analysis of full ROC curves: Additional flexibility by using semi-parametric distributions of diagnostic test values. Research synthesis methods 2019;10:528–38.

42. Stoye FV, Tschammler C, Kuss O, and Hoyer A. A discrete time-to-event model for the meta-analysis of full ROC curves. Research synthesis methods 2024;15:1031–48.

43. Frömke C, Kirstein M, and Zapf A. A semiparametric approach for meta-analysis of diagnostic accuracy studies with multiple cut-offs. Research synthesis methods 2022;13:612–21.

44. Gasparini A. rsimsum: Summarise results from Monte Carlo simulation studies. Journal of Open Source Software 26 2018;3:739.

45. Zeileis A, Hothorn T, and Hornik K. Model-Based Recursive Partitioning. Journal of Computational and Graphical Statistics 2008;17:492–514.

46. Hothorn T and Zeileis A. partykit: A Modular Toolkit for Recursive Partytioning in R. Journal of Machine Learning Research 2015;16:3905–9.

47. Morris TP, White IR, and Crowther MJ. Using simulation studies to evaluate statistical methods. Statistics in medicine 2019;38:2074–102.
