# Supplementary material for: Comparison of Different Methods for the Meta‐Analysis of Diagnostic Test Accuracy Studies—A Simulation Study
Source: Biom J. 2026 Jul 2;68(4):e70147. doi: 10.1002/bimj.70147 (PMC13329219; doi:10.1002/bimj.70147)
Supplement: Supplementary file 3 — Supporting File 3: bimj70147‐sup‐0003‐simstudy_code.zip. [file BIMJ-68-e70147-s001.zip › figures/Fig_S04_all_heatmaps.pdf]

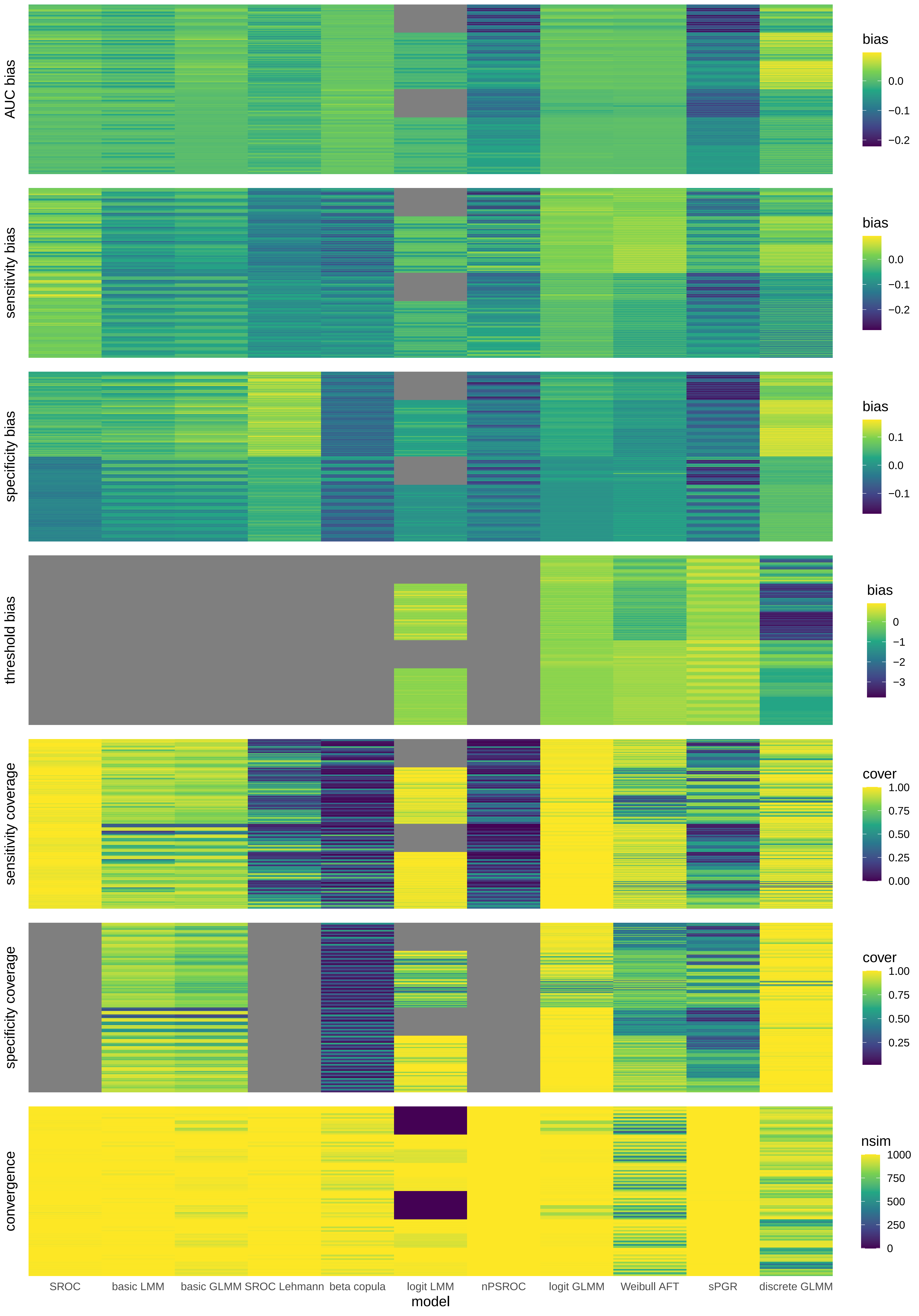
AUC bias
sensitivity bias
specificity bias
threshold bias
sensitivity coverage
specificity coverage
convergence
bias
0.0
−0.1
−0.2
bias
0.0
−0.1
−0.2
bias
0.1
0.0
−0.1
bias
0
−1
−2
−3
cover
1.00
0.75
0.50
0.25
0.00
cover
1.00
0.75
0.50
0.25
nsim
1000
750
500
250
0
SROC
basic LMM
basic GLMM
SROC Lehmann
beta copula
logit LMM
nPSROC
logit GLMM
Weibull AFT
sPGR
discrete GLMM
model
