# Supplementary figures and images for: Comparison of Different Methods for the Meta‐Analysis of Diagnostic Test Accuracy Studies—A Simulation Study
Source: Biom J. 2026 Jul 2;68(4):e70147. doi: 10.1002/bimj.70147 (PMC13329219; doi:10.1002/bimj.70147)

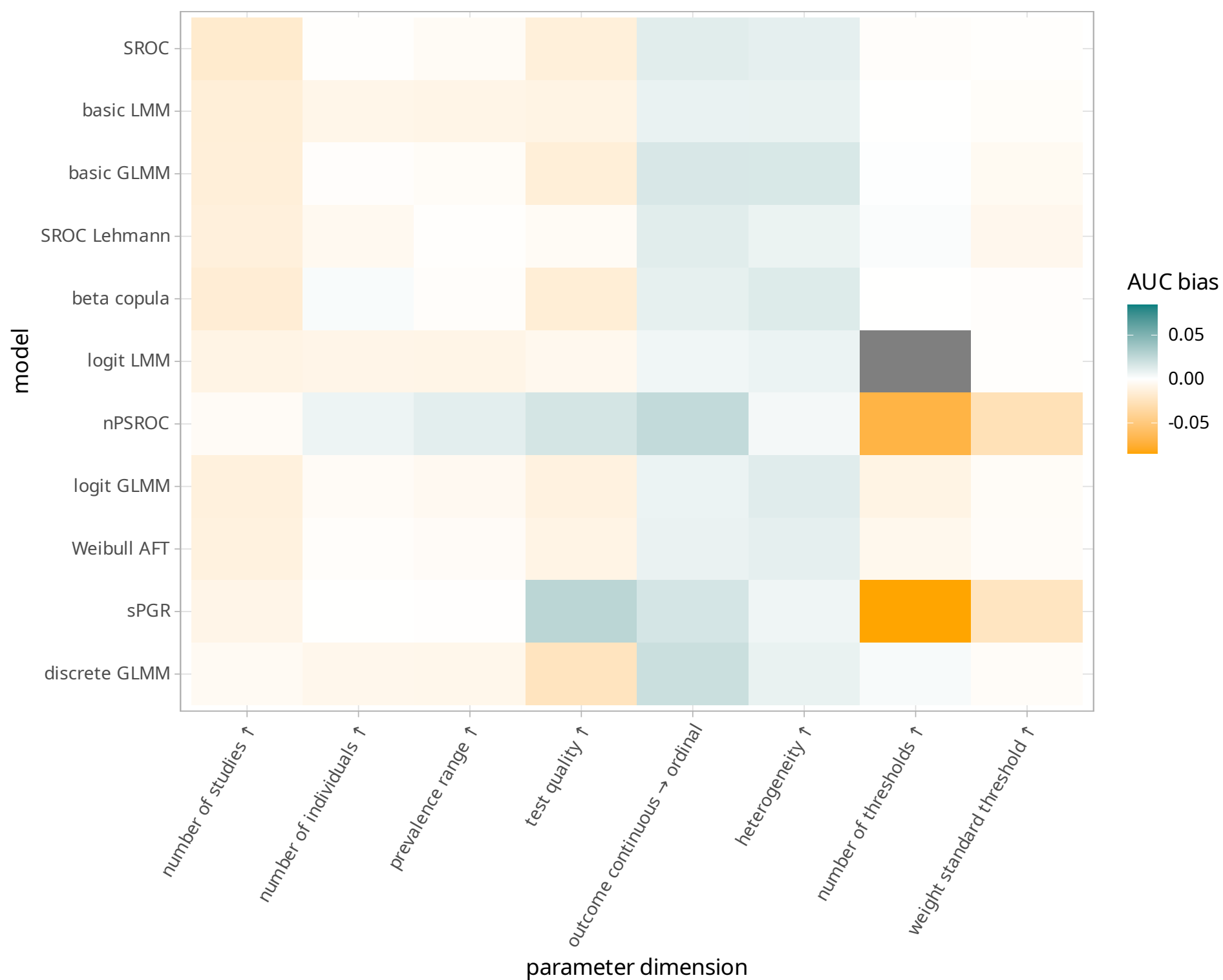

Supplement: Supplementary file 3 — Supporting File 3: bimj70147‐sup‐0003‐simstudy_code.zip. [file BIMJ-68-e70147-s001.zip › figures/Fig_01_heatmap_AUC_bias_effects.pdf]

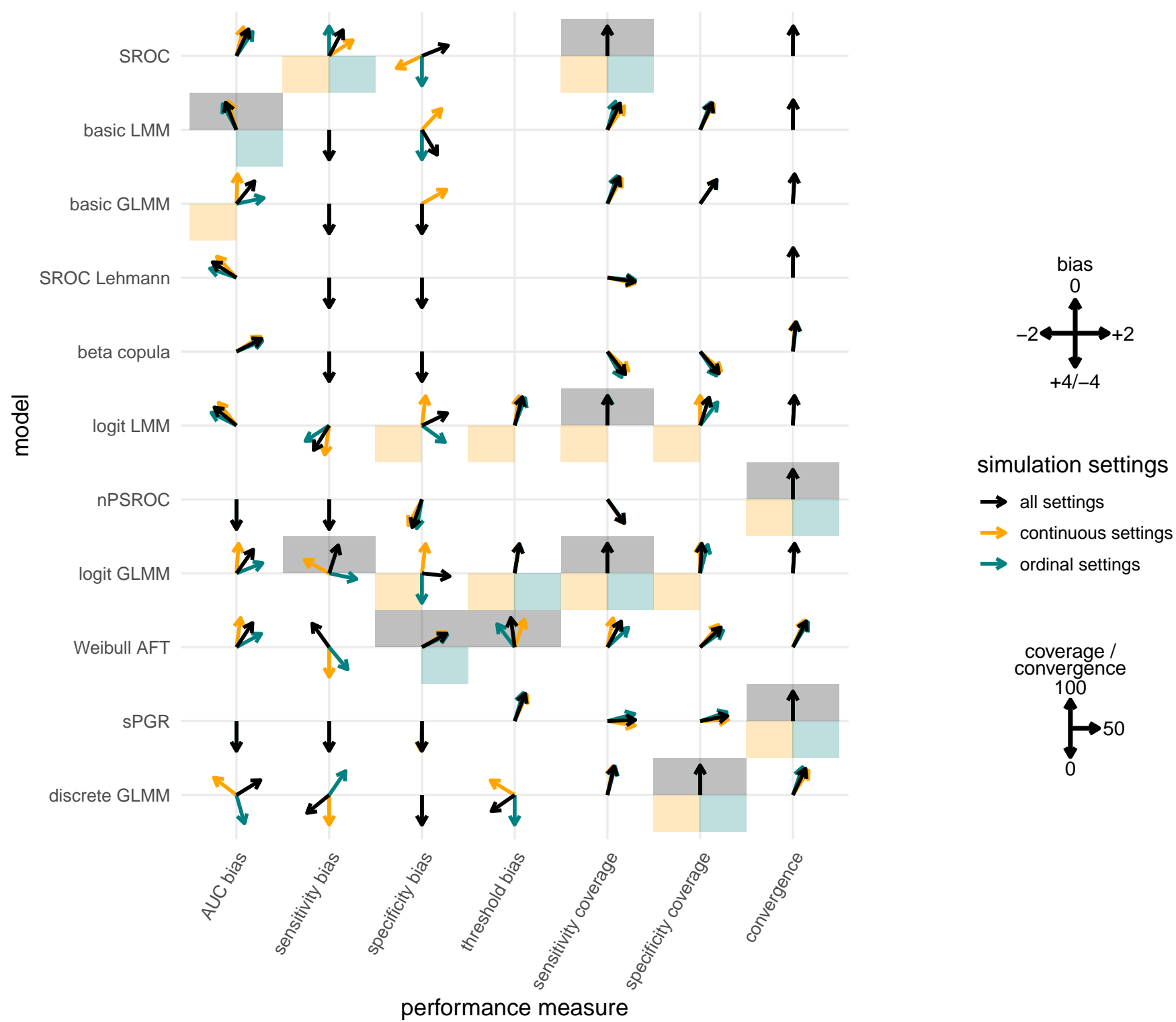

Supplement: Supplementary file 3 — Supporting File 3: bimj70147‐sup‐0003‐simstudy_code.zip. [file BIMJ-68-e70147-s001.zip › figures/Fig_02_arrowmap_all_vs_ord_vs_cont.pdf]

continuous outcome

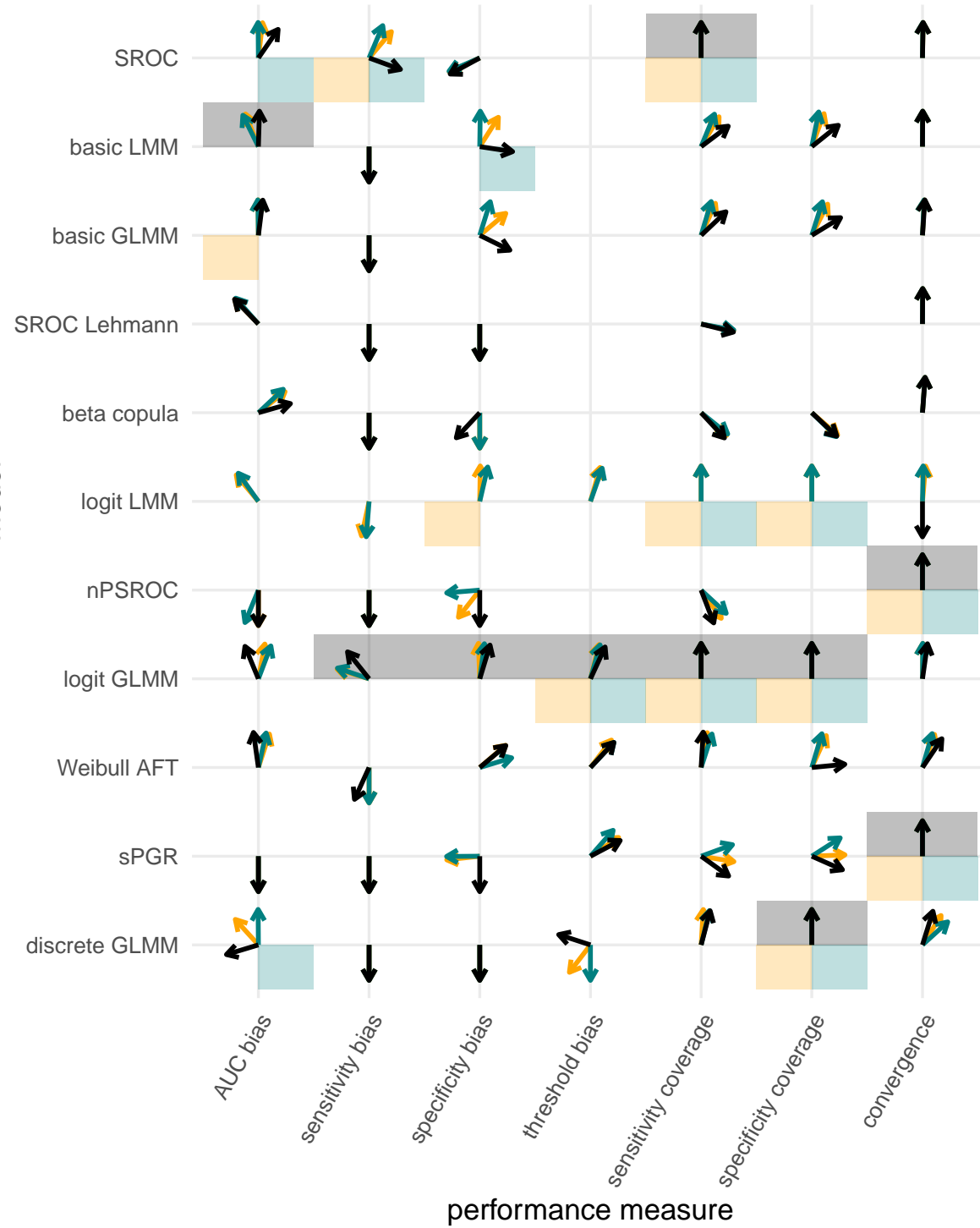

ordinal outcome

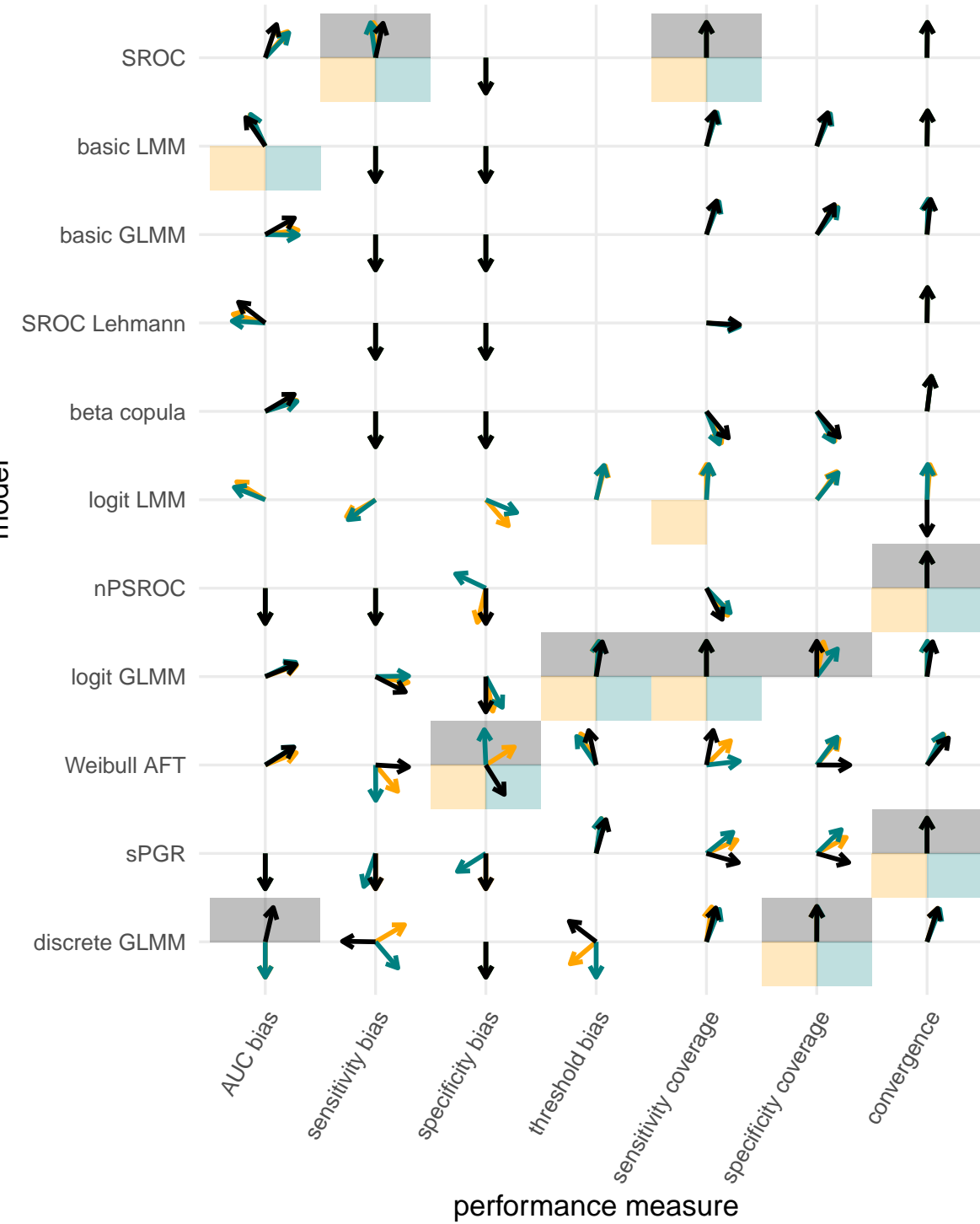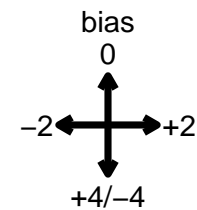

number of thresholds

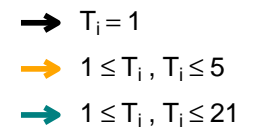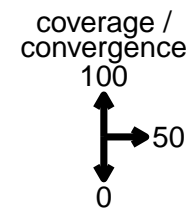

Supplement: Supplementary file 3 — Supporting File 3: bimj70147‐sup‐0003‐simstudy_code.zip. [file BIMJ-68-e70147-s001.zip › figures/Fig_03_double_arrowmap_thresholds.pdf]

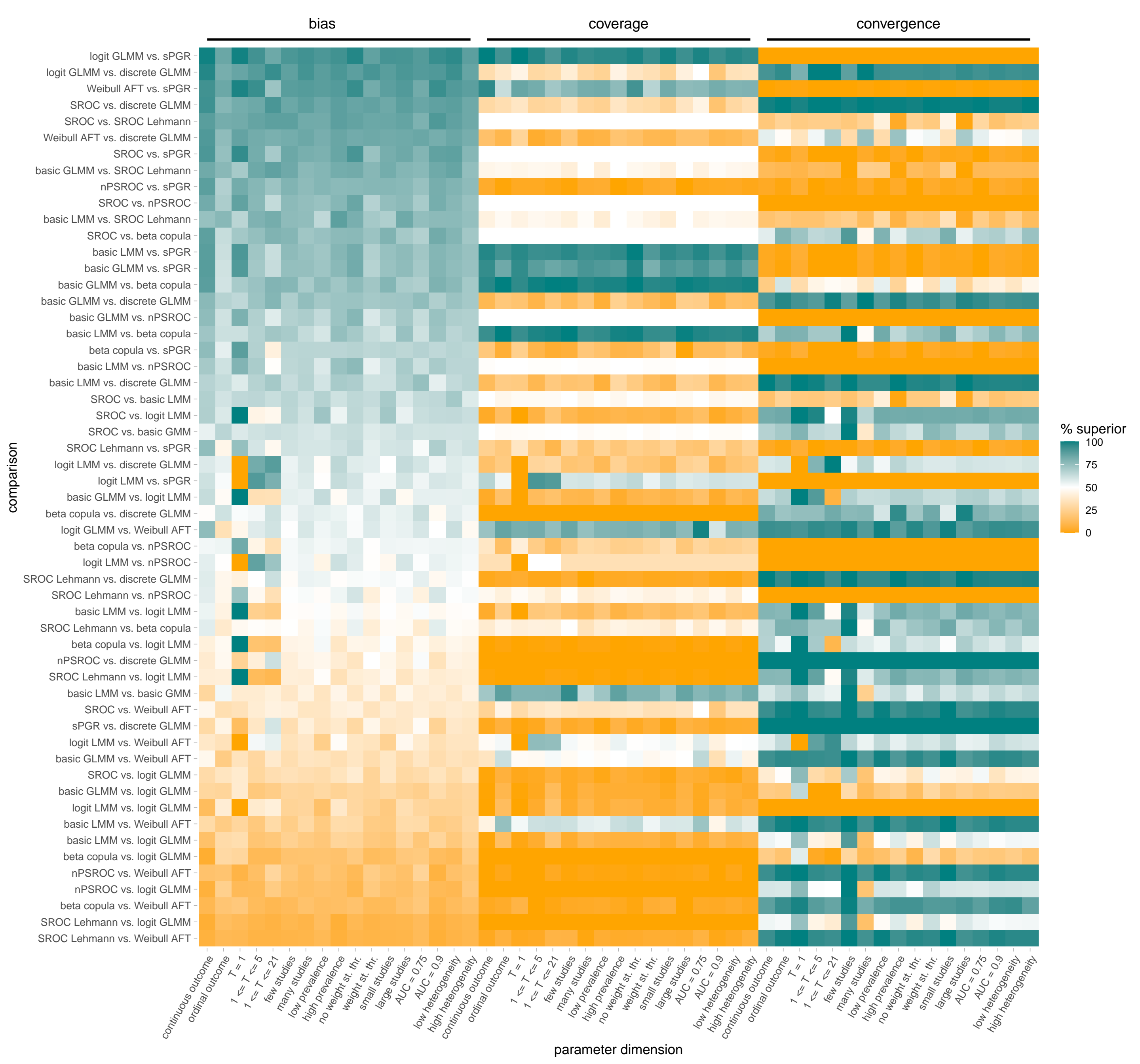

Supplement: Supplementary file 3 — Supporting File 3: bimj70147‐sup‐0003‐simstudy_code.zip. [file BIMJ-68-e70147-s001.zip › figures/Fig_04_heatmaps_headtohead.pdf]

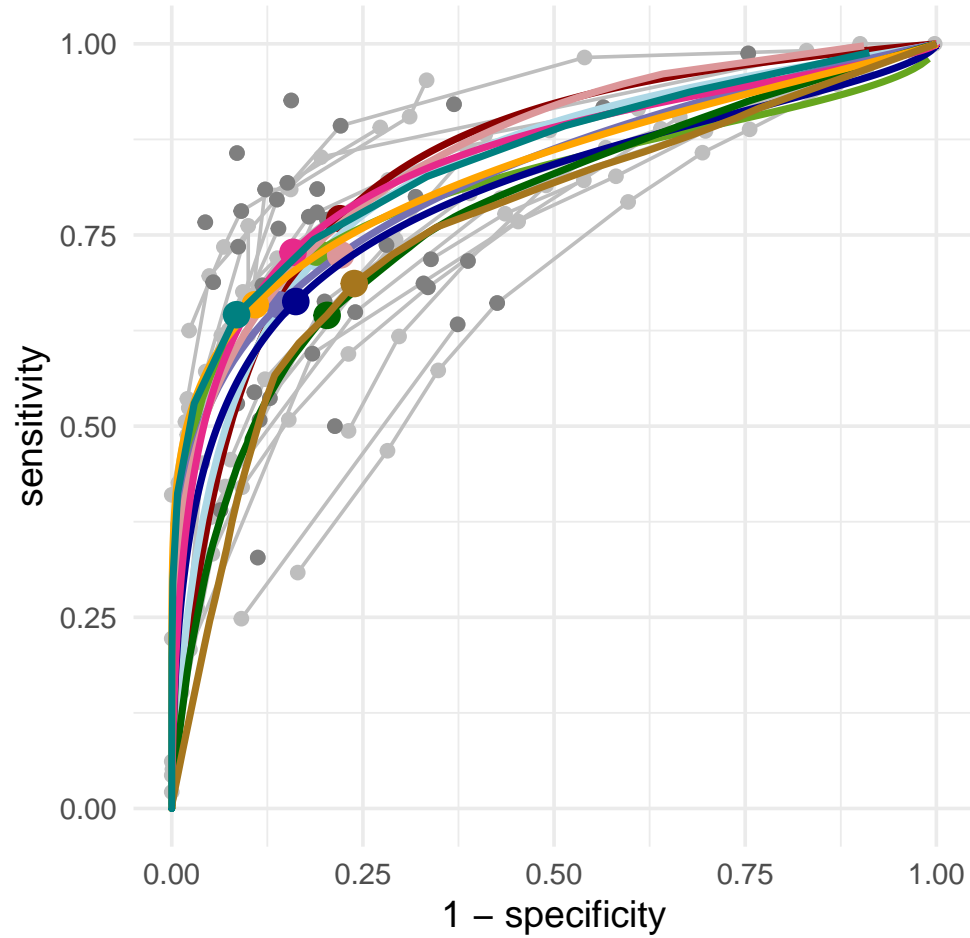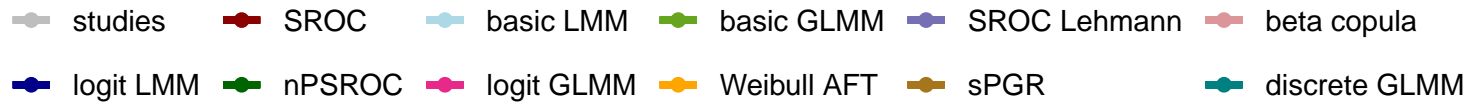

Supplement: Supplementary file 3 — Supporting File 3: bimj70147‐sup‐0003‐simstudy_code.zip. [file BIMJ-68-e70147-s001.zip › figures/Fig_05_sroc_plots_hba1c.pdf]

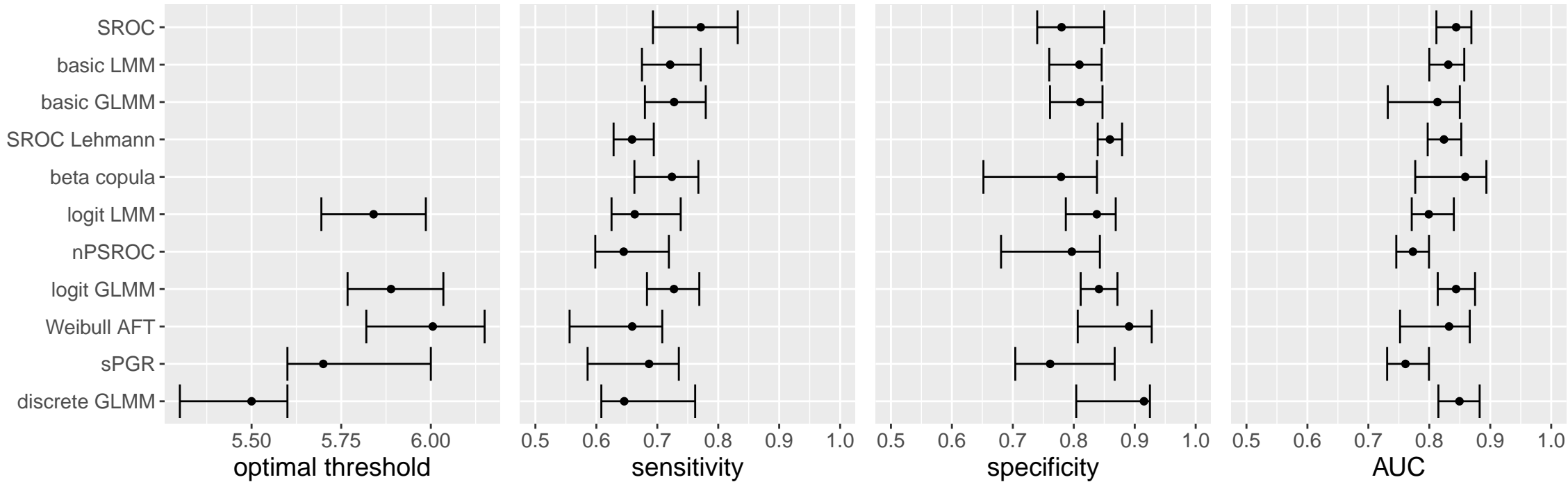

Supplement: Supplementary file 3 — Supporting File 3: bimj70147‐sup‐0003‐simstudy_code.zip. [file BIMJ-68-e70147-s001.zip › figures/Fig_06_forest_plots_hba1c_all_w_sens_0.5.pdf]

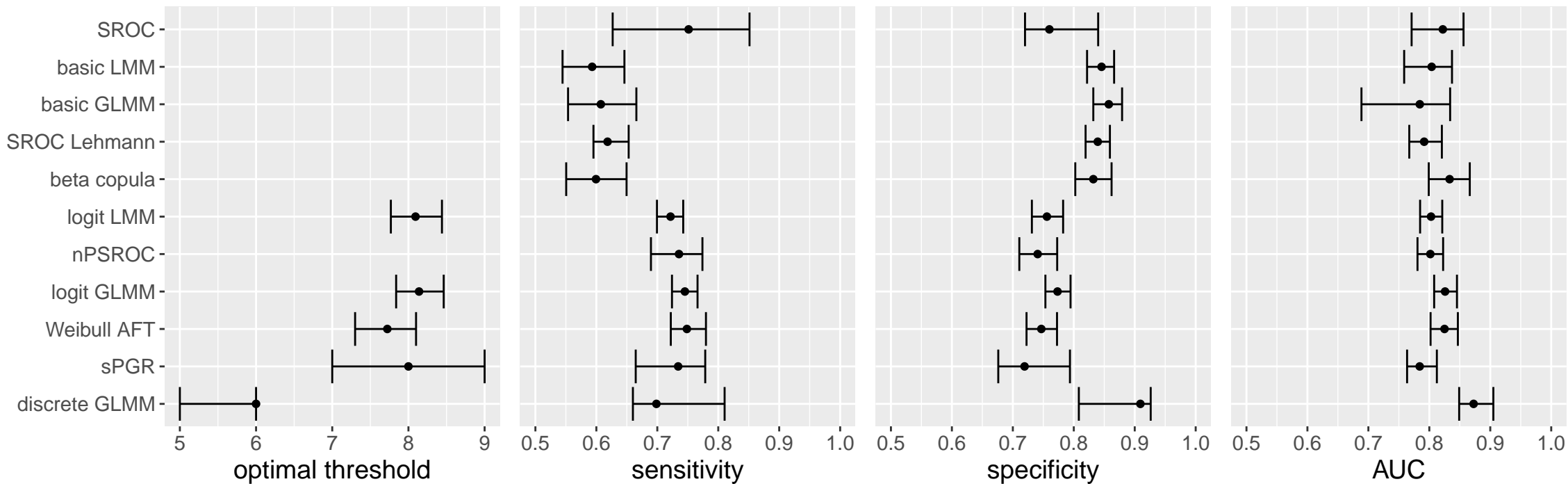

Supplement: Supplementary file 3 — Supporting File 3: bimj70147‐sup‐0003‐simstudy_code.zip. [file BIMJ-68-e70147-s001.zip › figures/Fig_08_forest_plots_hadsa_all_w_sens_0.5.pdf]

AUC = 0.75

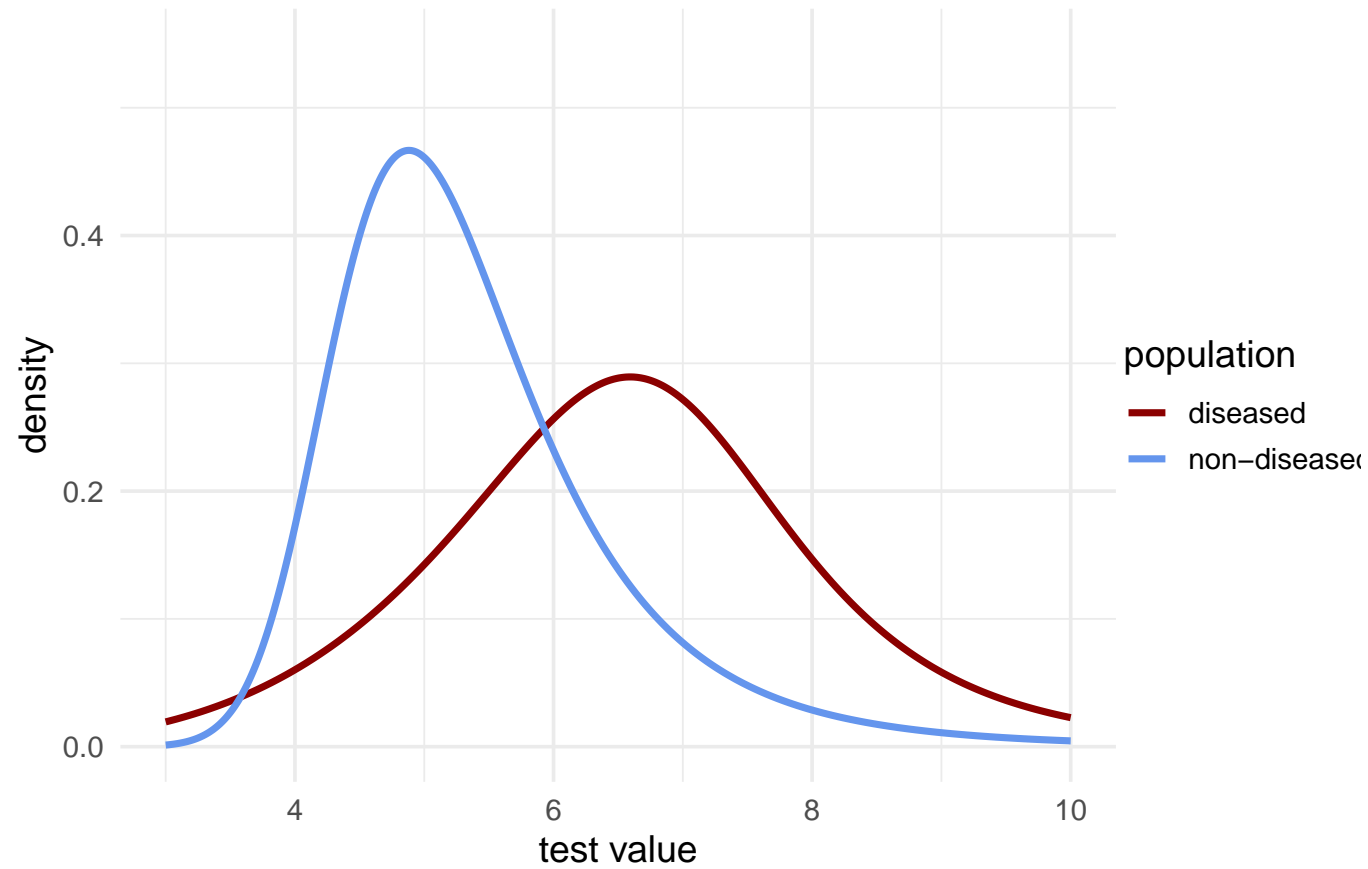

AUC = 0.90

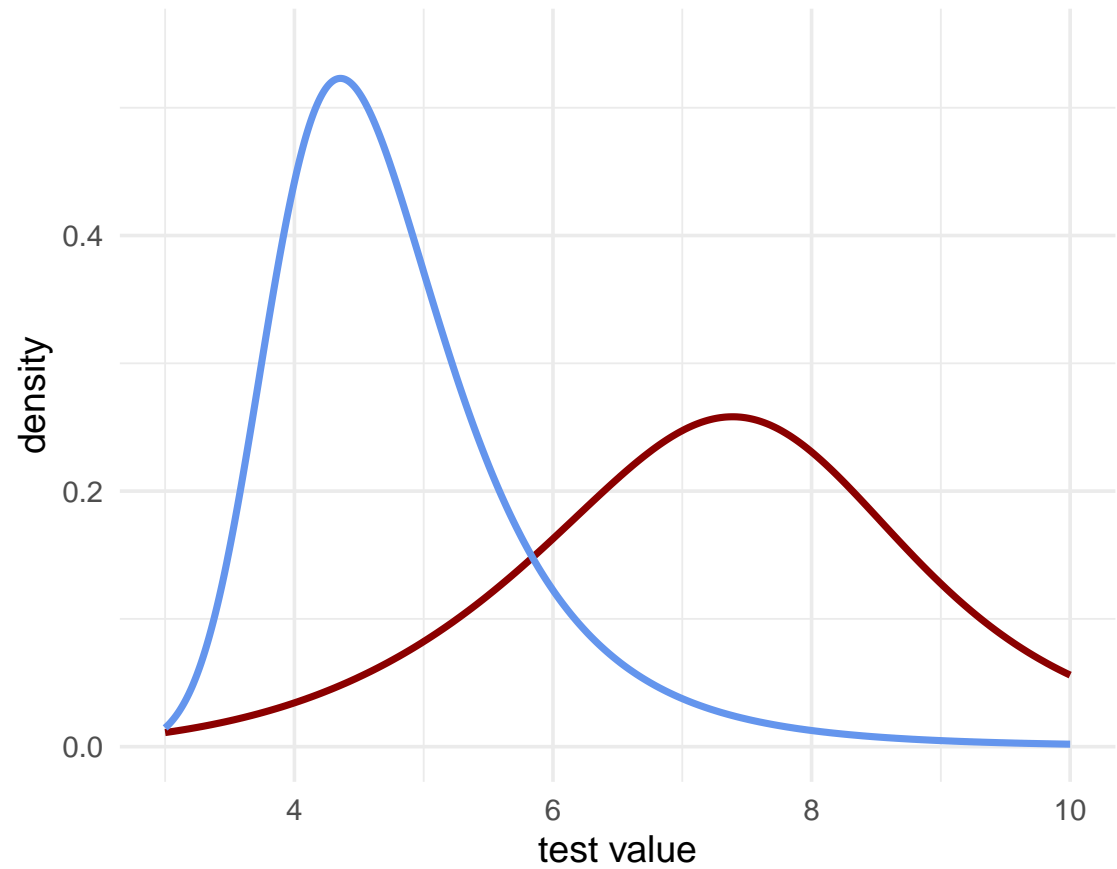

Supplement: Supplementary file 3 — Supporting File 3: bimj70147‐sup‐0003‐simstudy_code.zip. [file BIMJ-68-e70147-s001.zip › figures/Fig_S01.pdf]

AUC = 0.75

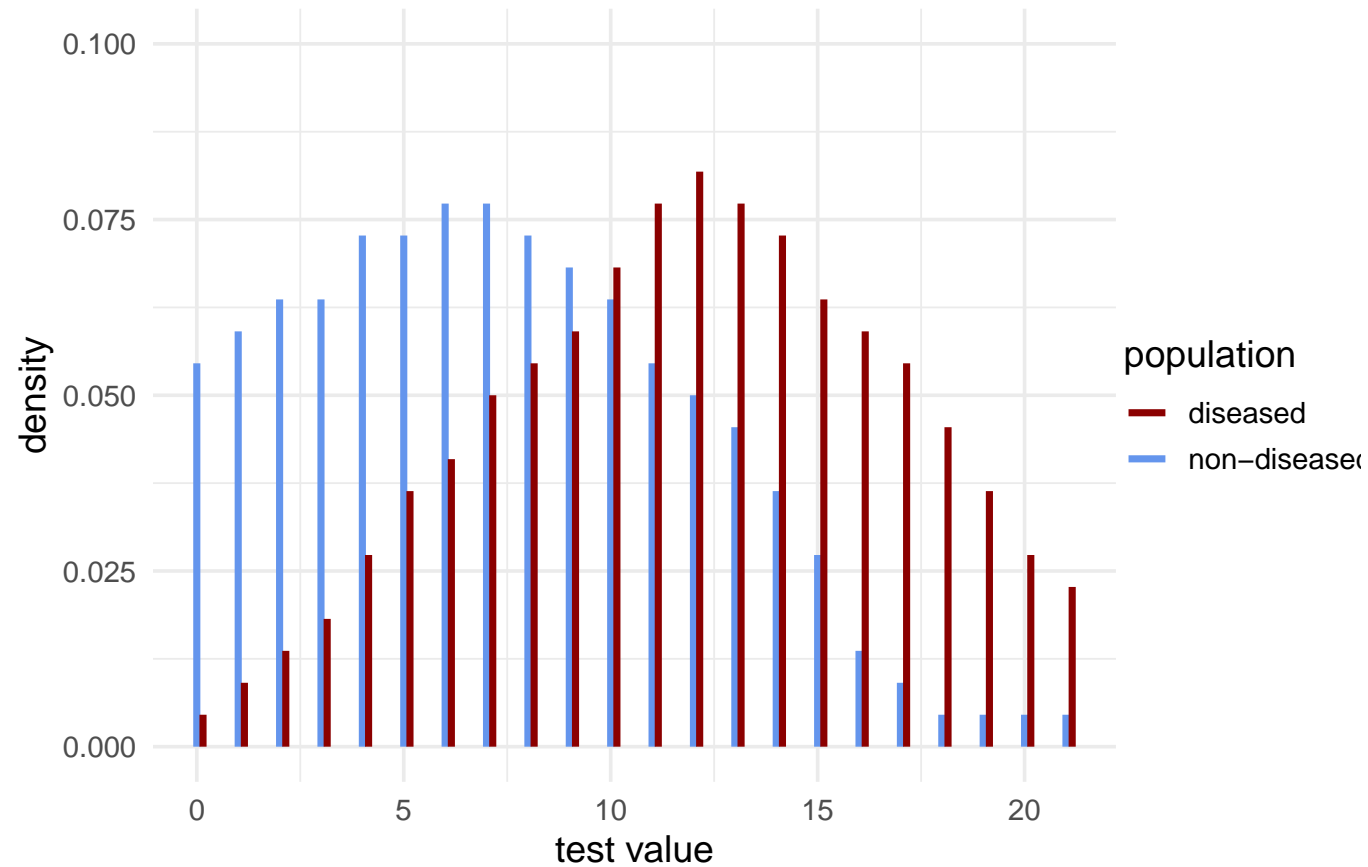

AUC = 0.90

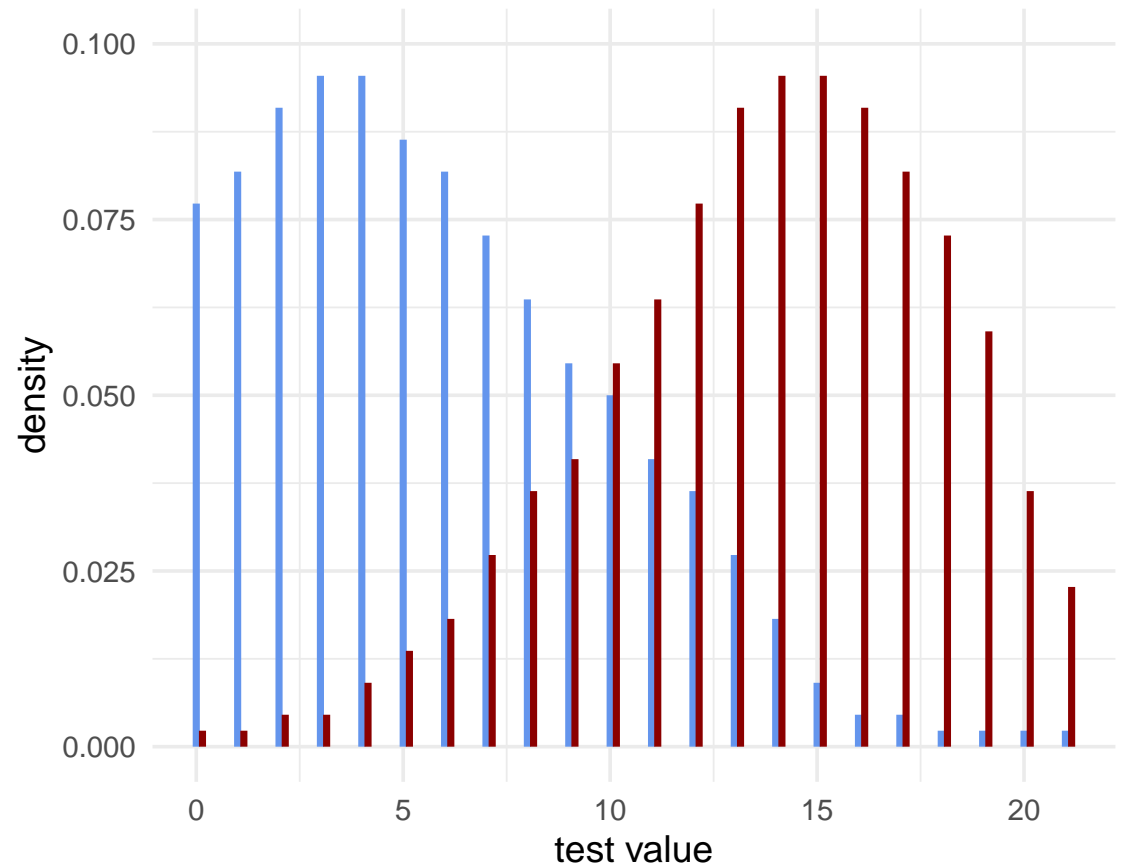

Supplement: Supplementary file 3 — Supporting File 3: bimj70147‐sup‐0003‐simstudy_code.zip. [file BIMJ-68-e70147-s001.zip › figures/Fig_S02.pdf]

AUC RMSE

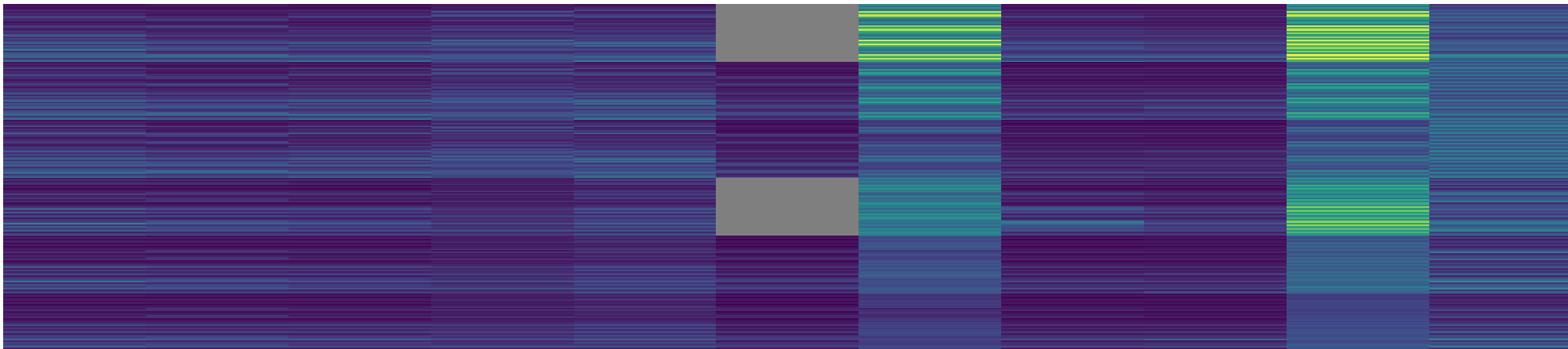

RMSE

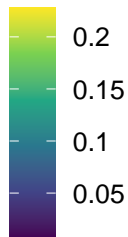

sensitivity RMSE

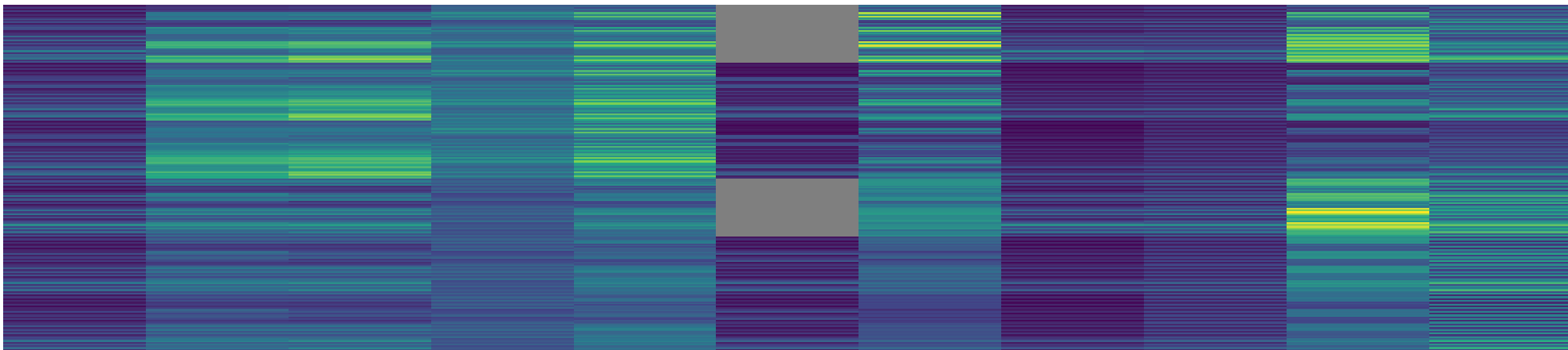

RMSE

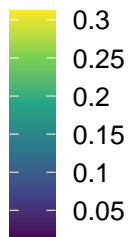

specificity RMSE

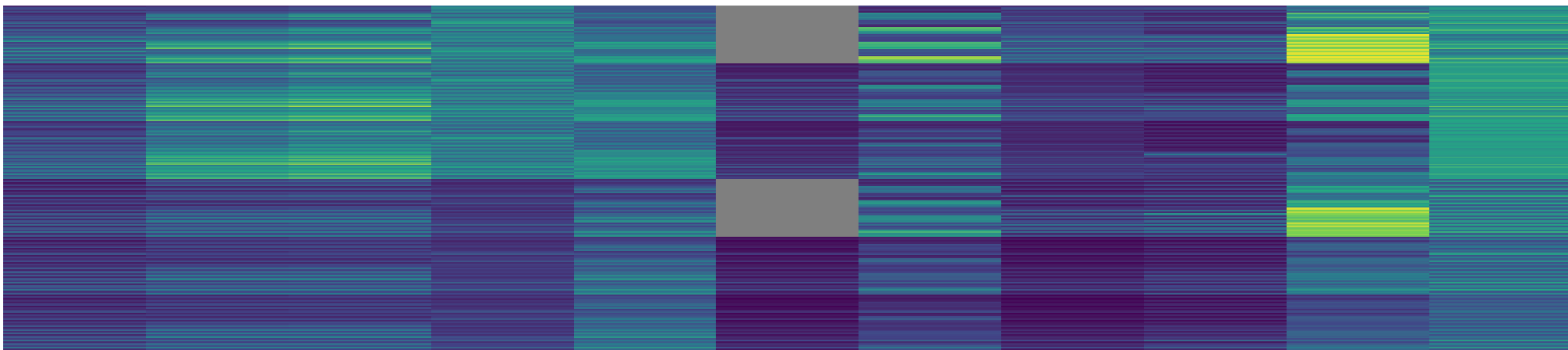

RMSE

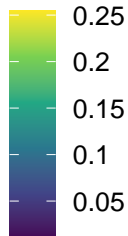

threshold RMSE

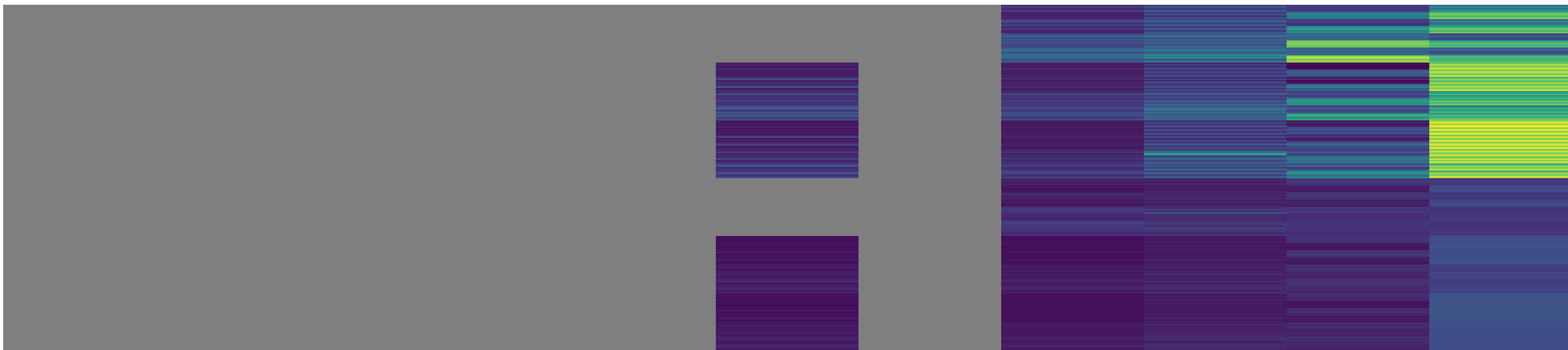

RMSE

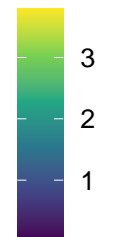

Supplement: Supplementary file 3 — Supporting File 3: bimj70147‐sup‐0003‐simstudy_code.zip. [file BIMJ-68-e70147-s001.zip › figures/Fig_S05_all_heatmaps_rmse.pdf]

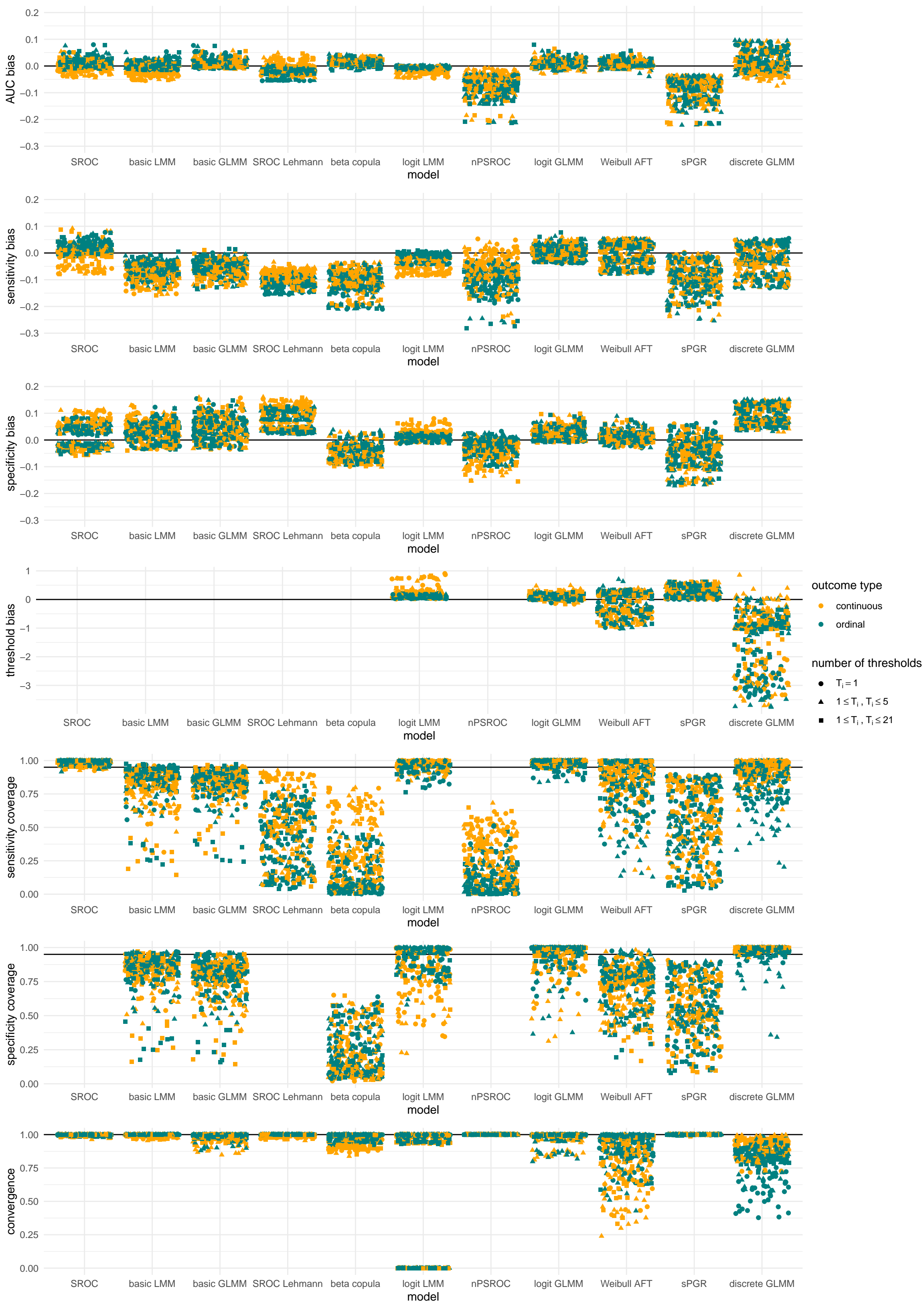

Supplement: Supplementary file 3 — Supporting File 3: bimj70147‐sup‐0003‐simstudy_code.zip. [file BIMJ-68-e70147-s001.zip › figures/Fig_S06_scatterplot_all.pdf]

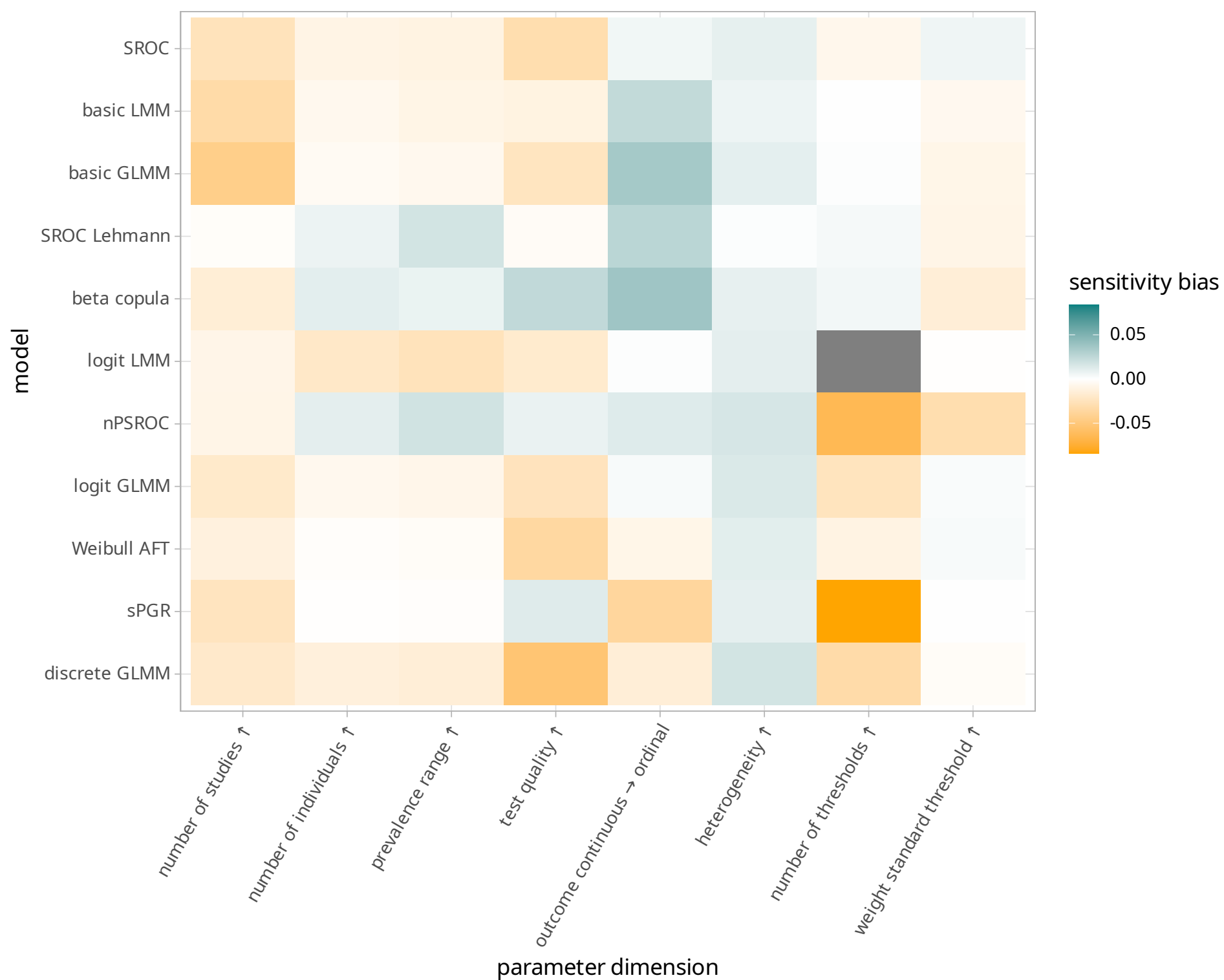

Supplement: Supplementary file 3 — Supporting File 3: bimj70147‐sup‐0003‐simstudy_code.zip. [file BIMJ-68-e70147-s001.zip › figures/Fig_S07_heatmap_sensitivity_bias_effects.pdf]

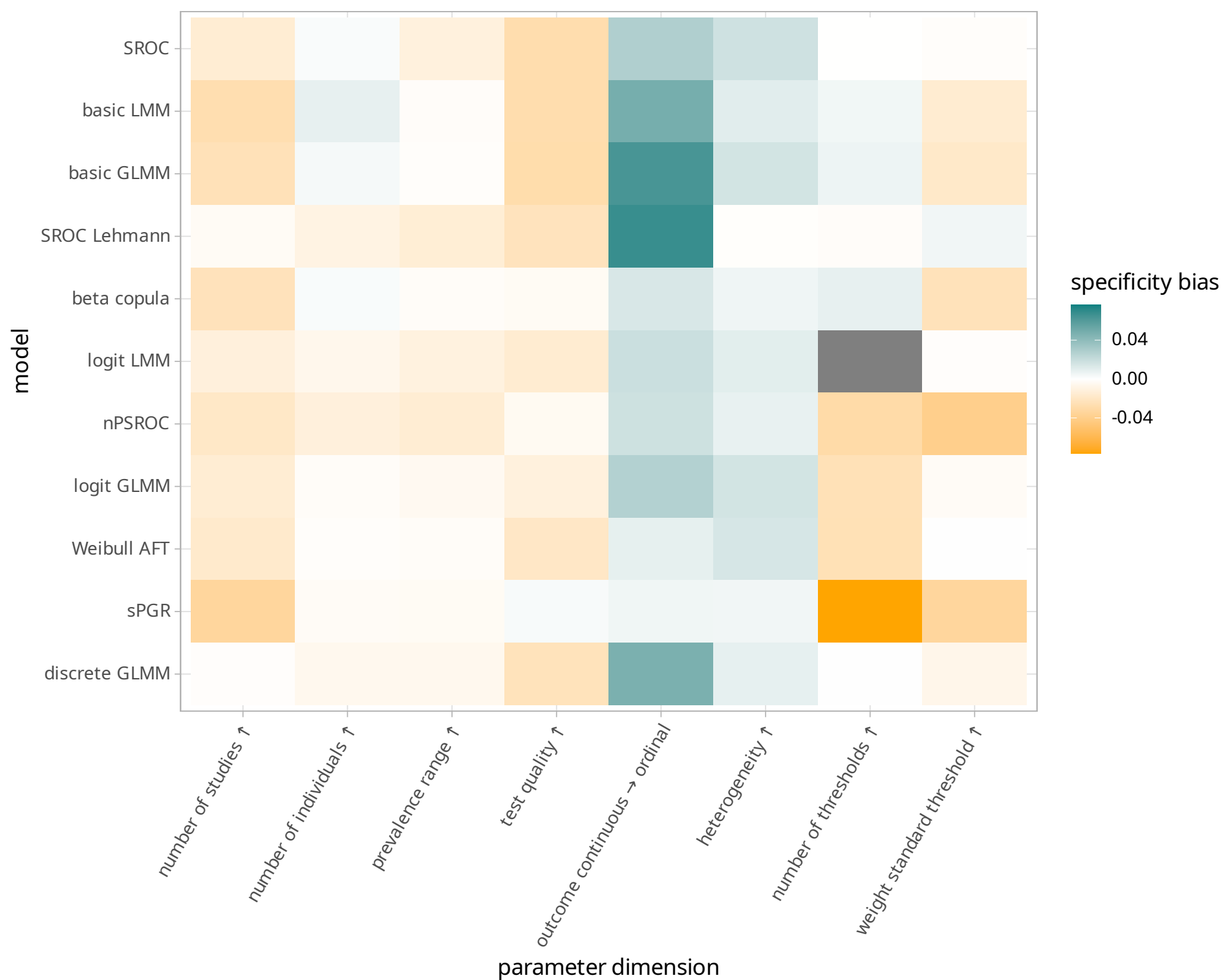

Supplement: Supplementary file 3 — Supporting File 3: bimj70147‐sup‐0003‐simstudy_code.zip. [file BIMJ-68-e70147-s001.zip › figures/Fig_S08_heatmap_specificity_bias_effects.pdf]

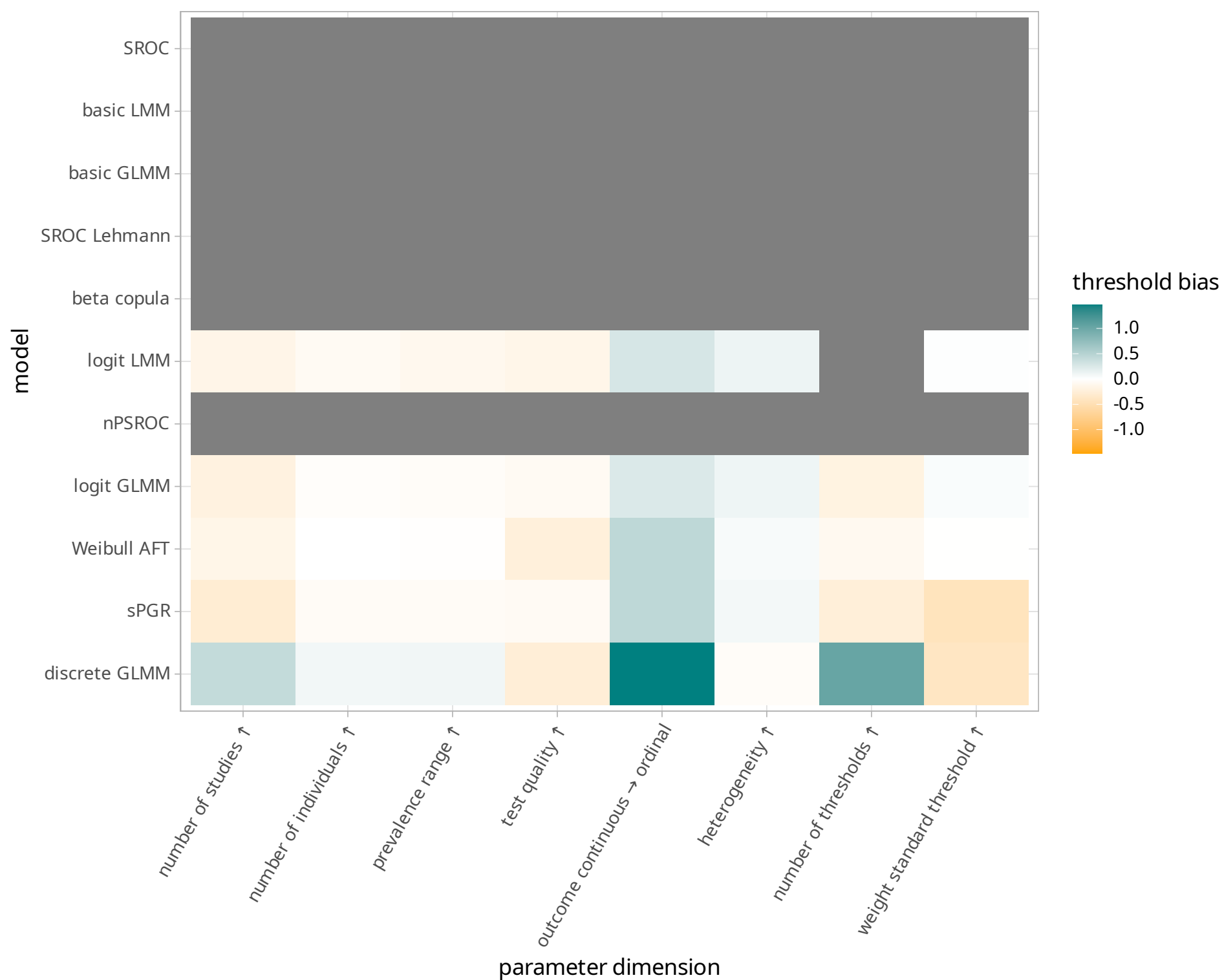

Supplement: Supplementary file 3 — Supporting File 3: bimj70147‐sup‐0003‐simstudy_code.zip. [file BIMJ-68-e70147-s001.zip › figures/Fig_S09_heatmap_threshold_bias_effects.pdf]

model

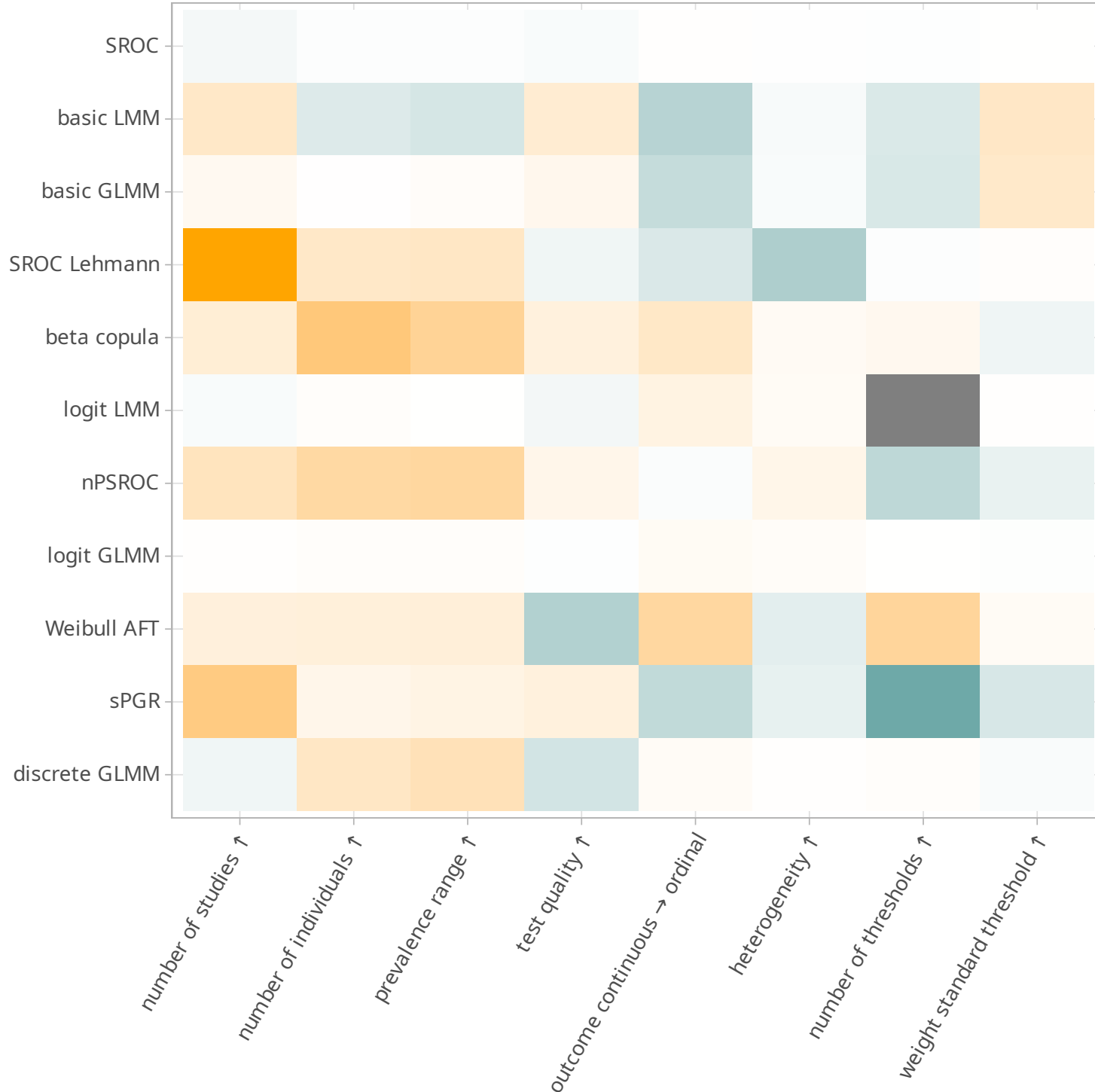

sensitivity coverage

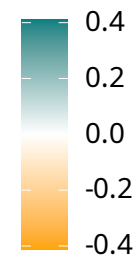

parameter dimension

Supplement: Supplementary file 3 — Supporting File 3: bimj70147‐sup‐0003‐simstudy_code.zip. [file BIMJ-68-e70147-s001.zip › figures/Fig_S10_heatmap_sensitivity_coverage_effects.pdf]

model

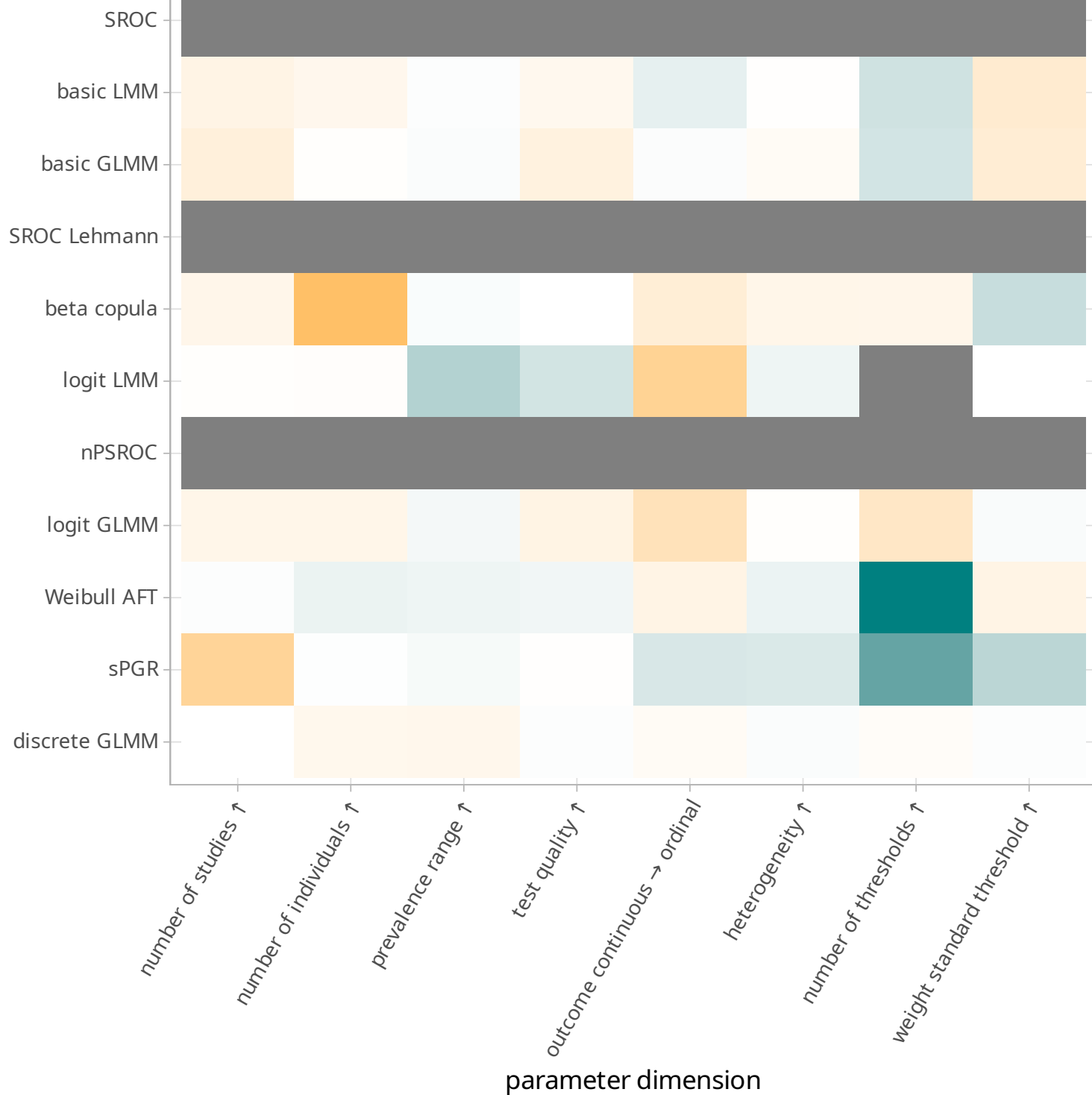

specificity coverage

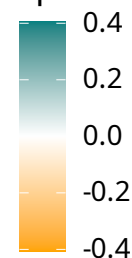

Supplement: Supplementary file 3 — Supporting File 3: bimj70147‐sup‐0003‐simstudy_code.zip. [file BIMJ-68-e70147-s001.zip › figures/Fig_S11_heatmap_specificity_coverage_effects.pdf]

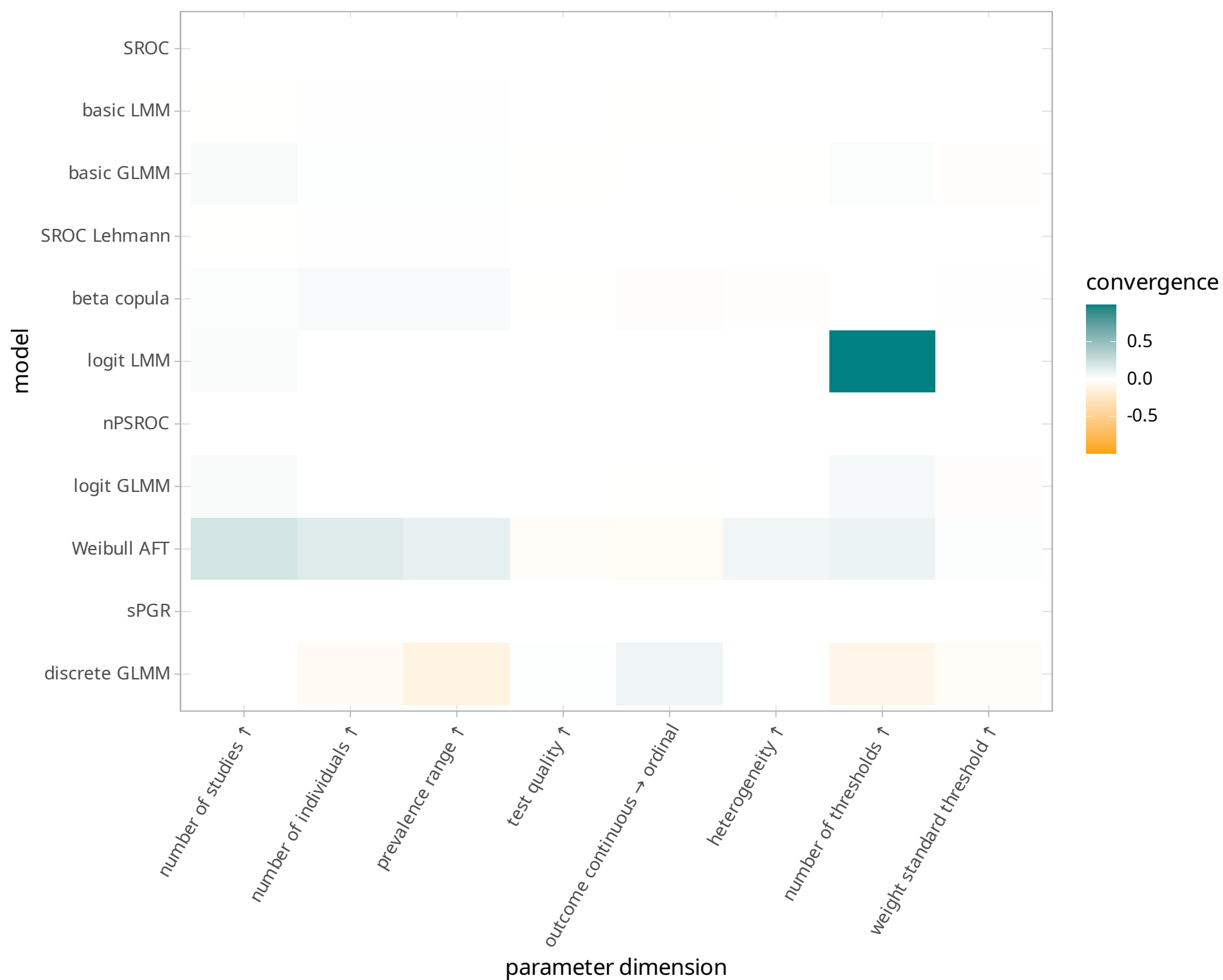

Supplement: Supplementary file 3 — Supporting File 3: bimj70147‐sup‐0003‐simstudy_code.zip. [file BIMJ-68-e70147-s001.zip › figures/Fig_S12_heatmap_convergence_effects.pdf]

AUC bias

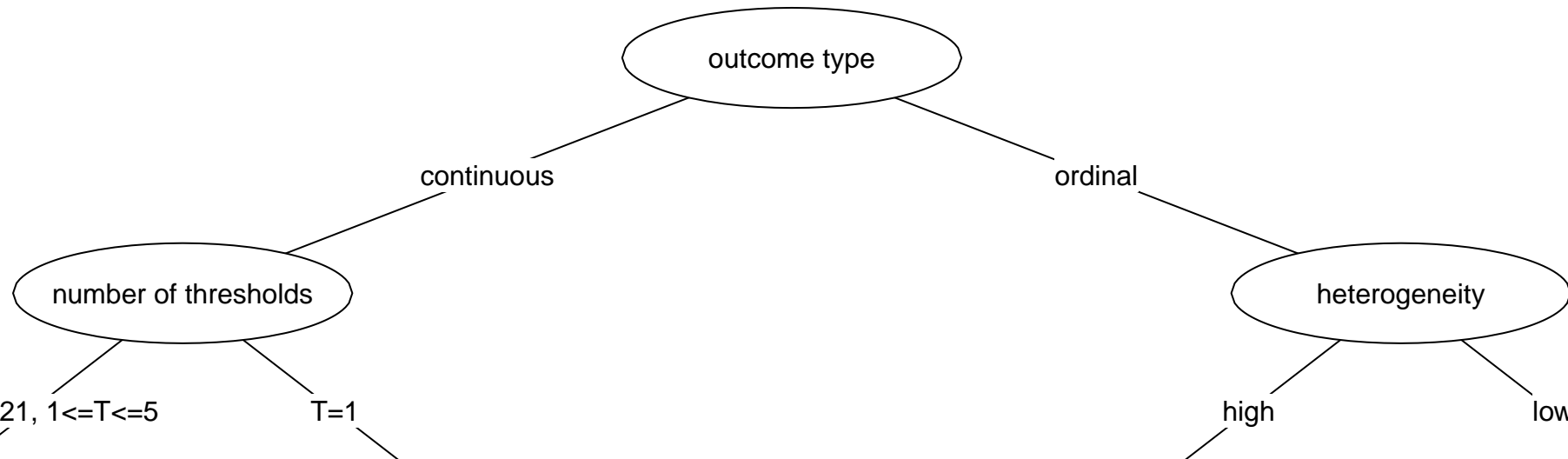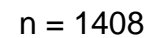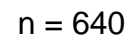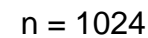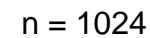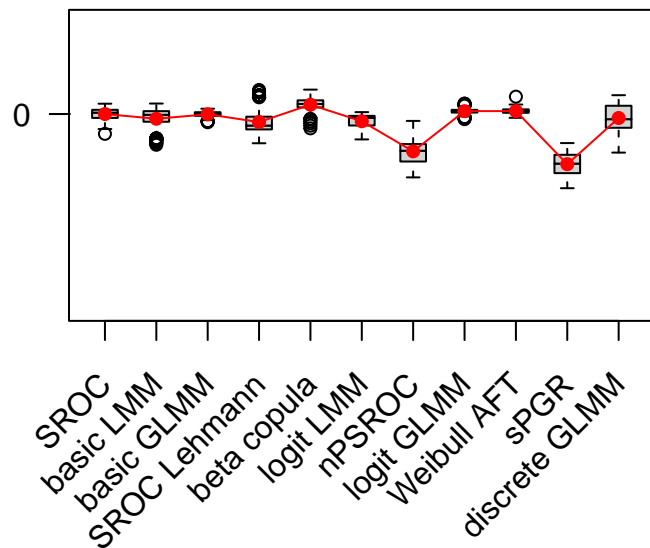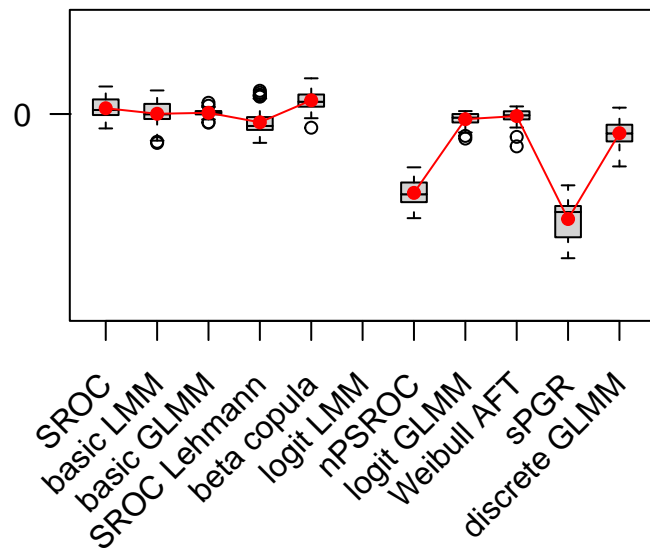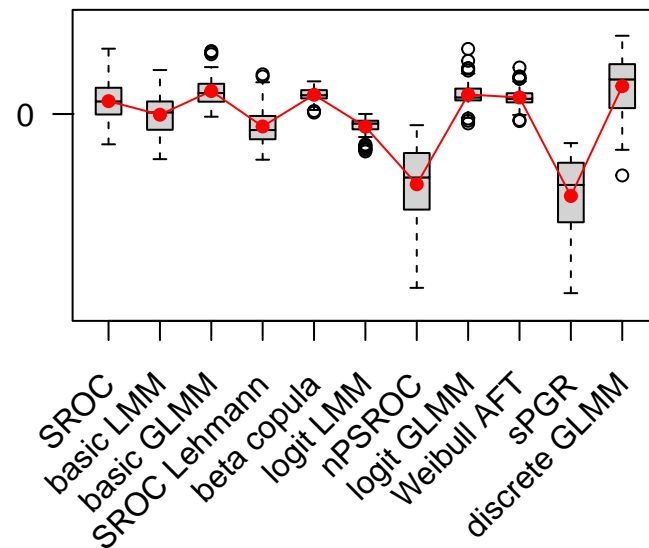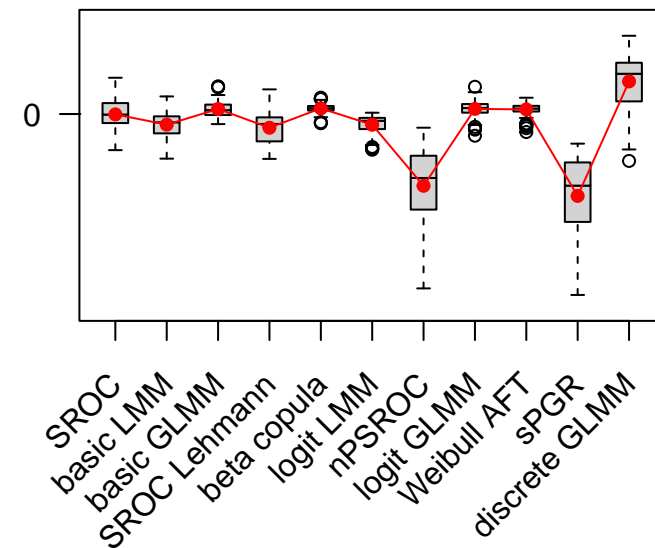

Supplement: Supplementary file 3 — Supporting File 3: bimj70147‐sup‐0003‐simstudy_code.zip. [file BIMJ-68-e70147-s001.zip › figures/Fig_S13_mobaucbias.pdf]

mean coverage sensitivity

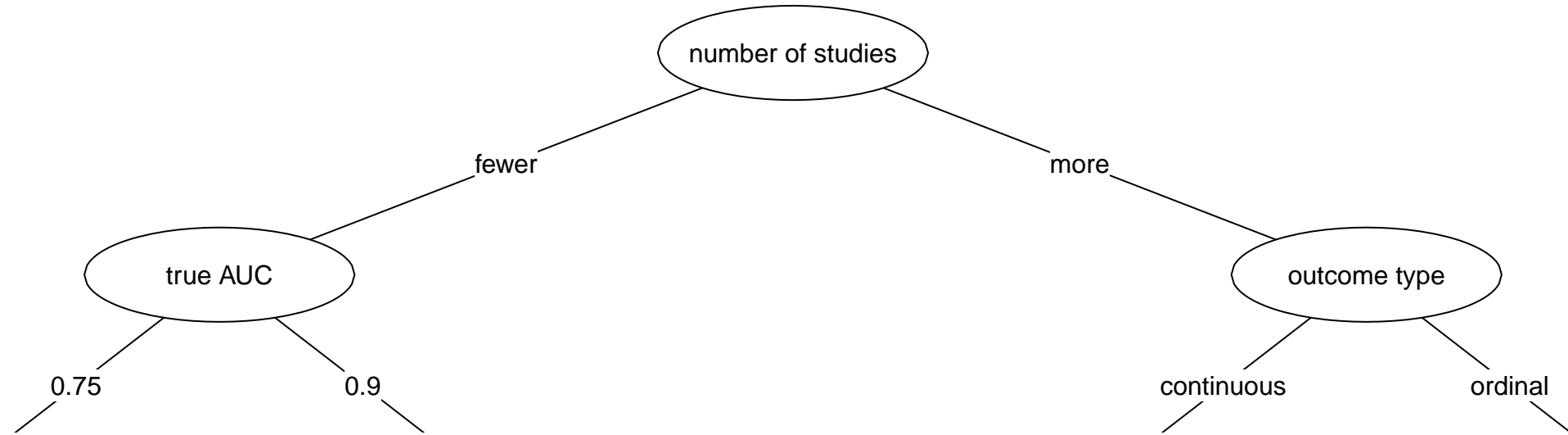

n = 1024

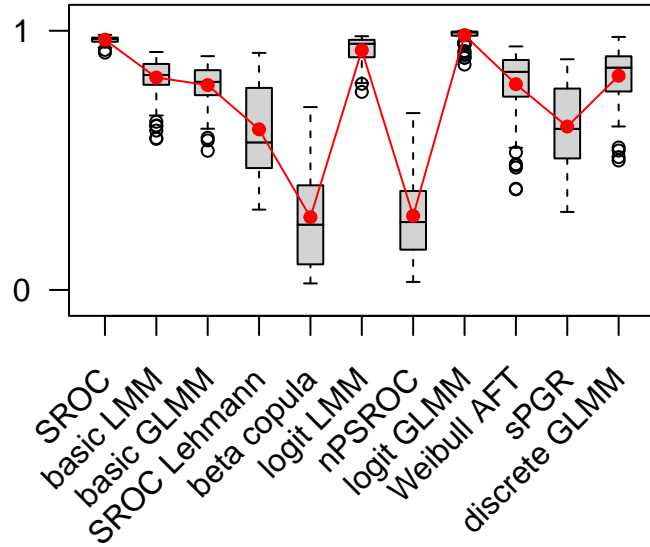

n = 1024

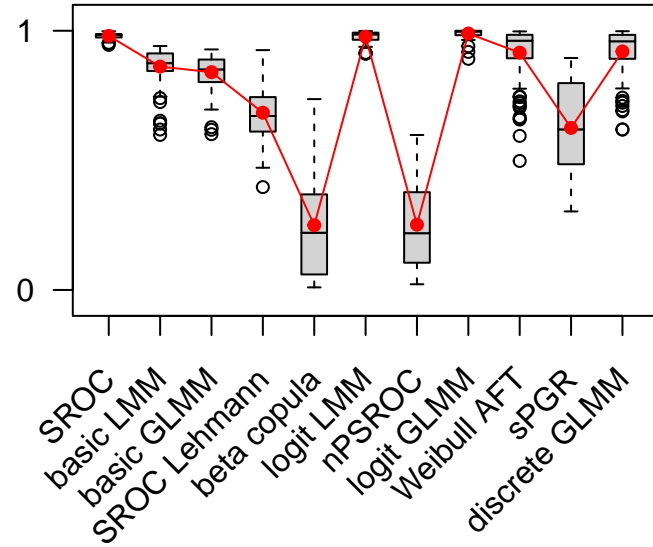

n = 1024

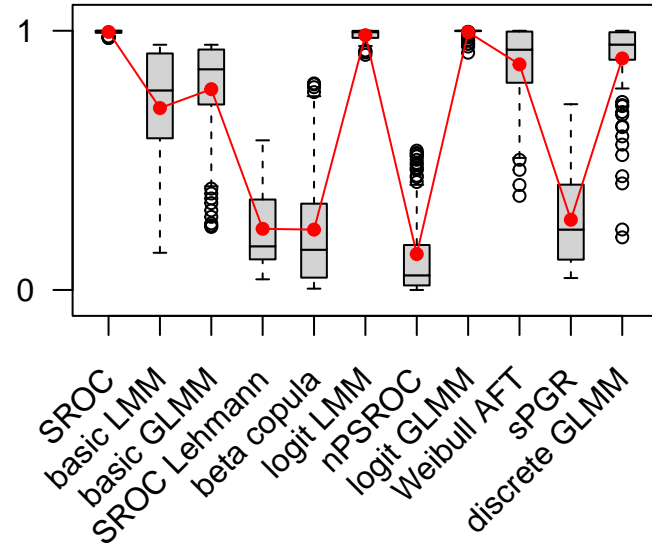

n = 1024

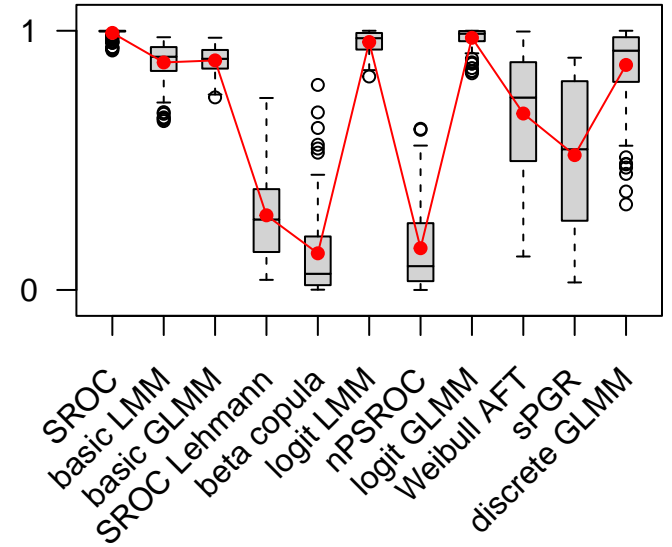

Supplement: Supplementary file 3 — Supporting File 3: bimj70147‐sup‐0003‐simstudy_code.zip. [file BIMJ-68-e70147-s001.zip › figures/Fig_S14_mobsenscov.pdf]

mean coverage specificity

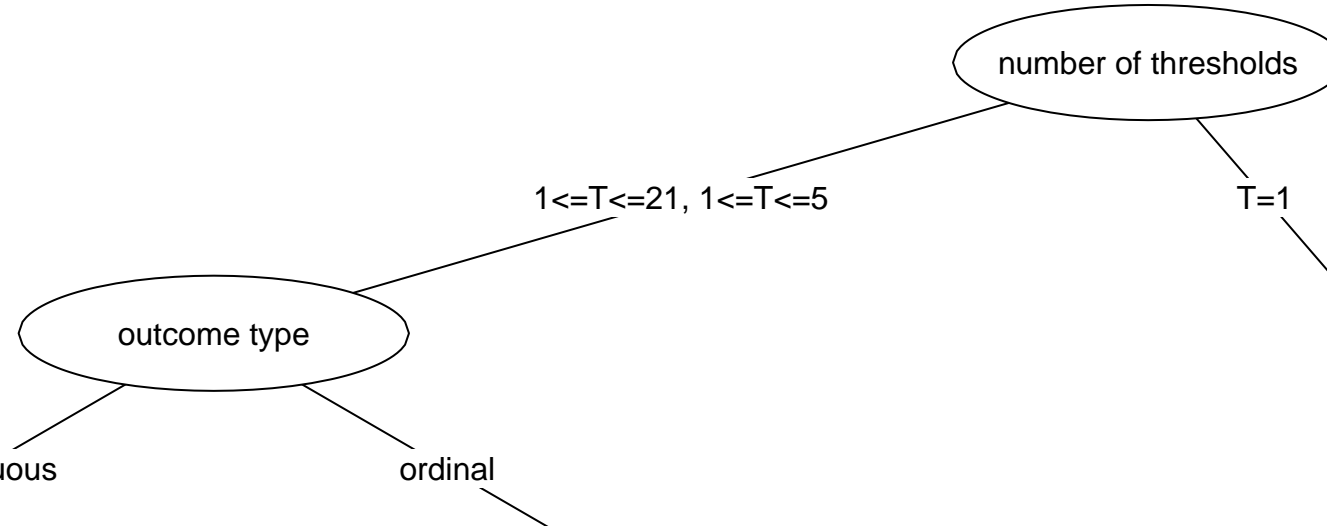

n = 1024

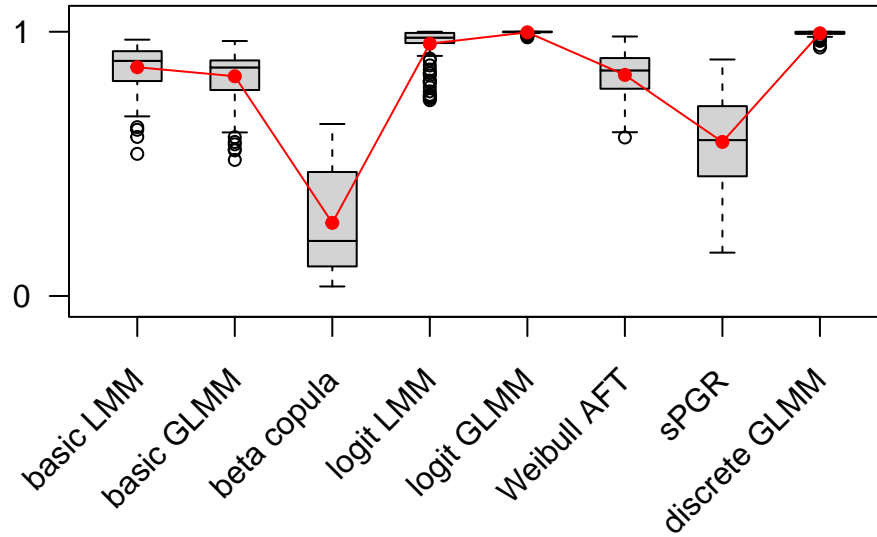

n = 1024

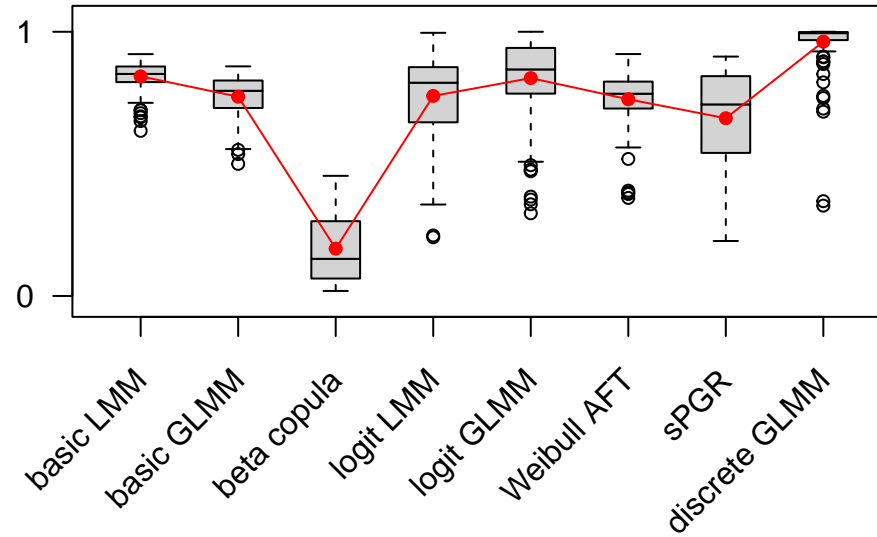

n = 896

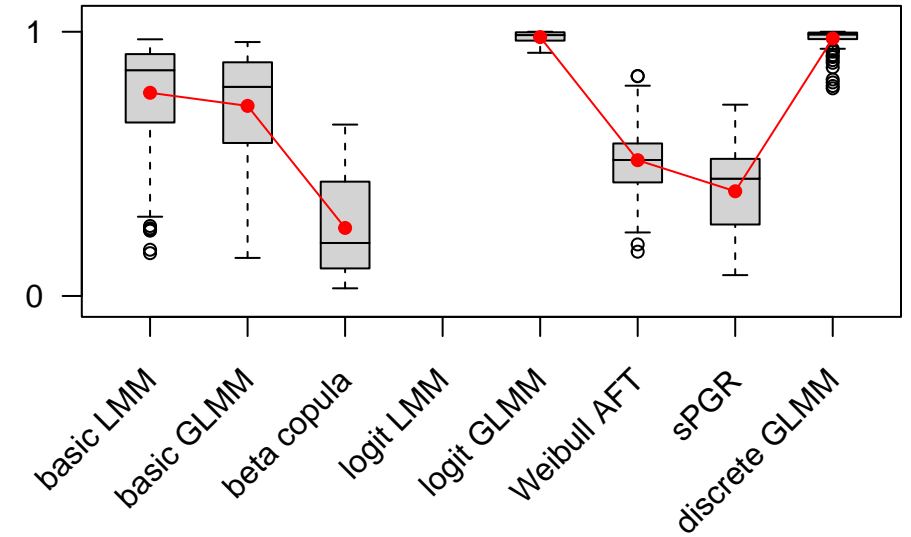

Supplement: Supplementary file 3 — Supporting File 3: bimj70147‐sup‐0003‐simstudy_code.zip. [file BIMJ-68-e70147-s001.zip › figures/Fig_S14_mobspeccov.pdf]

# threshold bias

outcome type

continuous

ordinal

number of thresholds

$1 \leq T \leq 21$ ,  $1 \leq T \leq 5$

$T=1$

n = 896

n = 640

n = 256

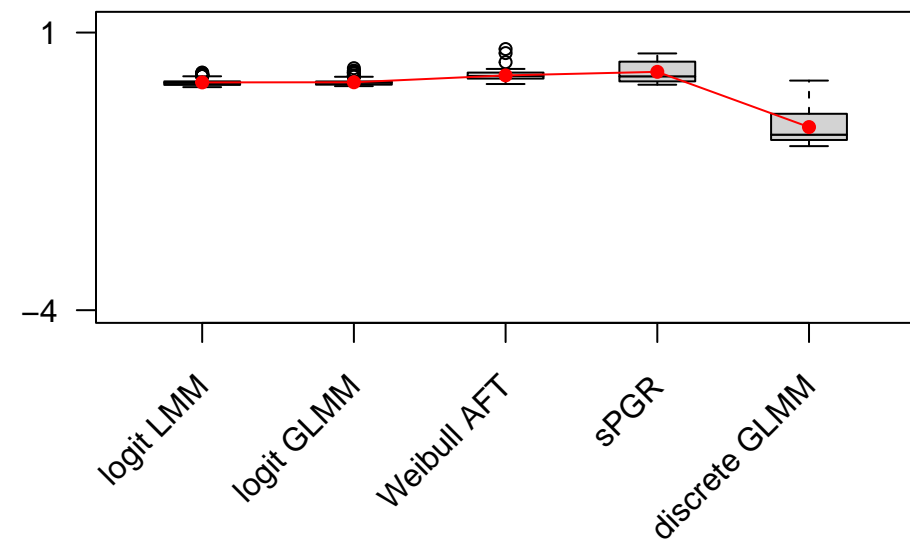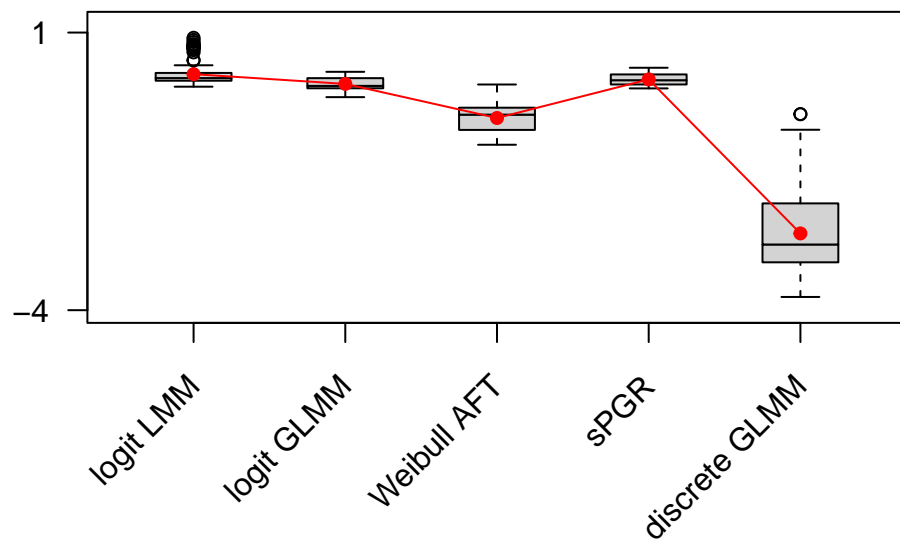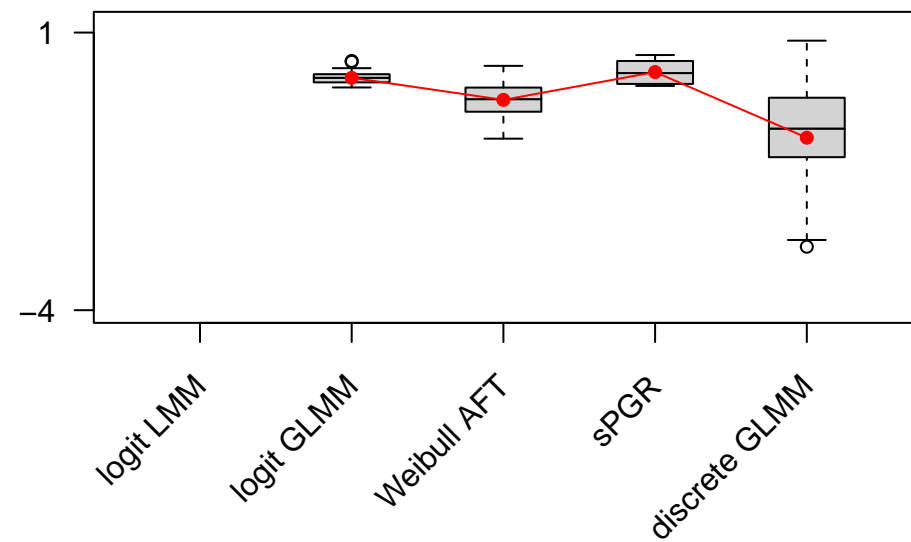

Supplement: Supplementary file 3 — Supporting File 3: bimj70147‐sup‐0003‐simstudy_code.zip. [file BIMJ-68-e70147-s001.zip › figures/Fig_S14_mobtrbias.pdf]

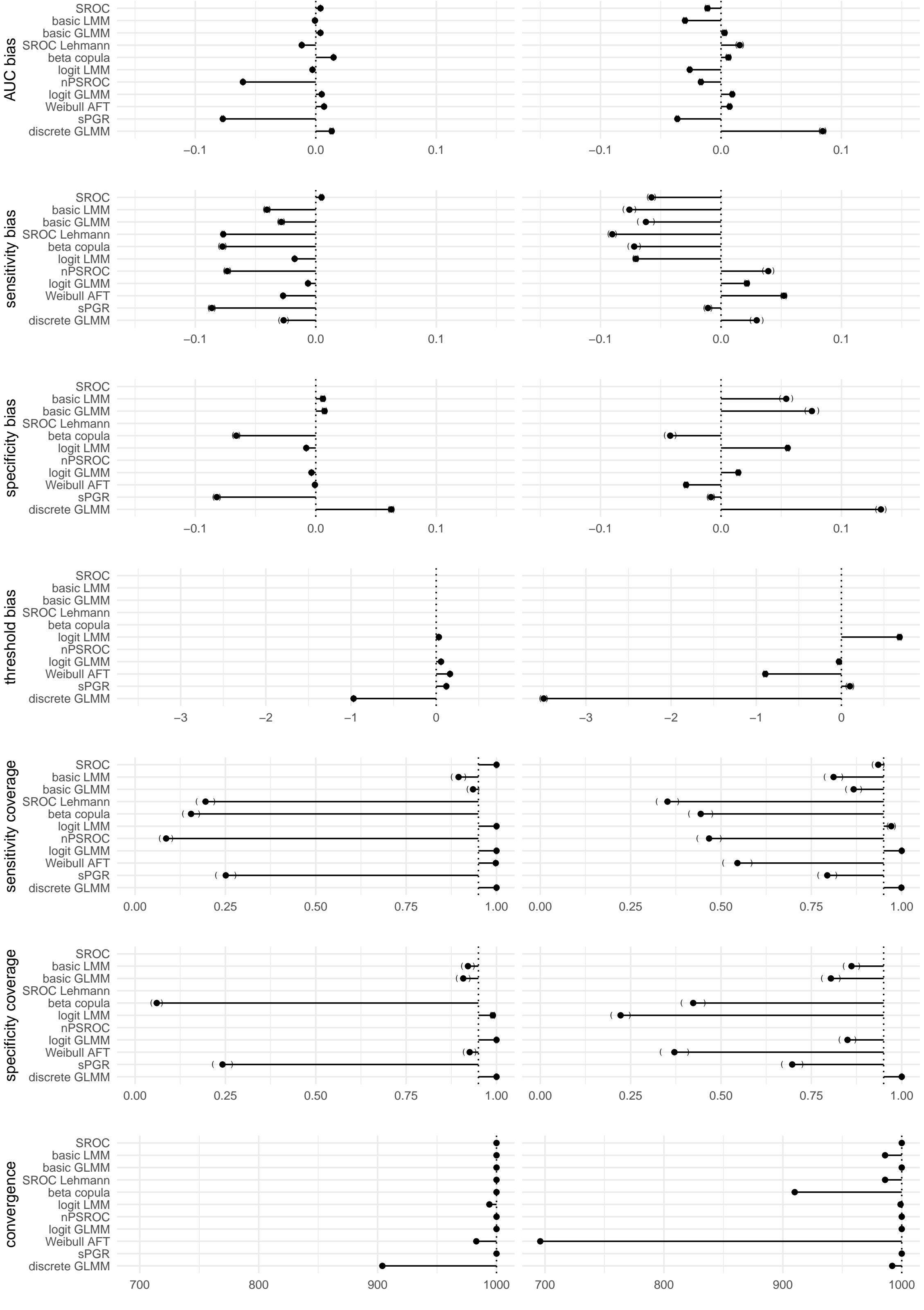

Supplement: Supplementary file 3 — Supporting File 3: bimj70147‐sup‐0003‐simstudy_code.zip. [file BIMJ-68-e70147-s001.zip › figures/Fig_S15_lollipops_22211211_and_21321112.pdf]

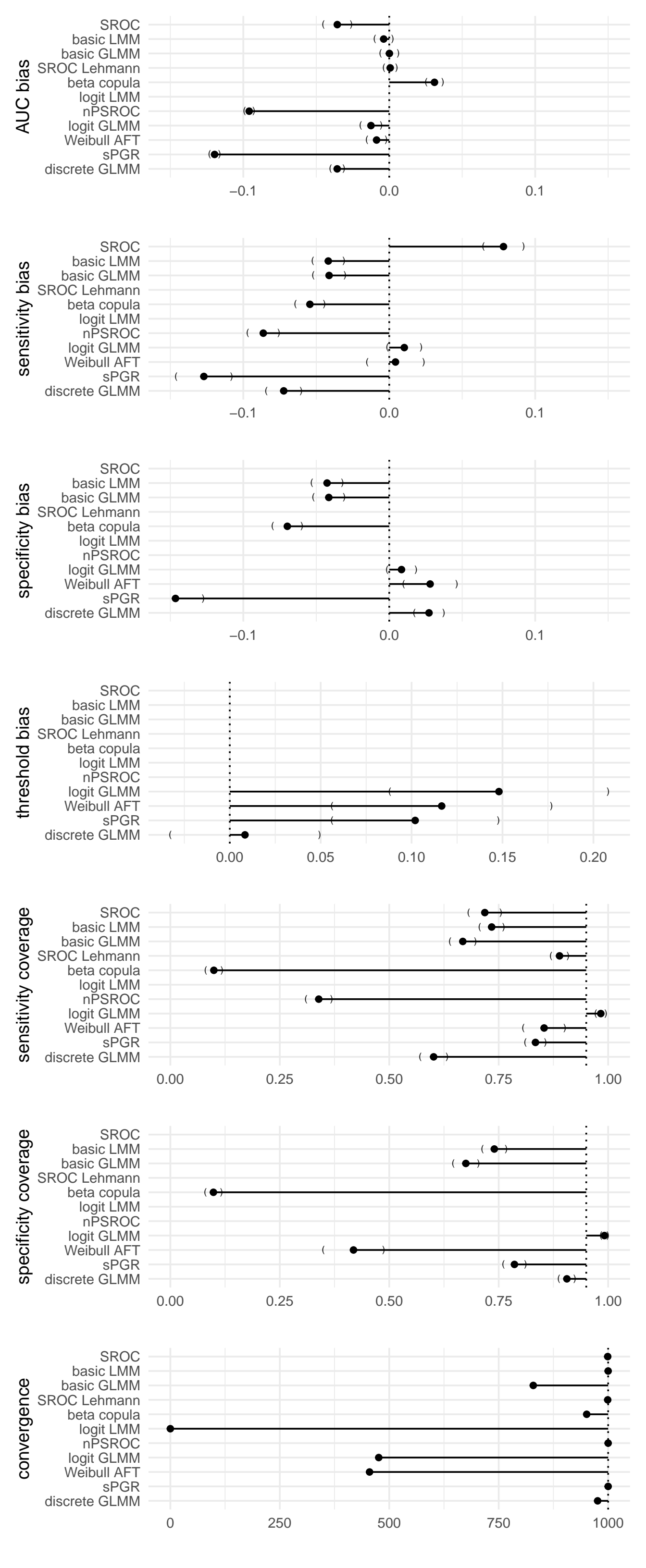

Supplement: Supplementary file 3 — Supporting File 3: bimj70147‐sup‐0003‐simstudy_code.zip. [file BIMJ-68-e70147-s001.zip › figures/Fig_S16_lollipops_2to3.pdf]

AUC bias

weight standard threshold

0

0.7

n = 960

n = 960

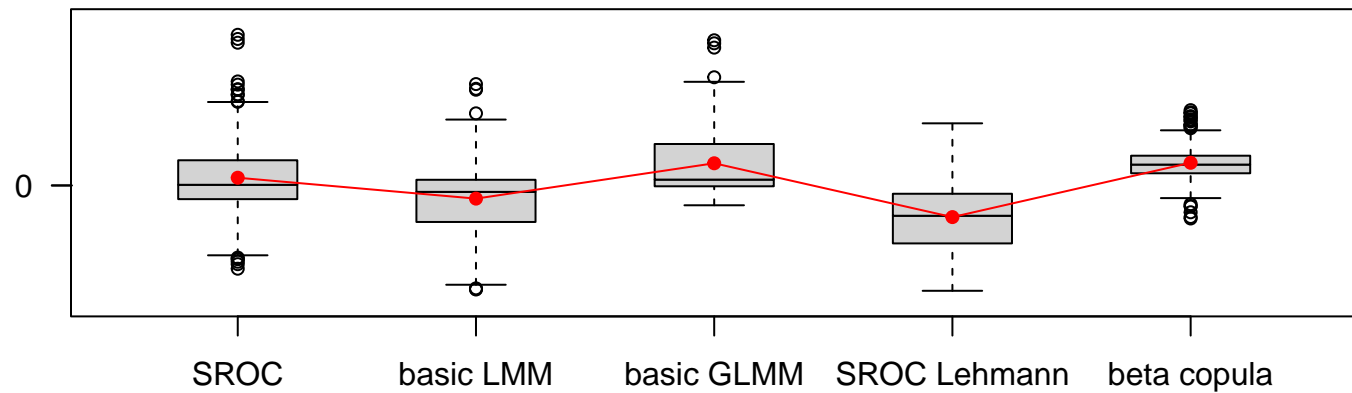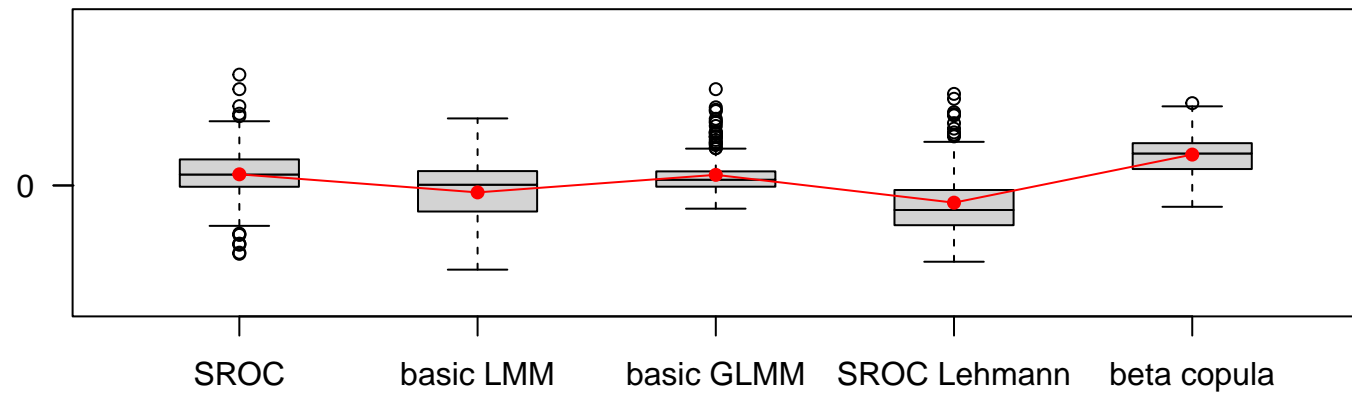

Supplement: Supplementary file 3 — Supporting File 3: bimj70147‐sup‐0003‐simstudy_code.zip. [file BIMJ-68-e70147-s001.zip › figures/Fig_S17_moball1.pdf]

sensitivity bias

weight standard threshold

0

0.7

n = 960

n = 960

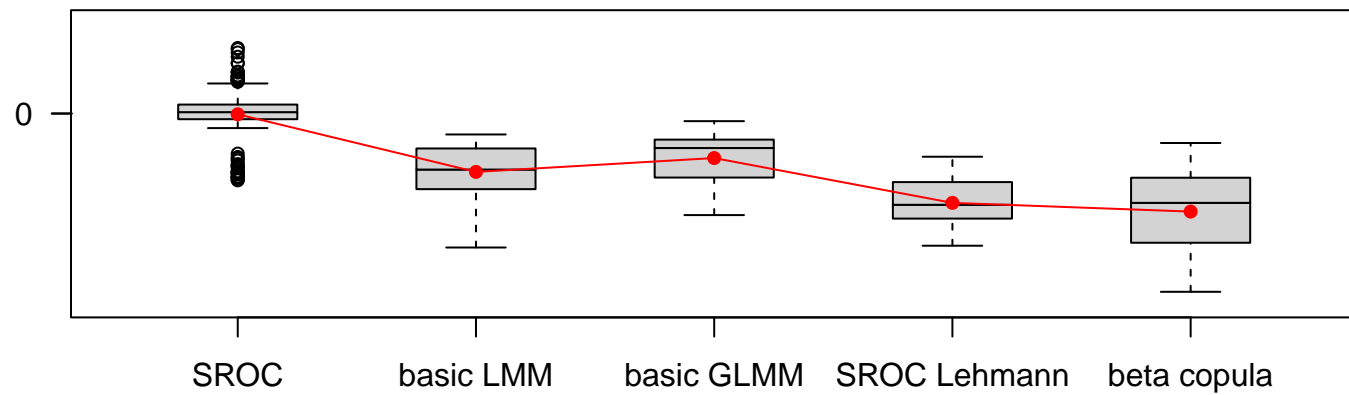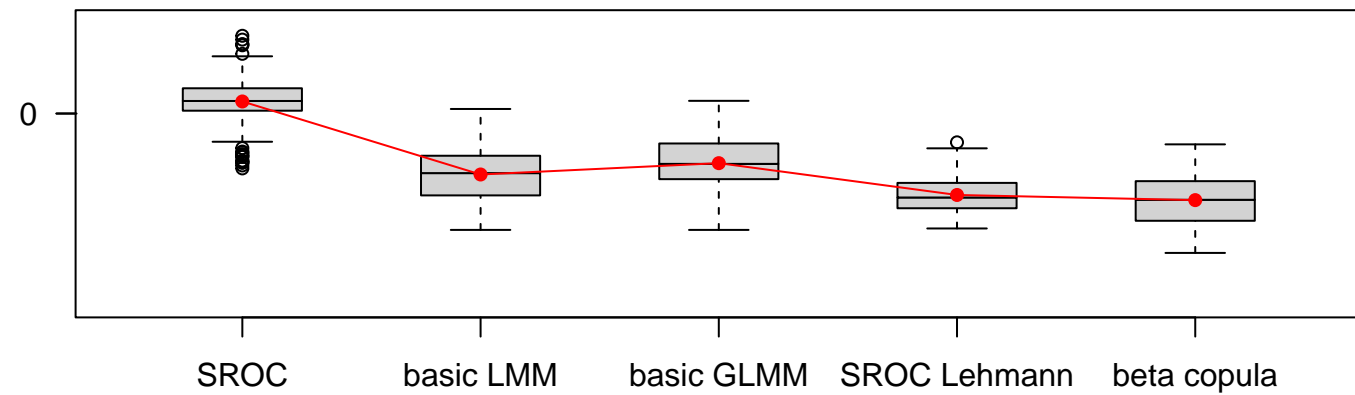

Supplement: Supplementary file 3 — Supporting File 3: bimj70147‐sup‐0003‐simstudy_code.zip. [file BIMJ-68-e70147-s001.zip › figures/Fig_S17_moball2.pdf]

specificity bias

weight standard threshold

0

0.7

n = 960

n = 960

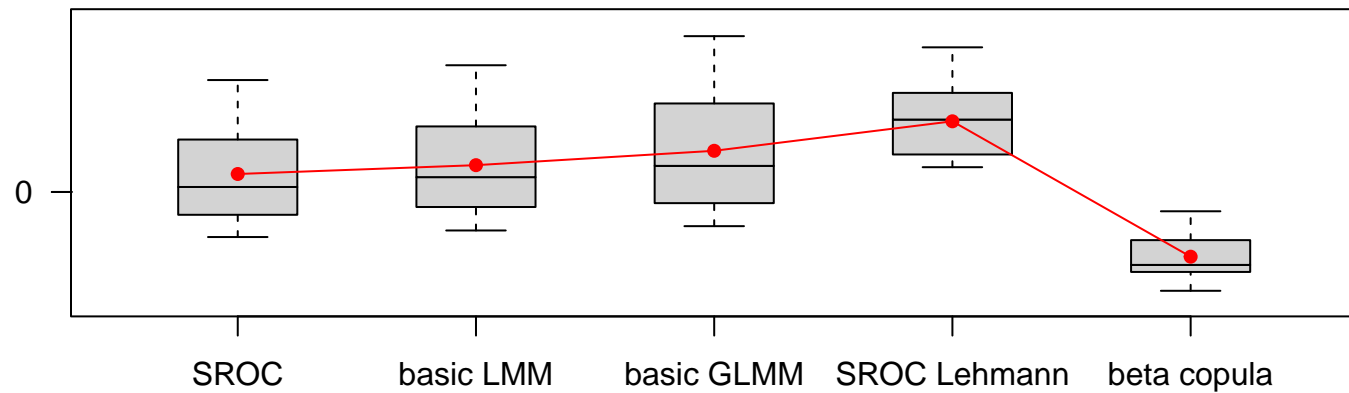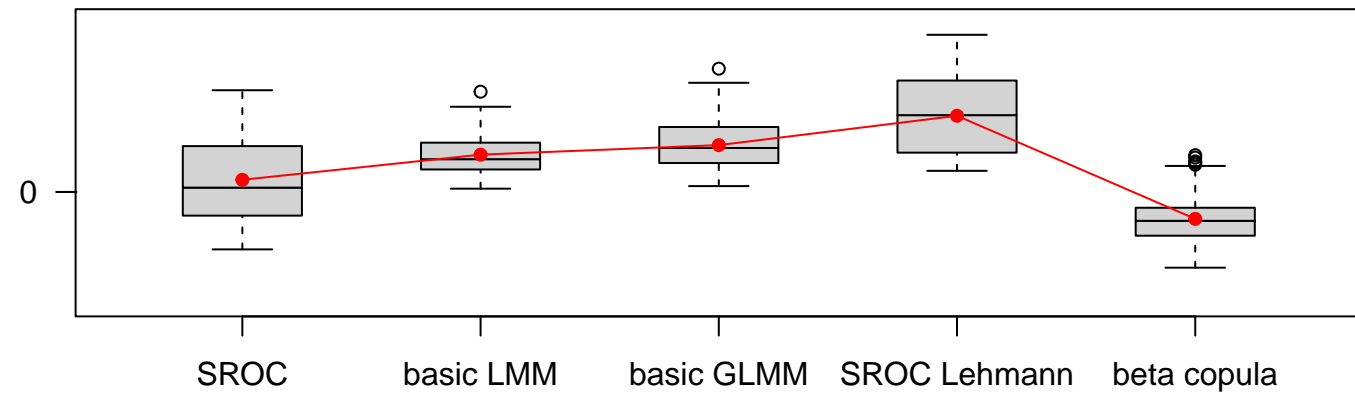

Supplement: Supplementary file 3 — Supporting File 3: bimj70147‐sup‐0003‐simstudy_code.zip. [file BIMJ-68-e70147-s001.zip › figures/Fig_S17_moball3.pdf]

sensitivity coverage

weight standard threshold

0

0.7

n = 960

n = 960

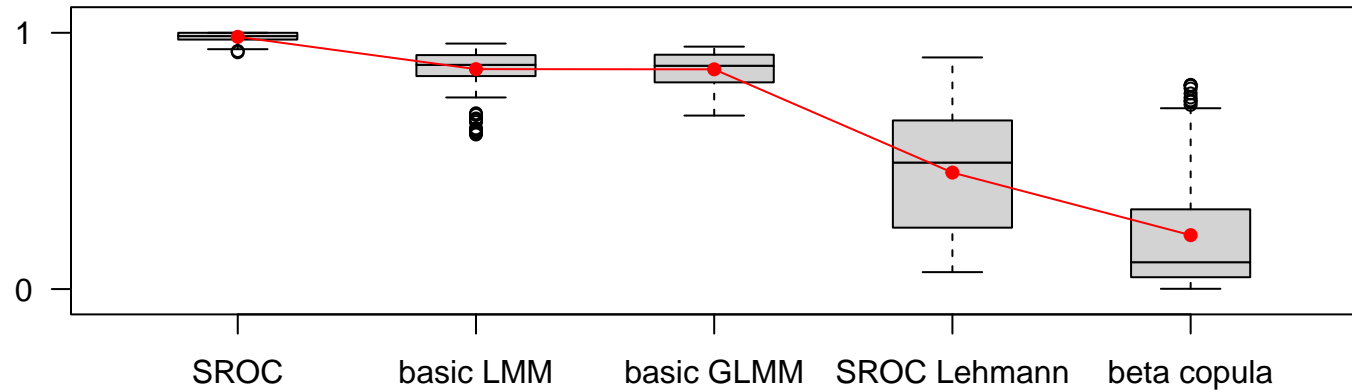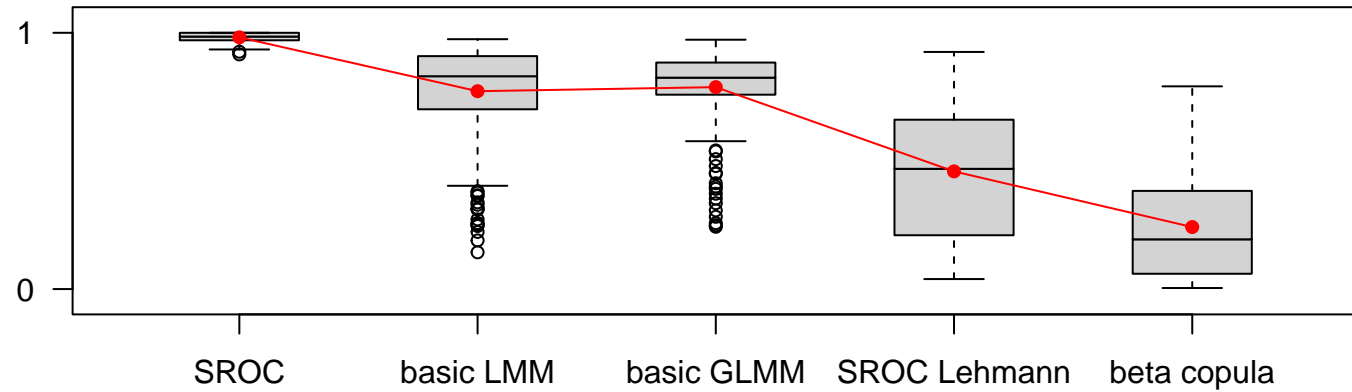

Supplement: Supplementary file 3 — Supporting File 3: bimj70147‐sup‐0003‐simstudy_code.zip. [file BIMJ-68-e70147-s001.zip › figures/Fig_S17_moball4.pdf]

specificity coverage

weight standard threshold

0

0.7

n = 576

n = 576

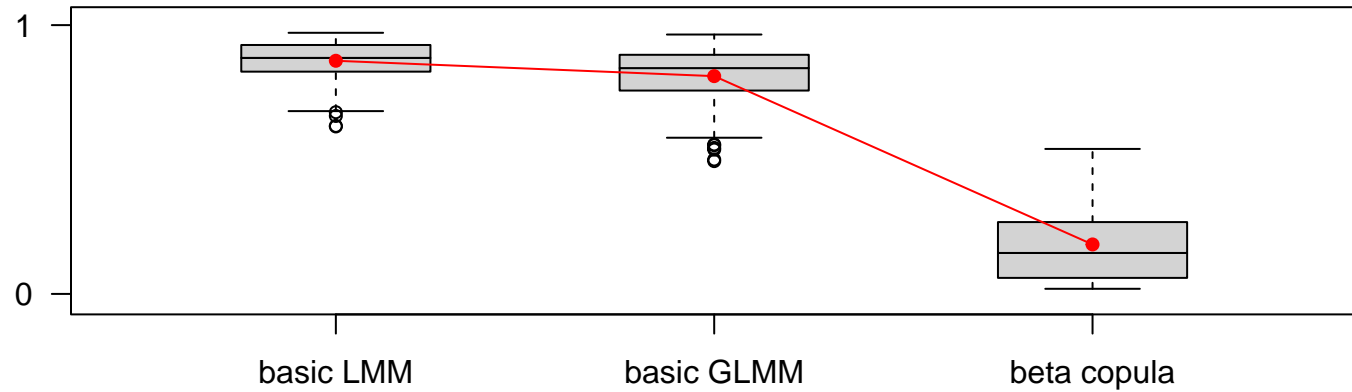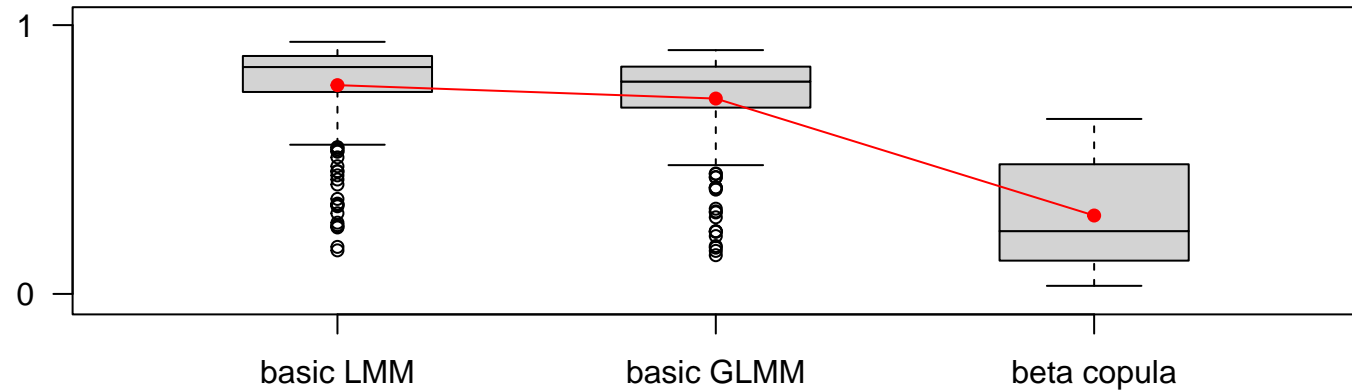

Supplement: Supplementary file 3 — Supporting File 3: bimj70147‐sup‐0003‐simstudy_code.zip. [file BIMJ-68-e70147-s001.zip › figures/Fig_S17_moball5.pdf]

AUC bias

weight standard threshold

0

0.7

n = 320

n = 320

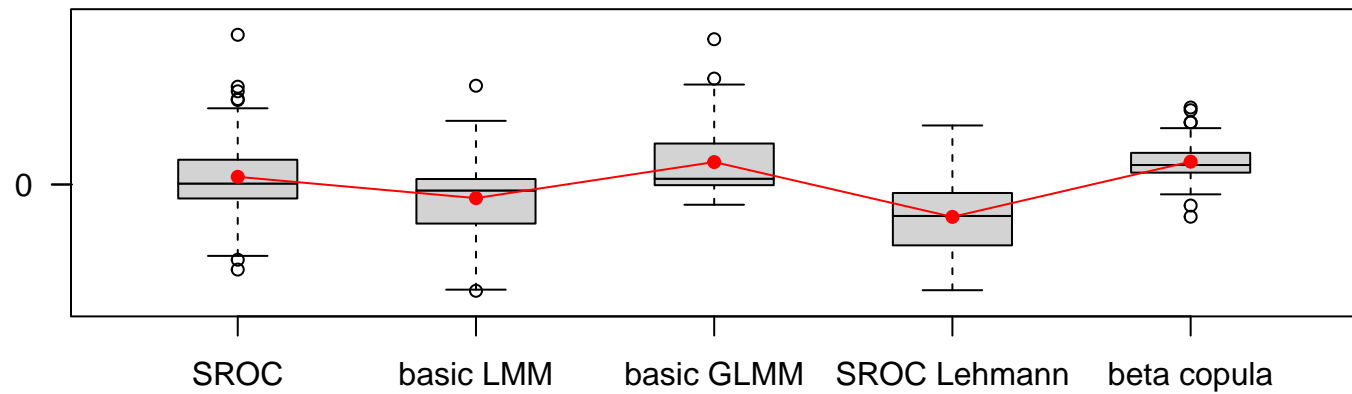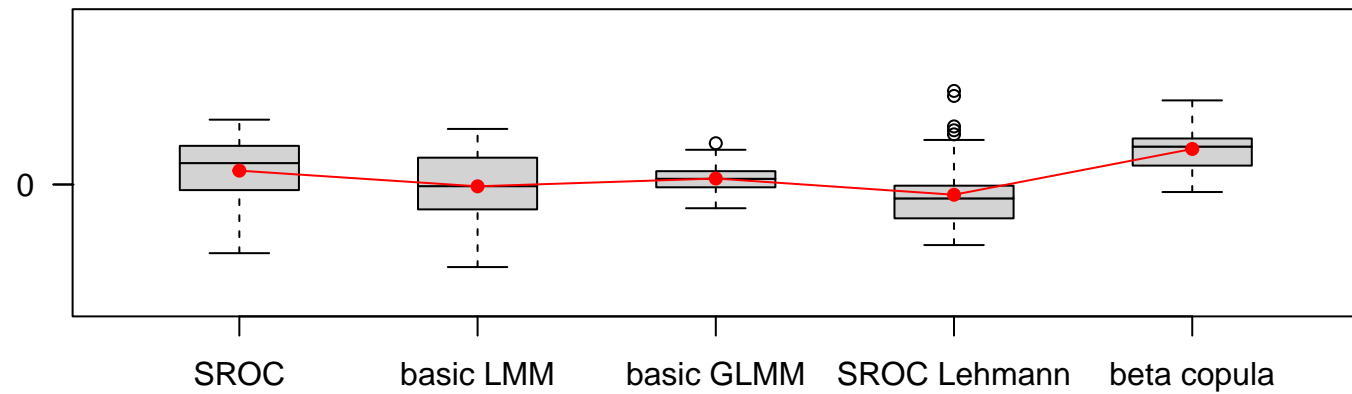

Supplement: Supplementary file 3 — Supporting File 3: bimj70147‐sup‐0003‐simstudy_code.zip. [file BIMJ-68-e70147-s001.zip › figures/Fig_S18_mob1t1.pdf]

sensitivity bias

weight standard threshold

0

0.7

n = 320

n = 320

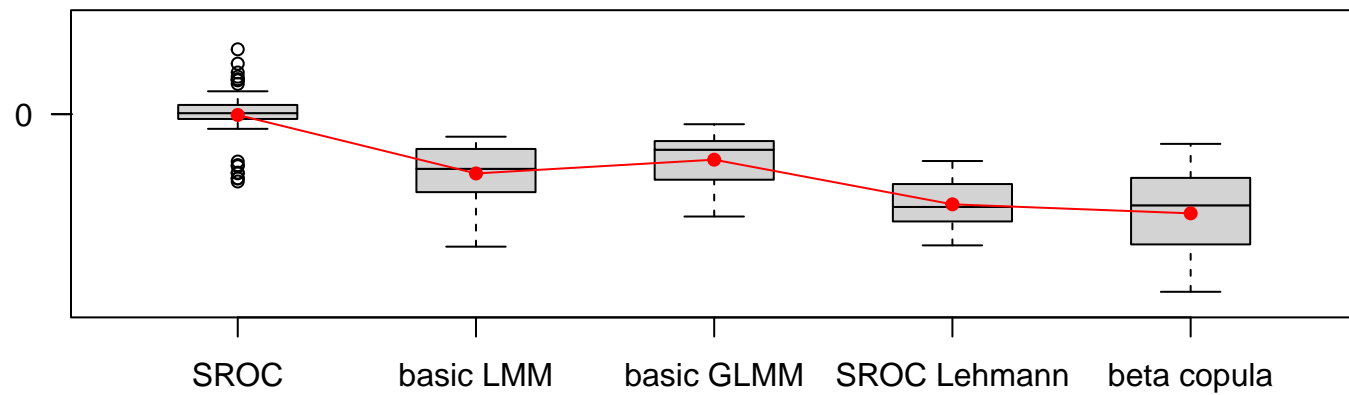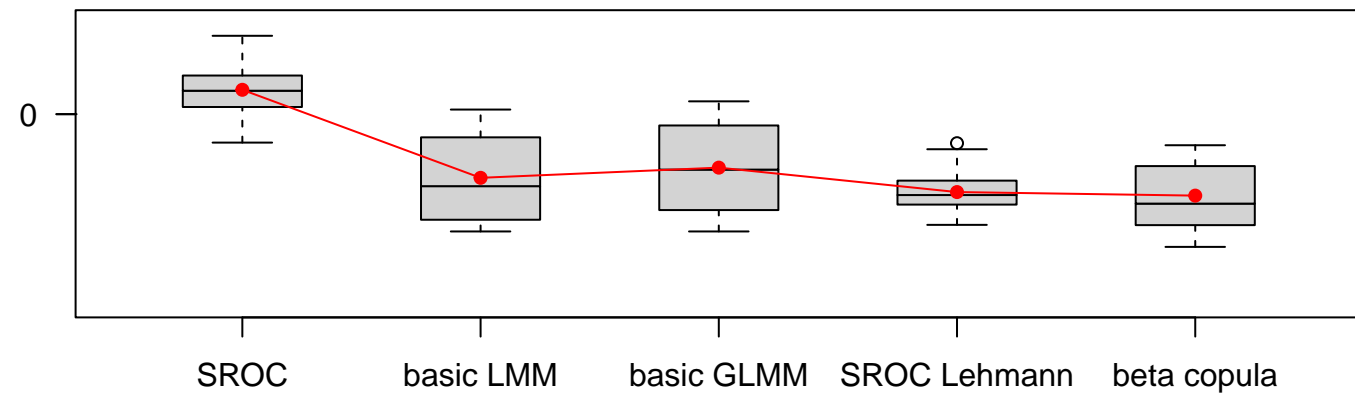

Supplement: Supplementary file 3 — Supporting File 3: bimj70147‐sup‐0003‐simstudy_code.zip. [file BIMJ-68-e70147-s001.zip › figures/Fig_S18_mob1t2.pdf]

specificity bias

weight standard threshold

0

0.7

n = 320

n = 320

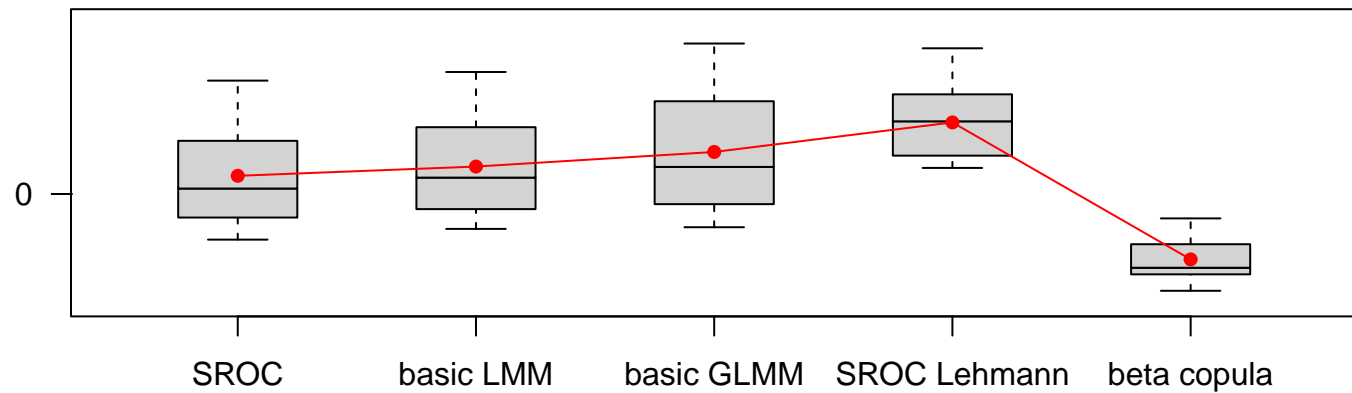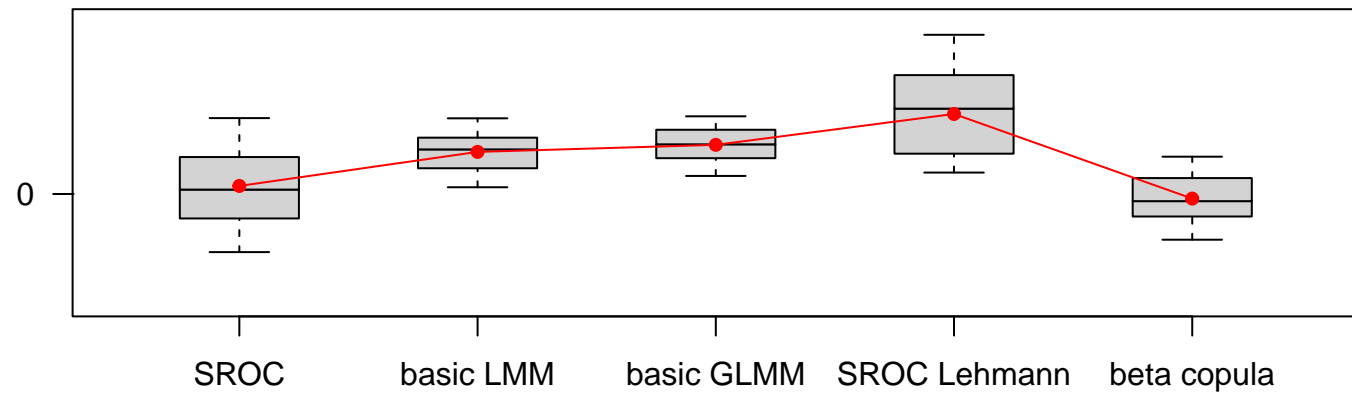

Supplement: Supplementary file 3 — Supporting File 3: bimj70147‐sup‐0003‐simstudy_code.zip. [file BIMJ-68-e70147-s001.zip › figures/Fig_S18_mob1t3.pdf]

sensitivity coverage

weight standard threshold

0

0.7

n = 320

n = 320

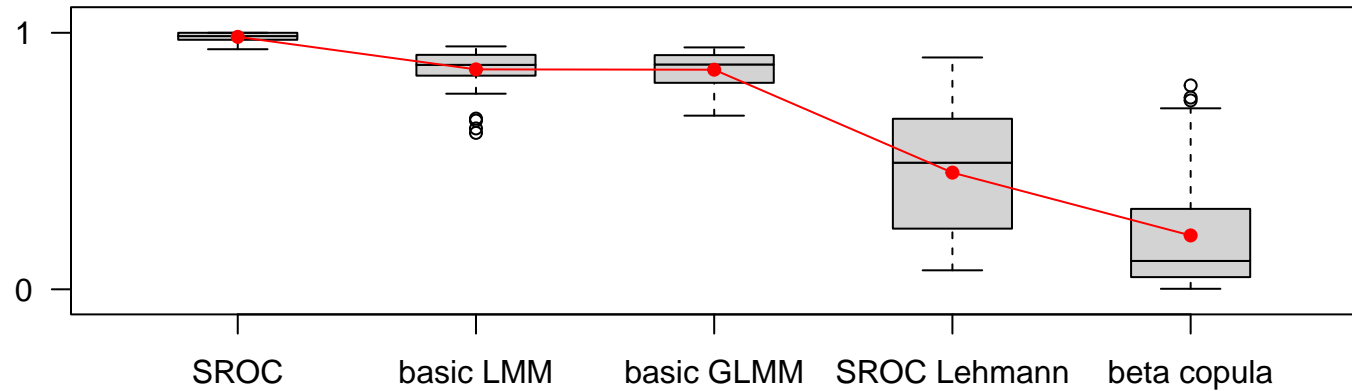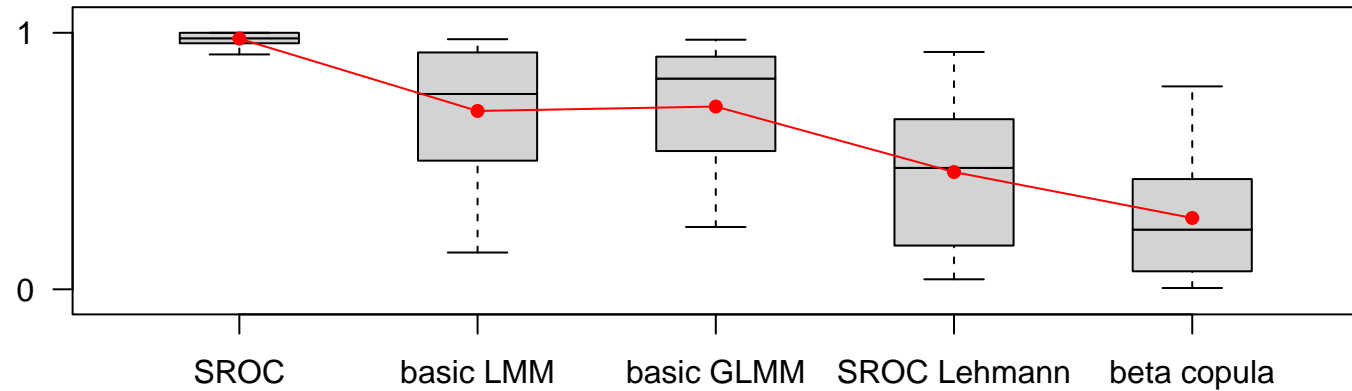

Supplement: Supplementary file 3 — Supporting File 3: bimj70147‐sup‐0003‐simstudy_code.zip. [file BIMJ-68-e70147-s001.zip › figures/Fig_S18_mob1t4.pdf]

specificity coverage

weight standard threshold

0

0.7

n = 192

n = 192

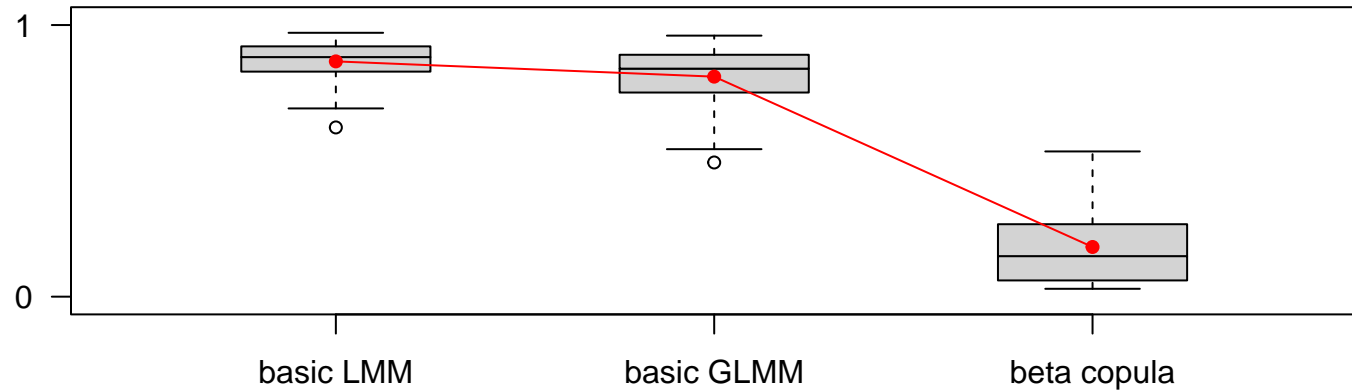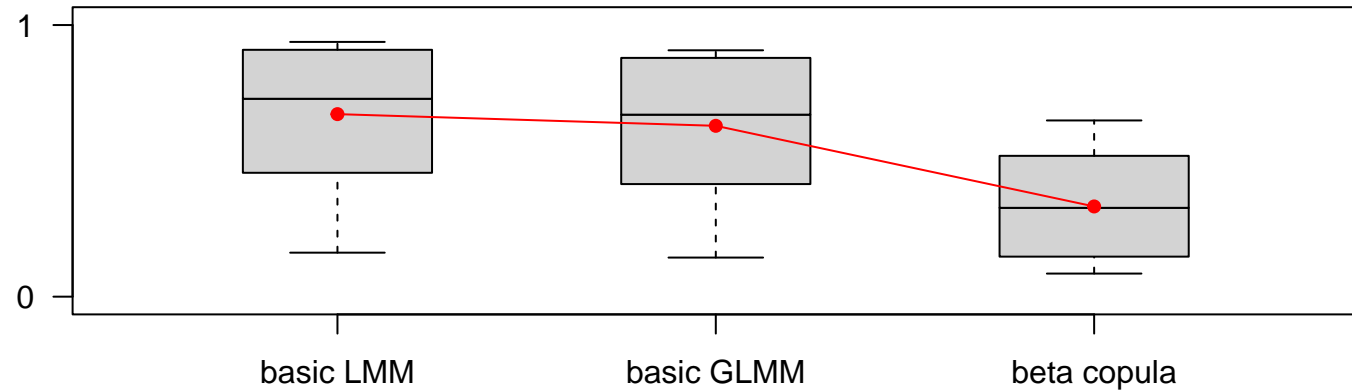

Supplement: Supplementary file 3 — Supporting File 3: bimj70147‐sup‐0003‐simstudy_code.zip. [file BIMJ-68-e70147-s001.zip › figures/Fig_S18_mob1t5.pdf]

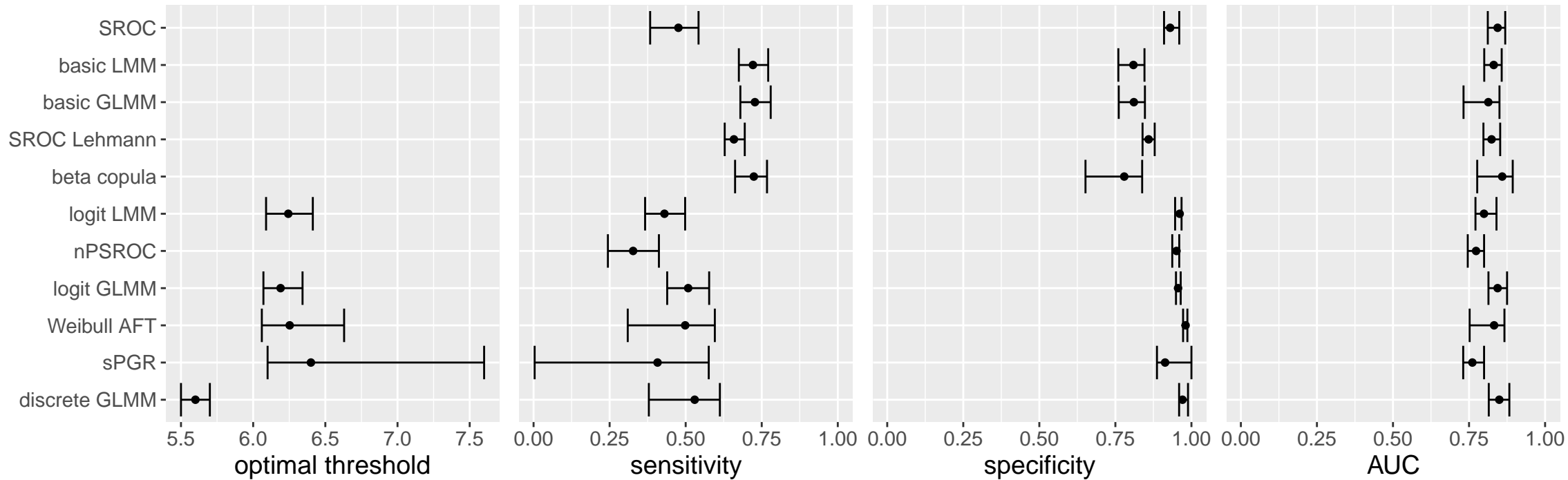

Supplement: Supplementary file 3 — Supporting File 3: bimj70147‐sup‐0003‐simstudy_code.zip. [file BIMJ-68-e70147-s001.zip › figures/Fig_S19_forest_plots_hba1c_all_w_sens_0.2.pdf]

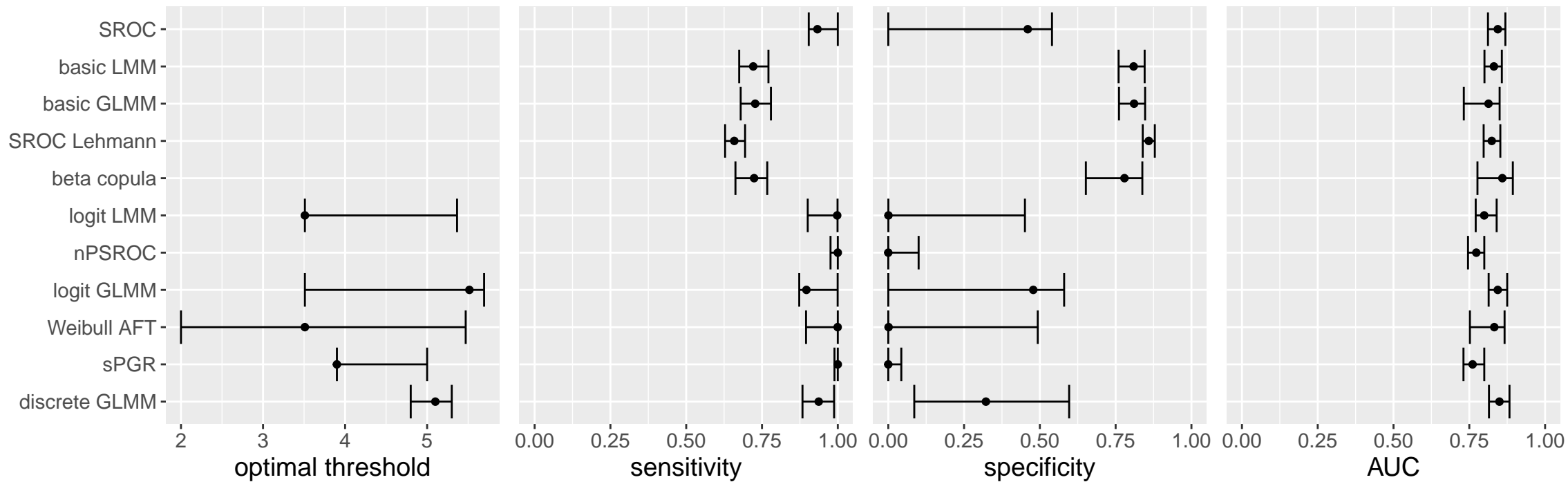

Supplement: Supplementary file 3 — Supporting File 3: bimj70147‐sup‐0003‐simstudy_code.zip. [file BIMJ-68-e70147-s001.zip › figures/Fig_S20_forest_plots_hba1c_all_w_sens_0.8.pdf]

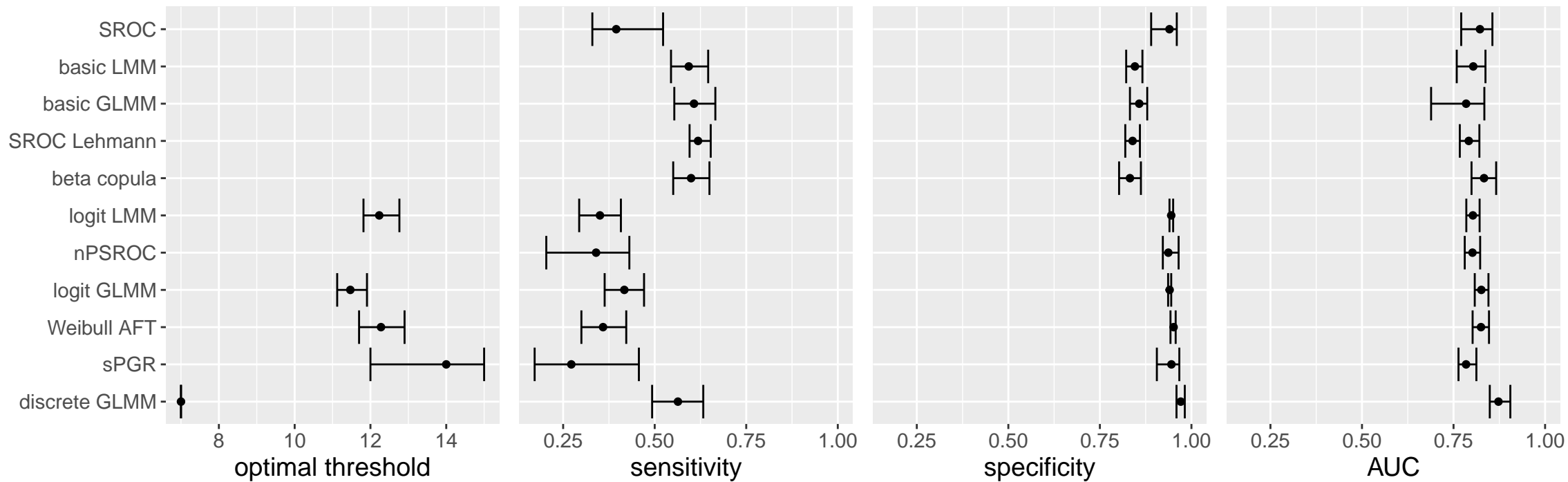

Supplement: Supplementary file 3 — Supporting File 3: bimj70147‐sup‐0003‐simstudy_code.zip. [file BIMJ-68-e70147-s001.zip › figures/Fig_S21_forest_plots_hadsa_all_w_sens_0.2.pdf]

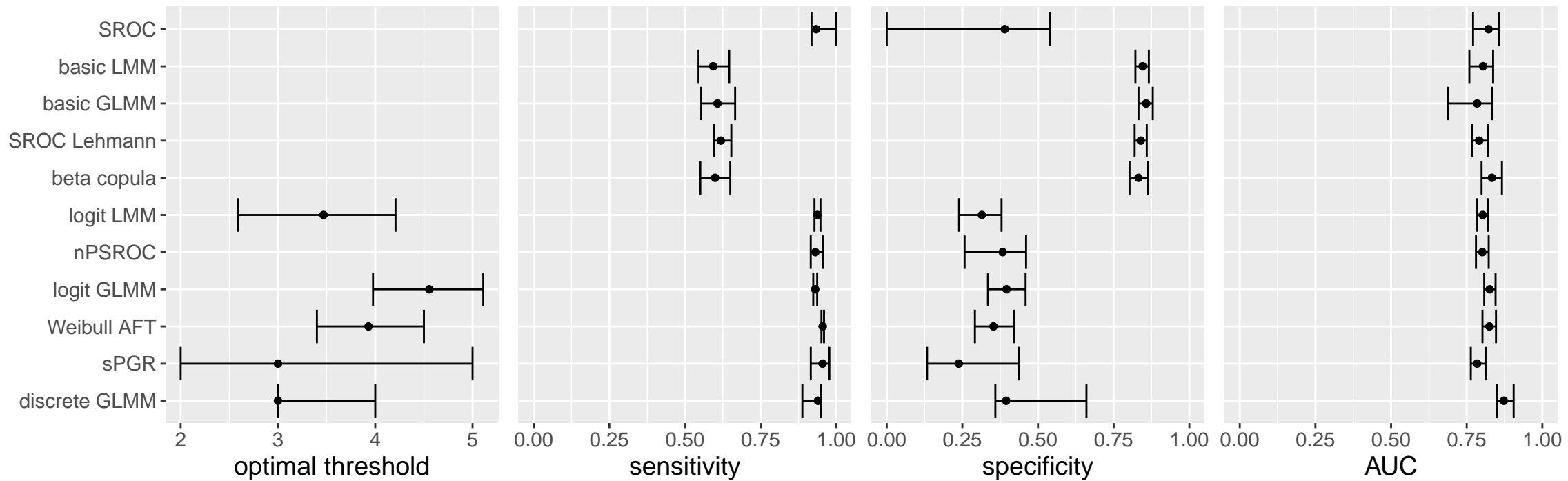

Supplement: Supplementary file 3 — Supporting File 3: bimj70147‐sup‐0003‐simstudy_code.zip. [file BIMJ-68-e70147-s001.zip › figures/Fig_S22_forest_plots_hadsa_all_w_sens_0.8.pdf]
