# Supplementary material for: Comparison of Different Methods for the Meta‐Analysis of Diagnostic Test Accuracy Studies—A Simulation Study
Source: Biom J. 2026 Jul 2;68(4):e70147. doi: 10.1002/bimj.70147 (PMC13329219; doi:10.1002/bimj.70147)
Supplement: Supplementary file 3 — Supporting File 3: bimj70147‐sup‐0003‐simstudy_code.zip. [file BIMJ-68-e70147-s001.zip › ressources/metaROC-simstudy.tar.gz!/metaROC-simstudy/tests/testthat/Rplots.pdf]

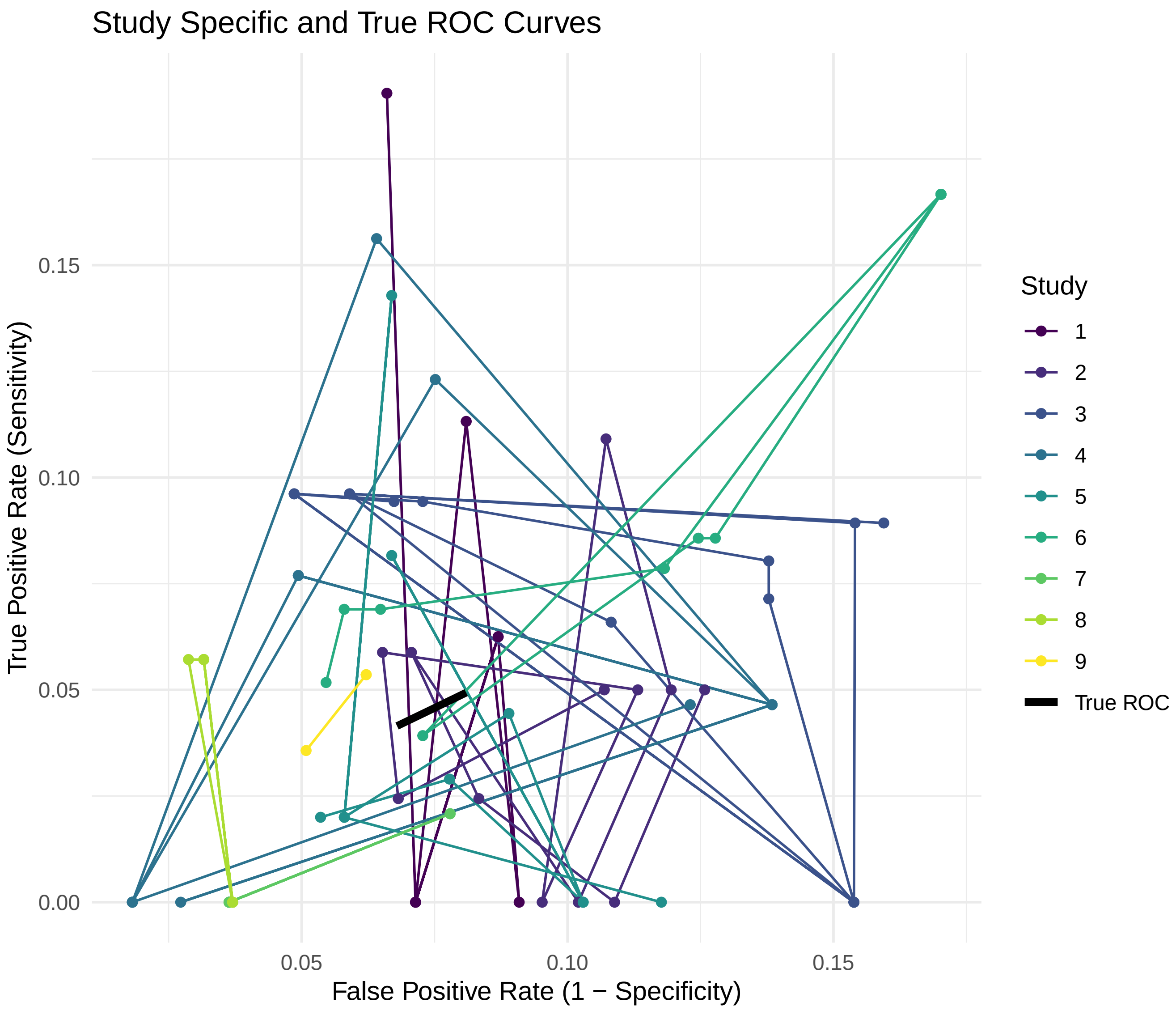

Study Specific and True ROC Curves
True Positive Rate (Sensitivity)
False Positive Rate (1 − Specificity)
0.00
0.05
0.10
0.15
0.05
0.10
0.15
Study
1
2
3
4
5
6
7
8
9
True ROC

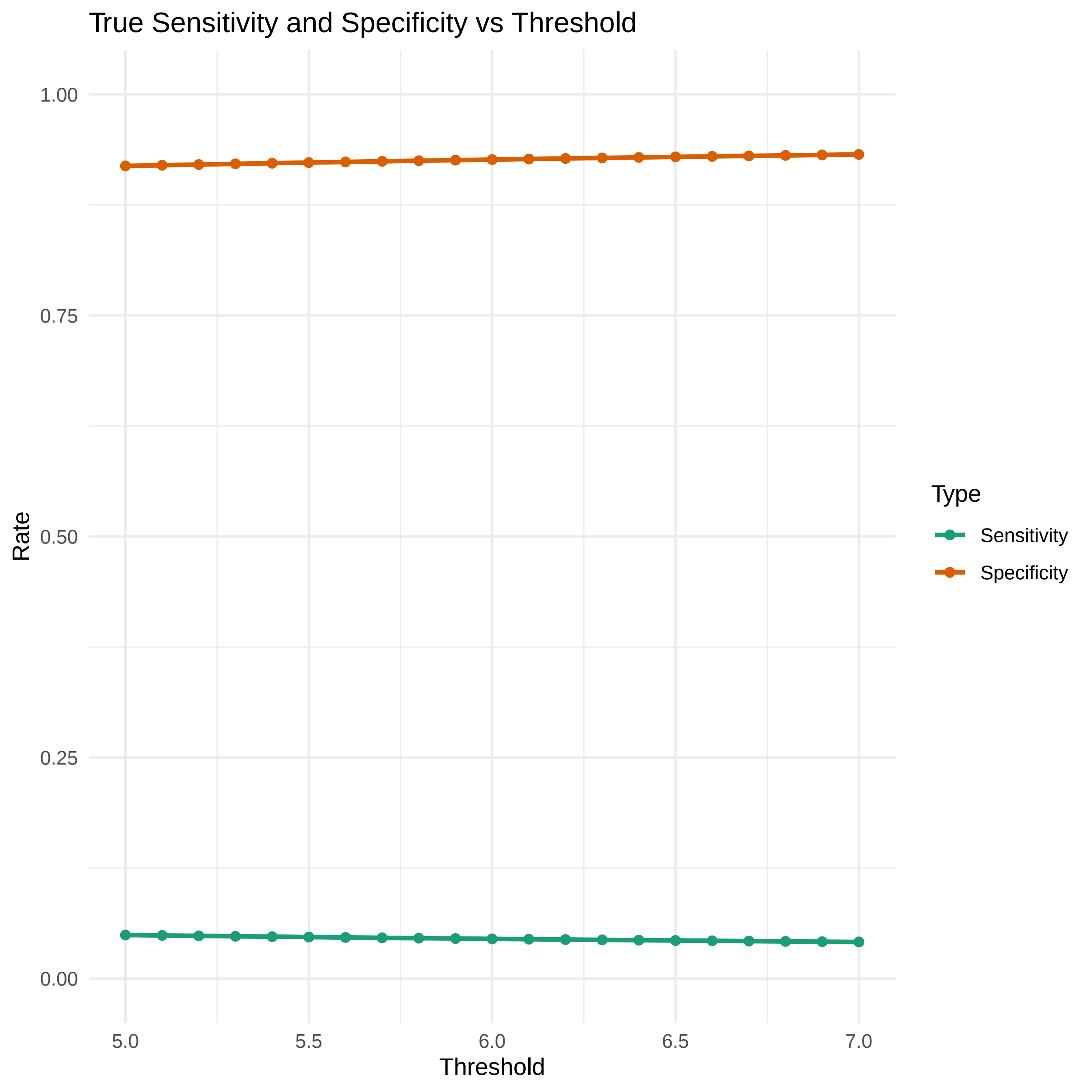
True Sensitivity and Specificity vs Threshold
1.00
0.75
0.50
0.25
0.00
Rate
5.0
5.5
6.0
6.5
7.0
Threshold
Type
Sensitivity
Specificity

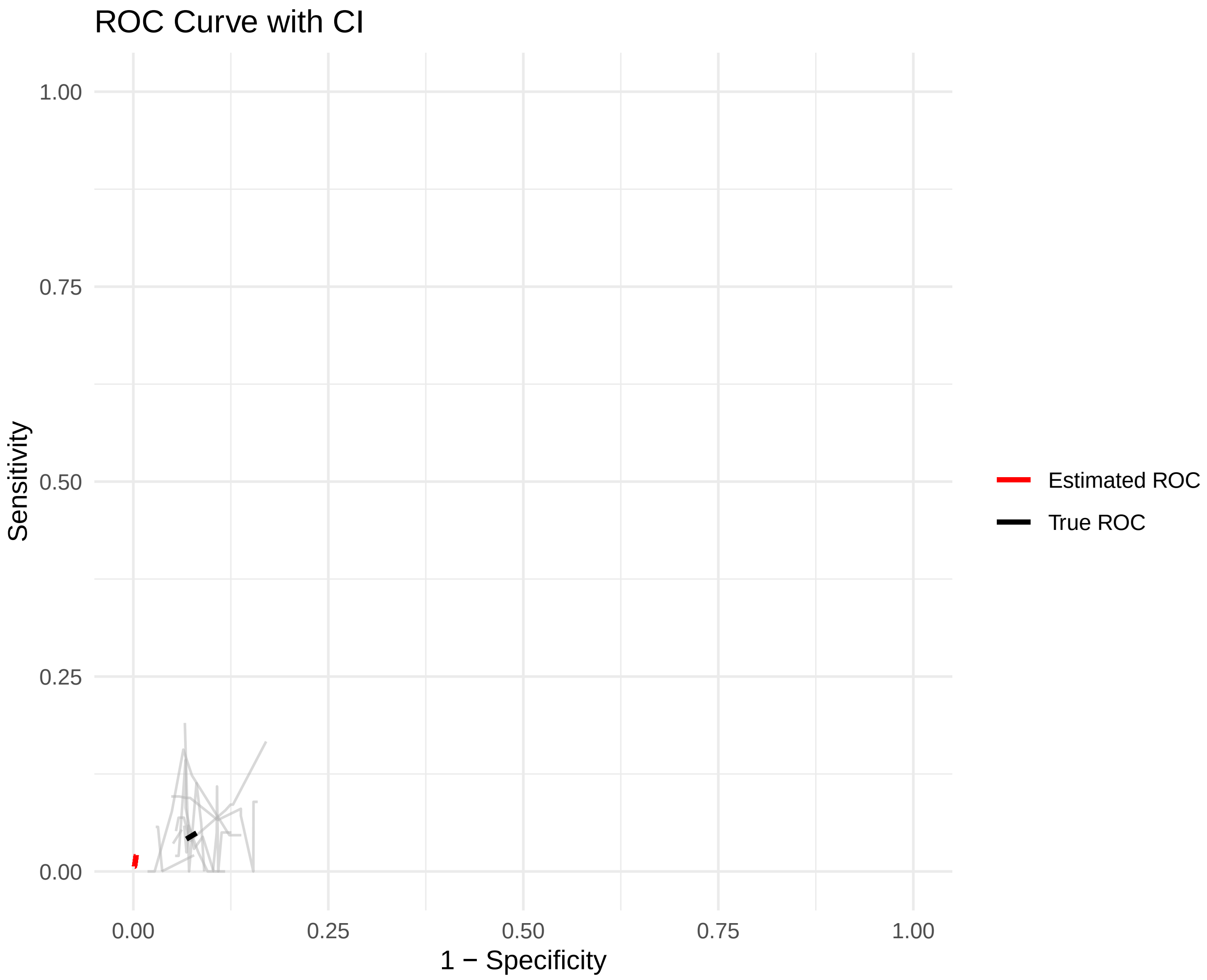

ROC Curve with CI
Sensitivity
1.00
0.75
0.50
0.25
0.00
0.00
0.25
0.50
0.75
1.00
1 − Specificity
Estimated ROC
True ROC

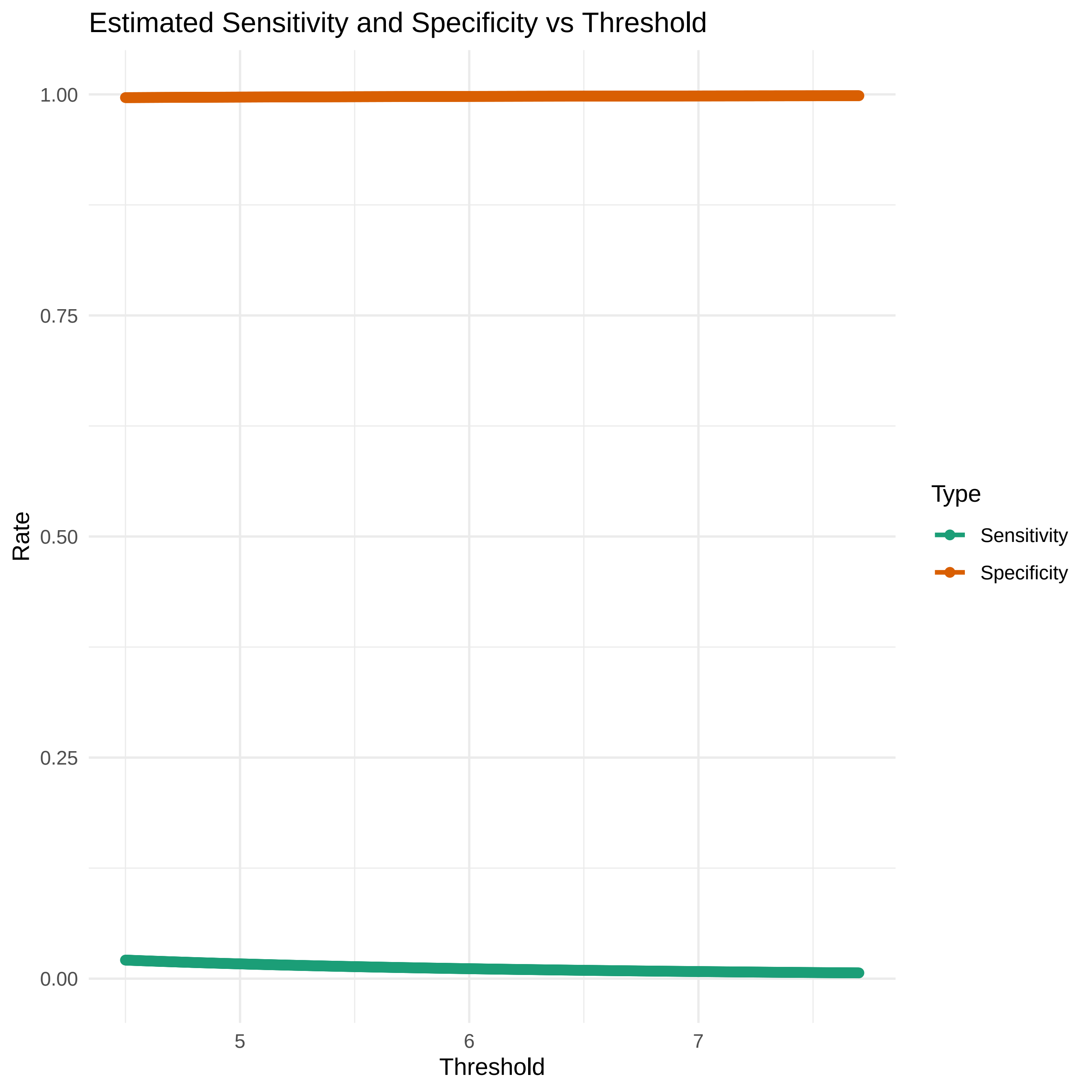

Estimated Sensitivity and Specificity vs Threshold
Rate
1.00
0.75
0.50
0.25
0.00
5
6
7
Threshold
Type
Sensitivity
Specificity

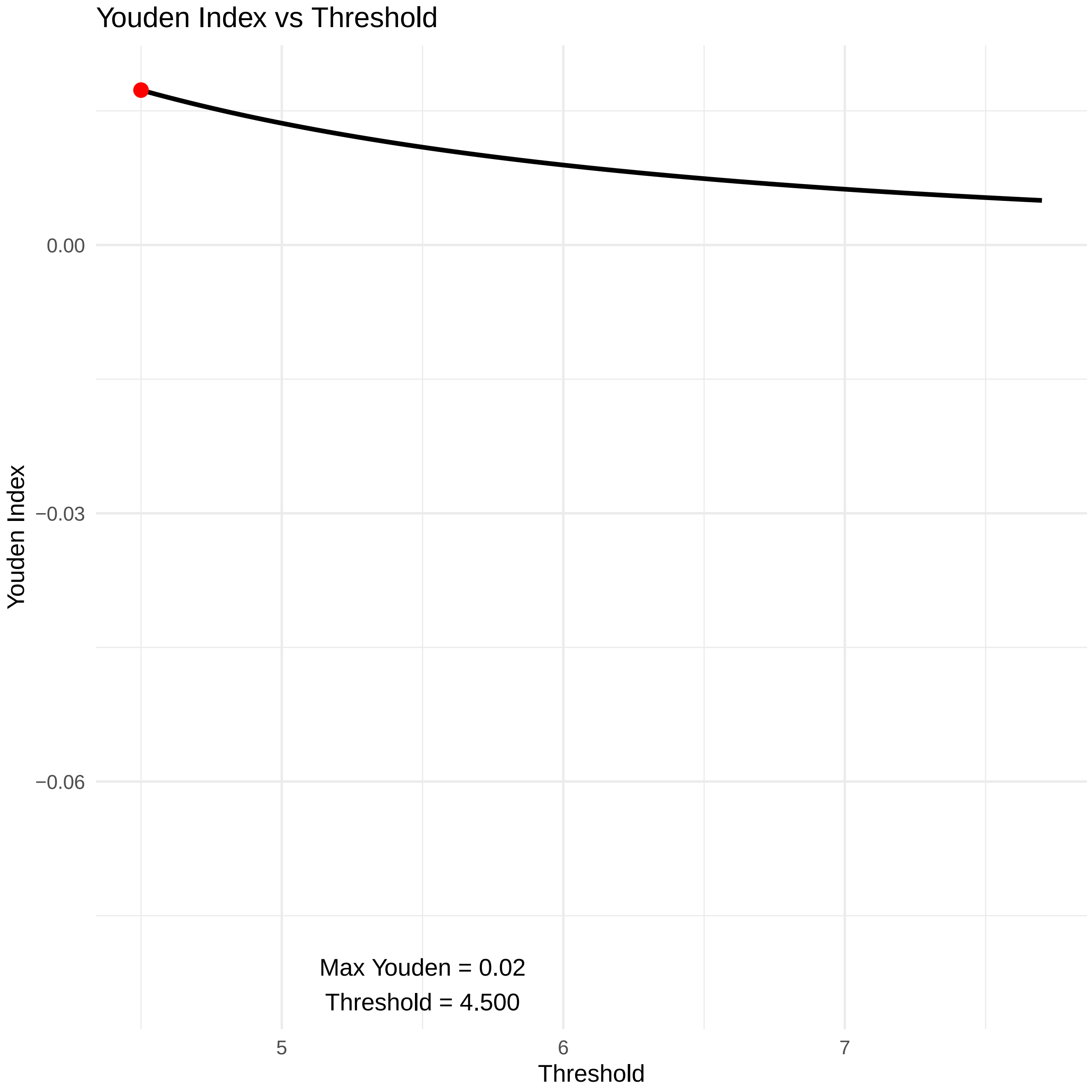

Youden Index vs Threshold
Youden Index
0.00
−0.03
−0.06
Max Youden = 0.02
Threshold = 4.500
5
6
7
Threshold

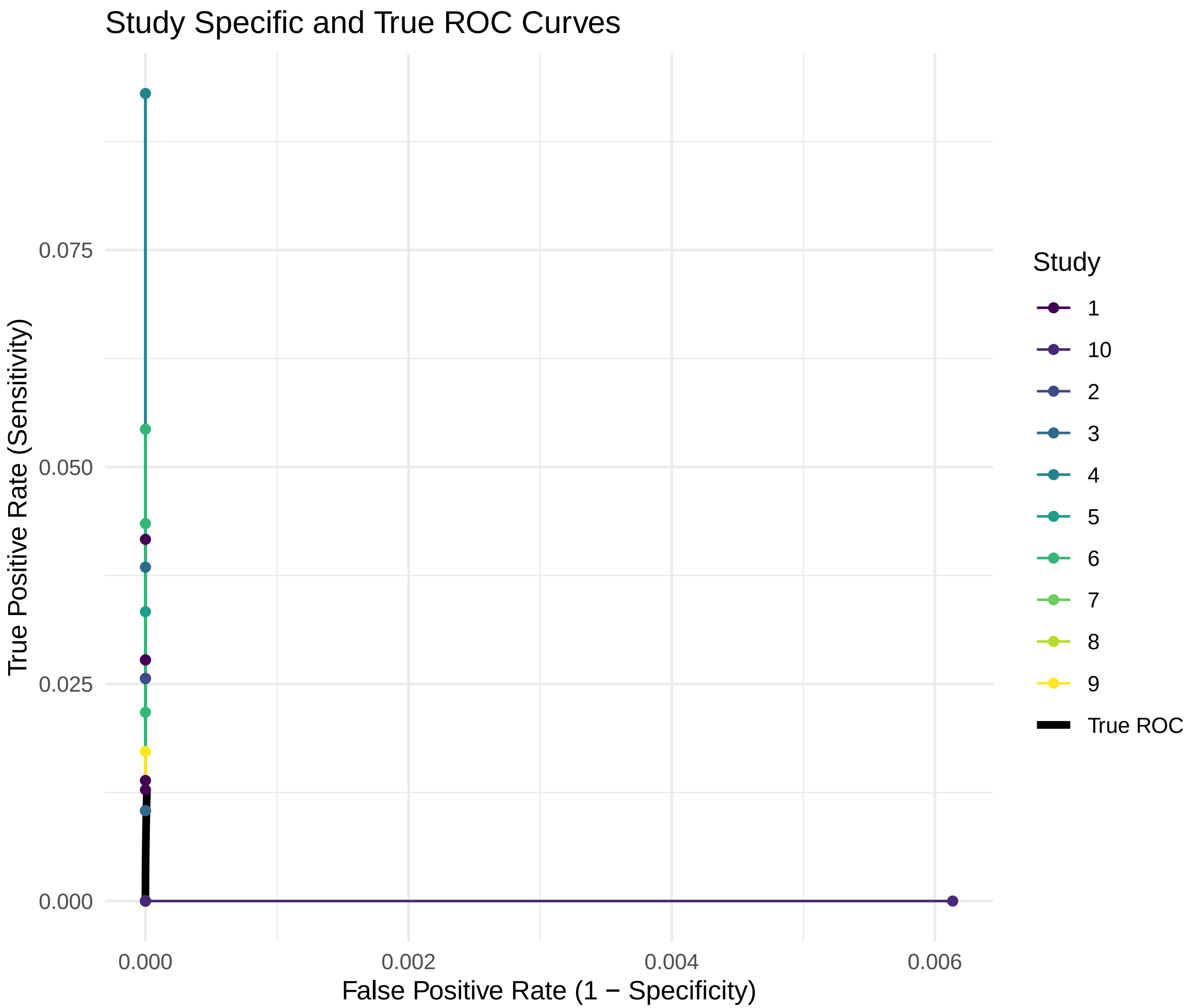

Study Specific and True ROC Curves
True Positive Rate (Sensitivity)
0.075
0.050
0.025
0.000
0.000
0.002
0.004
0.006
False Positive Rate (1 − Specificity)
Study
1
10
2
3
4
5
6
7
8
9
True ROC

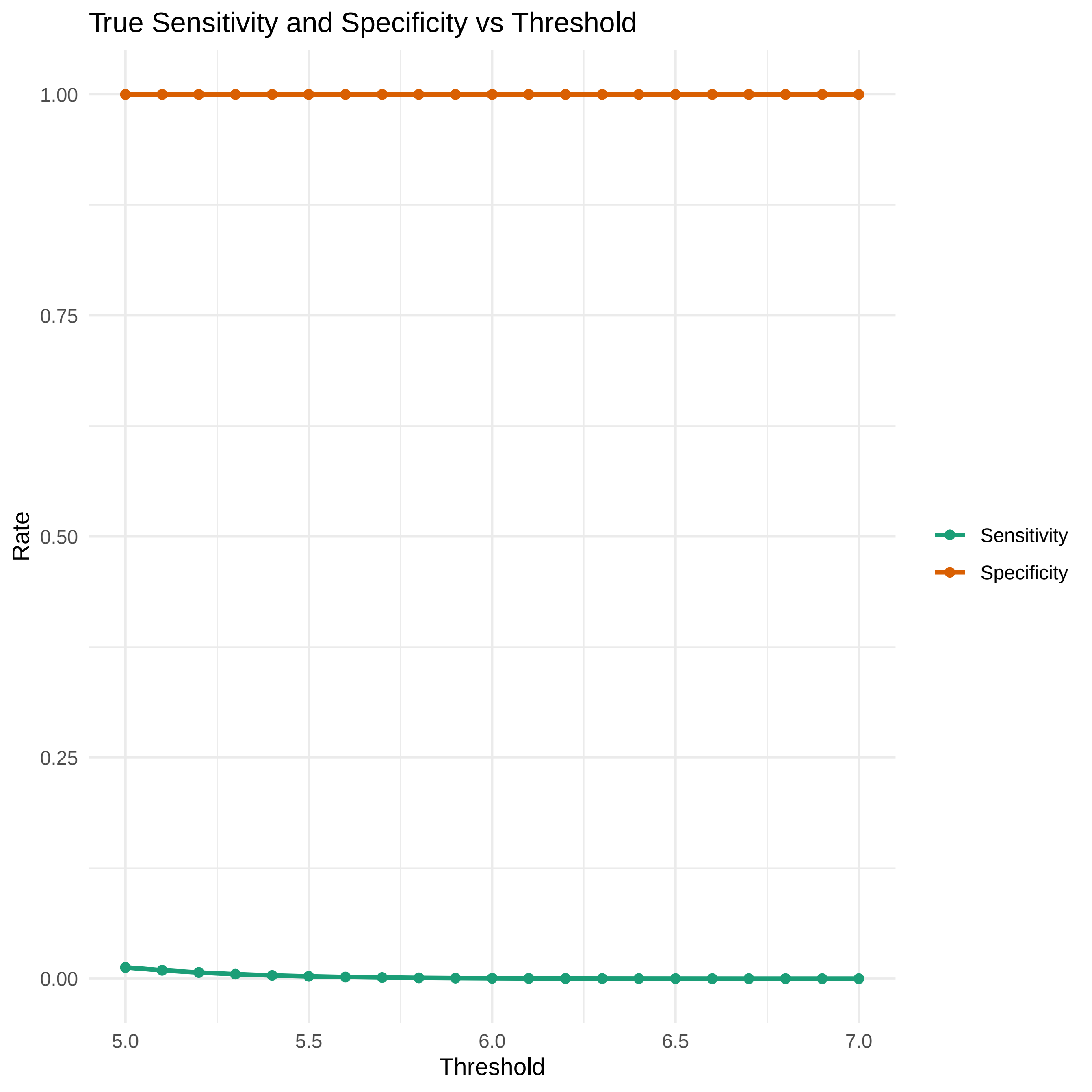
True Sensitivity and Specificity vs Threshold
Rate
1.00
0.75
0.50
0.25
0.00
Threshold
5.0
5.5
6.0
6.5
7.0
Sensitivity
Specificity
